# A Novel Virus Alters Gene Expression and Vacuolar Morphology in *Malassezia* Cells and Induces a TLR3-Mediated Inflammatory Immune Response

**DOI:** 10.1128/mBio.01521-20

**Published:** 2020-09-01

**Authors:** Minji Park, Yong-Joon Cho, Donggyu Kim, Chul-Su Yang, Shi Mun Lee, Thomas L. Dawson, Satoshi Nakamizo, Kenji Kabashima, Yang Won Lee, Won Hee Jung

**Affiliations:** a Department of Systems Biotechnology, Chung-Ang University, Anseong, South Korea; b The Research Institute of Basic Sciences, Seoul National University, Seoul, South Korea; c Department of Molecular and Life Science, Hanyang University, Ansan, South Korea; d Department of Bionano Technology, Hanyang University, Seoul, South Korea; e Singapore Immunology Network (SIgN), Agency for Science, Technology and Research (A*STAR), Biopolis, Singapore; f Center for Cell Death, Injury & Regeneration, Medical University of South Carolina, Charleston, South Carolina, USA; g Department of Dermatology, Kyoto University Graduate School of Medicine, Kyoto, Japan; h Department of Dermatology, School of Medicine, Konkuk University, Seoul, South Korea; i Research Institute of Medicine, Konkuk University, Seoul, South Korea; j Skin Research Institute of Singapore (SRIS), Agency for Science, Technology and Research (A*STAR), Biopolis, Singapore; k Department of Drug Discovery & Biomedical Sciences, Medical University of South Carolina, Charleston, South Carolina, USA; l Department of Biochemistry & Molecular Biology, Medical University of South Carolina, Charleston, South Carolina, USA; Texas Christian University

**Keywords:** *Malassezia*, *Malassezia restricta*, mycovirus, TLR3, cytokine, double-stranded RNA virus, virus, yeast virus

## Abstract

Most fungal viruses have been identified in plant pathogens, whereas the presence of viral particles in human-pathogenic fungi is less well studied. In the present study, we observed extrachromosomal double-stranded RNA (dsRNA) segments in various clinical isolates of *Malassezia* species. *Malassezia* is the most dominant fungal genus on the human skin surface, and species in this group are considered etiological factors of various skin diseases including dandruff, seborrheic dermatitis, and atopic dermatitis. We identified novel dsRNA segments, and our sequencing results revealed that the virus, named MrV40, belongs to the *Totiviridae* family and contains an additional satellite dsRNA segment encoding a novel protein. The transcriptome of virus-infected Malassezia restricta cells was compared to that of virus-cured cells, and the results showed that transcripts involved in ribosomal biosynthesis were downregulated and those involved in energy production and programmed cell death were upregulated. Moreover, transmission electron microscopy revealed significantly larger vacuoles in virus-infected *M. restricta* cells, indicating that MrV40 infection dramatically altered *M. restricta* physiology. Our analysis also revealed that viral nucleic acid from MrV40 induced a TLR3 (Toll-like receptor 3)-mediated inflammatory immune response in bone marrow-derived dendritic cells, suggesting that a viral element contributes to the pathogenicity of *Malassezia*.

## Abstract (precis)

*Malassezia* is the most dominant fungal genus on the human skin surface and is associated with various skin diseases including dandruff and seborrheic dermatitis. Among *Malassezia* species, *Malassezia restricta* is the most widely observed species on the human skin. In the current study, we identified a novel dsRNA virus, named MrV40, in *M. restricta* and characterized the sequence and structure of the viral genome along with an independent satellite dsRNA viral segment. Moreover, expression of genes involved in ribosomal synthesis and programmed cell death was altered, indicating that virus infection affected the physiology of the fungal host cells. Our data also showed that the viral nucleic acid from MrV40 induces a TLR3-mediated inflammatory immune response in bone marrow-derived dendritic cells, indicating that a viral element likely contributes to the pathogenicity of *Malassezia*. This is the first study to identify and characterize a novel mycovirus in *Malassezia*.

## INTRODUCTION

Viruses have been observed in many fungal species since their first identification in mushrooms (1). Fungal viruses, also known as mycoviruses, possess different forms of viral genomes including double-stranded RNA (dsRNA), single-stranded RNA (ssRNA), and single-stranded DNA (ssDNA). It is estimated that 30 to 80% of all fungal species, mainly endophytic fungi, are infected with viruses. Unlike viruses that infect other organisms, the transmission of fungal virus occurs vertically by cell division or horizontally via mating or hyphal anastomosis, with no extracellular phase of the virus life cycle. dsRNA segments have predominantly been found in fungal viruses, and taxonomically, the fungal dsRNA viruses are classified into seven families: *Chrysoviridae*, *Endornaviridae*, *Megabirnaviridae*, *Quadriviridae*, *Partitiviridae*, *Reoviridae*, and *Totiviridae* (2).

The model fungus Saccharomyces cerevisiae also carries a dsRNA virus that belongs to the *Totiviridae* family and is known as the L-A virus. A unique feature of fungal viruses of the *Totiviridae* family is their capability to produce a killer toxin that lyses susceptible neighbor strains, whereas the virus-containing strain (also known as the killer strain) is immune to the toxin. Studies of how the virus produces killer toxins in S. cerevisiae showed that the killer toxins are encoded by a satellite dsRNA segment, known as the M satellite, within the L-A virus. To date, four different killer toxins, K1, K2, K28, and Klus, have been described (3–6). The S. cerevisiae L-A virus forms icosahedral particles with a diameter of approximately 39 nm (7). The virus possesses a nonsegmented 4.6-kb dsRNA genome consisting of two open reading frames (ORFs), *gag* and *pol*, which overlap by 130 bp (8). A major 76-kDa capsid protein is encoded by *gag*, and a 180-kDa minor protein species is encoded as a Gag-Pol fusion protein by a −1 ribosomal frameshift event (9, 10). The C terminus of the Gag-Pol fusion protein possesses viral RNA-dependent RNA polymerase (RDRP) activity (8). The ribosomal frameshift is an interesting feature in a compact viral genome and has been commonly found in various viral genomes as a mechanism to allow viruses to express overlapping ORFs. Studies of S. cerevisiae L-A virus revealed that the mechanism of frameshifting is based on the sequence structures including a canonical slippery heptamer, 5′-X XXY YYZ-3′ (X = A, U, or G; Y = A or U; Z = A, U, or C) and RNA pseudoknot (11).

Fungal viruses have also been considered biocontrol agents in the field of agriculture. For example, a virus causes hypovirulence in the chestnut blight fungus Cryphonectria parasitica (12, 13), and a virus mediates the biocontrol of other phytopathogenic fungi such as Helminthosporium victoriae, Sclerotinia sclerotiorum, and Botrytis cinerea (14).

Although viral infections in fungal cells are widespread, the interactions between the fungal virus and its host are not well understood. One of the best-studied host defense mechanisms is RNA silencing. Several studies have shown that RNA silencing functions as an antiviral defense mechanism against *C. parasitica* in fungi. Disruption of the dicer pathway in *C. parasitica* increases the susceptibility to virus infections (15), and p29 was identified as a suppressor that inhibits expression of the genes required for RNA silencing-mediated viral defense in the fungus (16). Similarly, conserved RNA silencing-mediated antiviral defense systems have been identified in Aspergillus nidulans, Rosellinia necatrix, and Fusarium graminearum (17–19).

*Malassezia* is the most dominant fungal genus on the human skin surface and is considered an etiological factor in various skin diseases including dandruff, seborrheic dermatitis, and atopic dermatitis (20–23). Eighteen *Malassezia* species have been identified; among them, Malassezia restricta is the most abundant on the human skin (20). Recent studies showed an increased burden of *M. restricta* on the scalp of patients with dandruff, indicating an association between dandruff and the fungus, although its role as a pathogenic organism is still unclear, and host susceptibility should be taken into consideration (21, 24–27). Most fungal viruses are found in plant-pathogenic fungi, whereas few examples of viral particles have been identified in human-pathogenic fungi such as Candida albicans (28). In the present study, we observed extrachromosomal dsRNA segments in various *M. restricta* clinical isolates which represented a novel viral genome and its satellite. Sequence analysis revealed that the virus belongs to the *Totiviridae* family and that the additional satellite dsRNA segment encodes a novel protein. The interactions between the viral elements and the fungal host and the impact of the virus on fungal interactions with immune cells were evaluated.

## RESULTS

### Identification of extrachromosomal dsRNA segments in *Malassezia*.

**FIG 1:**
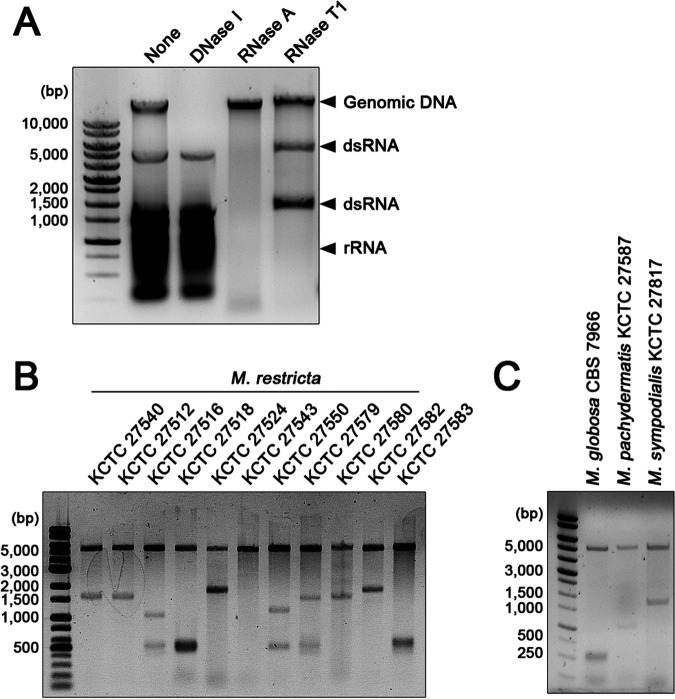
Extrachromosomal dsRNA segments in *Malassezia*. (A) Nucleic acids from *Malassezia restricta* KCTC 27540 were separated on a 0.7% agarose gel. Lane 1, total nucleic acids; lane 2, total nucleic acids treated with DNase I; lane 3, total nucleic acids treated with RNase A; lane 4, total nucleic acids treated with RNase T_1_. (B) Presence of dsRNA in *M. restricta* strains. Total nucleic acids were extracted and treated with DNase I and RNase T_1_ and were separated on a 0.7% agarose gel. (C) Total nucleic acids were extracted from different *Malassezia* species, treated with DNase I and RNase T_1_, and separated on a 0.7% agarose gel.

Extrachromosomal nucleic acid bands were observed in total nucleic acid extracts of the *M. restricta* strains isolated in our recent study (29). Among the strains, *M. restricta* KCTC 27540 was used to identify extrachromosomal segments. Total nucleic acids were extracted from the strain and digested with DNase I, RNase A, and RNase T_1_. The extrachromosomal segments and rRNA were resistant to DNase I, indicating that they were neither ssDNA nor dsDNA. RNase A degraded all nucleic acids except for genomic DNA, whereas RNase T_1_, which catalyzes the degradation of ssRNA, removed rRNA only (30). These results suggested that the extrachromosomal segments correspond to dsRNA and that *M. restricta* KCTC 27540 possesses two separate extrachromosomal segments as estimated by agarose gel electrophoresis to be approximately 4.5 and 1.5 kb (Fig. 1A).

To confirm whether the extrachromosomal segments observed in other *M. restricta* clinical isolates were also dsRNA, total nucleic acid extracts from strains other than *M. restricta* KCTC 27540 were treated with DNase I and RNase T_1_. The extrachromosomal segments remained unaffected after enzyme treatment, indicating that they are also dsRNA, as in *M. restricta* KCTC 27540 (Fig. 1B). Moreover, other *Malassezia* species including M. globosa, M. pachydermatis, and M. sympodialis showed similar extrachromosomal dsRNA segments, suggesting that these segments are common characteristics of *Malassezia* species (Fig. 1C). Agarose gel electrophoresis revealed extrachromosomal segments composed of at least two separate dsRNA fragments except for *M. restricta* KCTC 27543, which showed a single dsRNA fragment. Additionally, the large fragments of dsRNA showed similar sizes (∼5.0 kb), whereas the small dsRNA segments varied in size in different strains (Fig. 1B and C). We hypothesized that the dsRNA segments from *Malassezia* strains represent the dsRNA elements of mycoviruses, which are prevalent in all major fungal taxa (2). Indeed, a dsRNA mycovirus-infected soil fungus, Trichoderma atroviride NFCF028, displayed similar extrachromosomal segments (see Fig. S1 in the supplemental material) (31).

10.1128/mBio.01521-20.2 FIG S1 Extrachromosomal dsRNA segments in *Malassezia restricta* KCTC 27540 and T. atroviride NFCF028. Nucleic acids from the strains were separated on a 0.7% agarose gel. Lane 1, total nucleic acids; lane 2, total nucleic acids treated with DNase I; lane 3, total nucleic acids treated with RNase A; lane 4, total nucleic acids treated with RNase T_1_. Download FIG S1, PDF file, 0.3 MB. Copyright © 2020 Park et al. 2020 Park et al. This content is distributed under the terms of the Creative Commons Attribution 4.0 International license.

**FIG 2:**
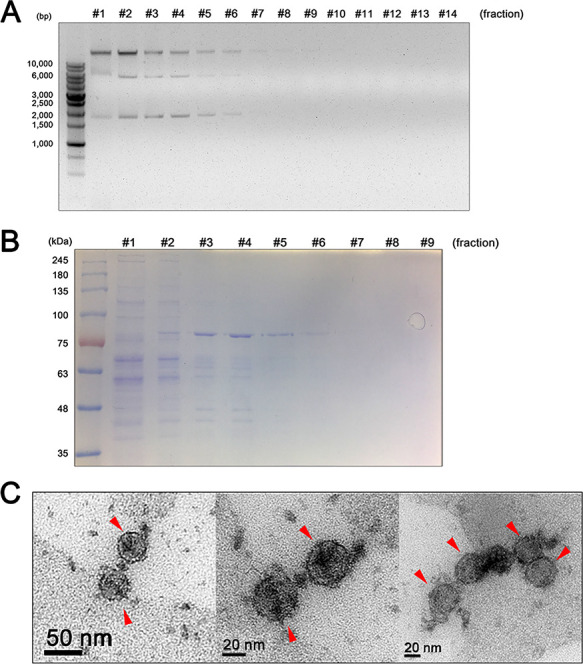
Isolation of virus particles from *Malassezia restricta* KCTC 27540 and their TEM images. The collected fractions after sucrose gradient ultracentrifugation were analyzed for their nucleic acids and proteins on an agarose gel (A) and SDS-PAGE gel (B), respectively. Images of the viral particles (red arrowheads) were obtained using a transmission electron microscope (C).

**FIG 3:**
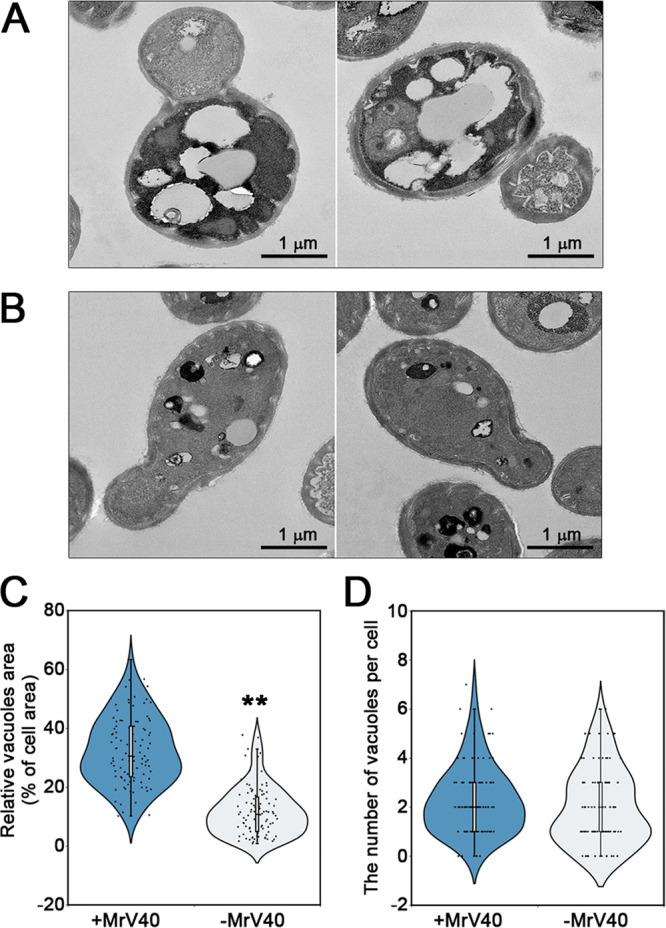
Morphology of vacuoles in virus-infected and virus-cured *Malassezia restricta* cells. Ultrathin sections of virus-infected (A) and virus-cured (B) *M. restricta* KCTC 27540 strains were observed by TEM. Cytoplasmic vacuole formation in a cell, expressed as the percentage of the area occupied by vacuoles (C) and the number of vacuoles (D) per cell, was estimated (*n* = 100 cells for each strain). The vacuole area was estimated using ImageJ (https://imagej.nih.gov/ij/), and the plot was created with BioVinci software (BioTuring Inc., San Diego, CA, USA). Statistical analysis for differences between the strains was performed with unpaired *t* tests. **, *P* < 0.001.

Sucrose gradient ultracentrifugation was conducted to purify virus particles to confirm that the dsRNA segments in the *Malassezia* strains were indeed viral elements. The separated nucleic acids and proteins in each fraction were analyzed. Two dsRNA fragments (∼5.0 and ∼1.7 kb) were clearly visible in fractions 1 to 6 following agarose gel electrophoresis (Fig. 2A). Moreover, the results of sodium dodecyl sulfate-polyacrylamide gel electrophoresis (SDS-PAGE) showed that fractions 3 to 6 contained protein bands with an estimated molecular weight of ∼77 kDa (Fig. 2B). This molecular weight is similar to that of the known capsid protein of the S. cerevisiae mycovirus (9, 32, 33). Fractions 3 to 6 were subsequently evaluated by microscopy to visualize mycovirus particles in *M. restricta*. Transmission electron microscopy (TEM) images showed virus-like particles with an isometric shape and a diameter of 43 nm (Fig. 2C). These results support the hypothesis that the extrachromosomal dsRNA segments form the genome of mycovirus in *M. restricta* KCTC27540. We named the viral particle MrV40 (*M. restricta* KCTC 27540 mycovirus). The large and small dsRNA viral fragments were named MrV40L and MrV40S, respectively. In addition to evaluating images of the purified virus particles, we examined the morphology of the virus-infected and the virus-cured *M. restricta* KCTC 27540 strain pairs by TEM. The virus-cured *M. restricta* KCTC 27540 strain was prepared by sterilizing the virus-infected *M. restricta* KCTC 27540 through numerous serial passages (Text S1 and Fig. S2). In general, there was no morphological difference between the virus-infected and the virus-cured KCTC 27540 strains. However, we noticed that the size of vacuoles in the virus-infected strain was significantly larger than that in the virus-cured strain, suggesting that the virus influences vacuole size in *M. restricta* (Fig. 3).

10.1128/mBio.01521-20.1 TEXT S1 Supplemental materials and methods. Cell fixation, transmission electron microscopy (TEM), cDNA synthesis, RNA isolation and transcriptome analysis by RNA sequencing, quantitative RT-PCR (Q-RT-PCR), annexin V-FITC/propidium iodide (PI) staining and flow cytometric analysis, heterologous expression and purification of the capsid protein, heterologous expression and purification of the MrV40S satellite proteins, and preparation of fungal cell lysates. Download Text S1, PDF file, 0.1 MB. Copyright © 2020 Park et al. 2020 Park et al. This content is distributed under the terms of the Creative Commons Attribution 4.0 International license.

10.1128/mBio.01521-20.3 FIG S2 Confirmation of the virus-cured *Malassezia restricta* strain. Total nucleic acids from the virus-infected and the virus-cured *M. restricta* KCTC 27540 and KCTC 27524 strains were extracted and treated with RNase T_1_. The dsRNA viral segments were not detected in the virus-cured *M. restricta* KCTC 27540 and KCTC 27524 strains (lane 2 and lane 4, respectively). Download FIG S2, PDF file, 0.3 MB. Copyright © 2020 Park et al. 2020 Park et al. This content is distributed under the terms of the Creative Commons Attribution 4.0 International license.

### Determination of dsRNA sequence of MrV40L.

**FIG 4:**
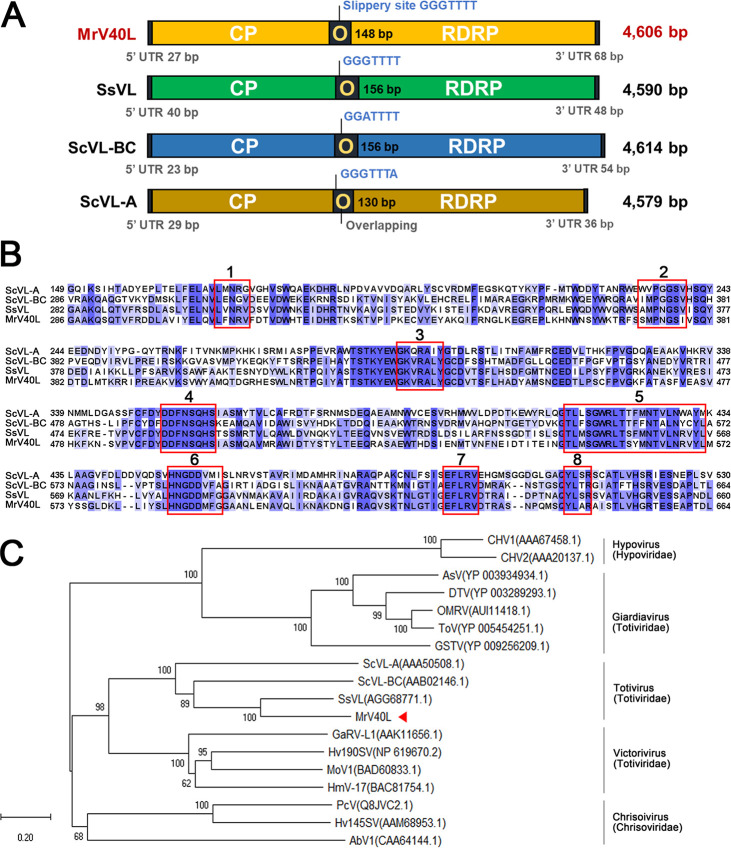
Genome and phylogenetic analysis of MrV40L. (A) Comparison of the genomic organization of MrV40L with that of other totiviruses. (B) Eight conserved motifs within the viral RDRP of MrV40L. The red boxes indicate conserved motifs. The amino acid sequences of ScVL-A, ScVL-BC, SsVL, and MrV40L were aligned using Jalview. (C) Multiple alignment of 18 amino acid sequences of RDRPs from dsRNA viruses was analyzed using the neighbor-joining method with bootstrap test (1,000 replicates) using MEGA X (81, 83, 84). The evolutionary distances are in units of the number of amino acid substitutions per site. All ambiguous positions were removed for each sequence pair. CHV1, *Cryphonectria* hypovirus 1; CHV2, *Cryphonectria* hypovirus 2; AsV, *Armigeres subalbatus* virus; DTV, Drosophila melanogaster totivirus; OMRV, Omono River virus; ToV, Tianjin totivirus; GSTV, *Golden shiner* totivirus; ScVL-A, S. cerevisiae virus L-A; ScVL-BC, S. cerevisiae virus L-BC; SsVL, *S. segobiensis* virus L; GaRV-L1, *Gremmeniella abietina* virus L1; Hv190SV, *Helminthosporium victoriae* virus-190S; MoV1, Magnaporthe oryzae virus 1; HmV-17, *Helicobasidium mompa* totivirus 1-17; PcV, Penicillium chrysogenum virus; Hv145SV, *Helminthosporium victoriae* 145S virus; AbV1, *Agaricus bisporus* virus 1 (8, 33, 34, 85–97).

The complete sequence of MrV40L was determined by a combination of the Illumina MiSeq technique and the Sanger sequencing method using purified viral dsRNA. The length of the complete assembled sequence of MrV40L was 4,606 bp, and two overlapping open reading frames (ORFs), designated ORF1 and ORF2, were identified (Fig. 4A). ORF1 corresponds to the region from nucleotides (nt) 28 to 2097 and encodes a polypeptide of 689 amino acids with a molecular weight of 77 kDa. ORF2 corresponds to the region from nt 1949 to 4538 and encodes a polypeptide of 862 amino acids with a molecular weight of 98 kDa. The results of BLAST analysis showed that the protein sequences of ORF1 and ORF2 were highly similar to that of the capsid protein (the Pfam families of LA-virus_coat, PF09220) and viral RNA-directed RNA polymerase (RDRP; the Pfam family of RDRP_4, PF02123) of Scheffersomyces segobiensis virus L belonging to the genus *Totivirus* (*Totiviridae* family) with 53% (YP_009507830.1) and 52% (YP_009507831.1) identities, respectively (34). Eight conserved motifs, which are commonly found in totiviruses, were found within ORF2, supporting the classification of MrV40 as a totivirus (Fig. 4B) (35). Moreover, phylogenetic analysis with known amino acid sequences of RDRPs from dsRNA mycoviruses demonstrated that the RDRP encoded by the MrV40L genome was clustered with totiviruses (Fig. 4C). Thus, MrV40L is a dsRNA viral genome encoding a capsid protein and an RDRP, and we propose that MrV40 belongs to the *Totivirus* genus.

**FIG 5:**
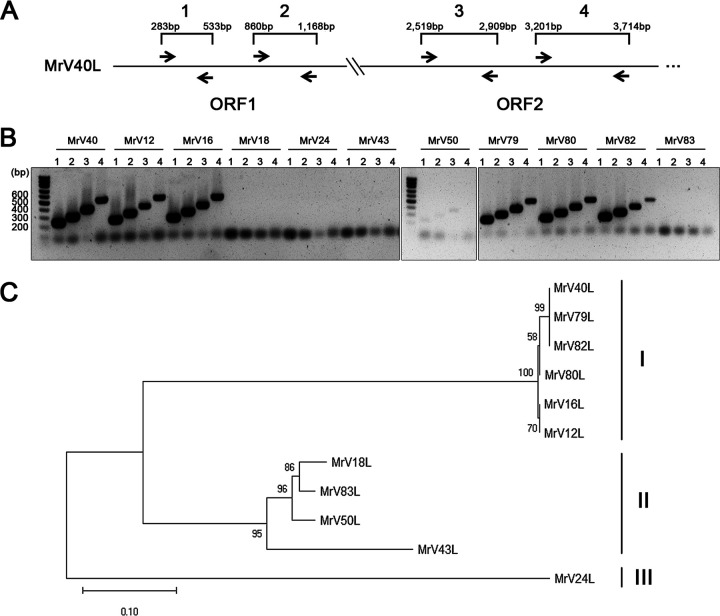
Phylogenetic classification of viruses found in *Malassezia restricta*. (A and B) The cDNAs of dsRNAs from *M. restricta* strains were amplified using primers for genes encoding CP and RDRP (ORF1 and ORF2, respectively) homologs in the viral genome. The following primers were used: lane 1, MrV40L_CP_F1 and MrV40L_CP_R1; lane 2, MrV40L_CP_F2 and MrV40L_CP_R2; lane 3, MrV40L_RDRP_F1 and MrV40L_RDRP_R1; lane 4, MrV40L_RDRP_F2 and MrV40L_RDRP_R2 (see Table S2 in the supplemental material). (C) MrV-Ls were clustered into three clades (clades I, II, and III). Multiple alignment of nucleotide sequences of combined parts of *gag* and *pol* from 11 MrV-Ls was analyzed by the neighbor-joining method with bootstrap test (1,000 replicates) in MEGA X (81, 83, 84). The evolutionary distances are in units of the number of base substitutions per site.

Next, we analyzed the genomic sequences of dsRNA viruses in other clinical *M. restricta* isolates to confirm that they were similar to those of MrV40L and thus belong to the *Totivirus* genus. Reverse transcription-PCR (RT-PCR) was performed using four primer sets corresponding to the sequence of the conserved regions of the capsid protein and RDRP in the MrV40L genome (Fig. 5A). The results showed that six of 11 viruses generated RT-PCR products, suggesting the presence of viral genomes similar to MrV40Ls, while the remaining samples had dissimilar viral genomes (Fig. 5B).

Furthermore, we performed phylogenetic classification of the same viruses found in other clinical *M. restricta* isolates by multilocus sequence typing of the 1,075-bp region, 638 bp of ORF1 and 437 bp of ORF2, corresponding to *gag* and *pol*, respectively, within the viral genomes. The results revealed that the viruses were classified into three clades: clade I (MrV12L, MrV16L, MrV40L, MrV79L, MrV80L, and MrV82L), clade II (MrV18L, MrV43L, MrV83L, and MrV50L), and clade III (MrV24L) (Fig. 5C). Additionally, the sequences of the 1,075-bp region of MrV40L, MrV79L, and MrV82L in clade I were 100% identical, suggesting that they originated from the same lineage.

### Determination of dsRNA sequence of MrV40S.

To determine the sequence of MrV40S, the dsRNA segments of MrV40S were extracted from agarose gels and then subjected to cDNA cloning and sequencing (see Materials and Methods). Using the partial sequences obtained from cDNA clones and the sequences obtained from repeated 5′ rapid amplification of cDNA ends (RACE), we successfully determined the complete sequence of MrV40S. The sequence of MrV40S was 1,355 bp, and a single ORF was identified in the 3′ region (from nt 773 to 1097), which encoded a polypeptide of 124 amino acids with a molecular weight of 15.6 kDa. The ORF was designated ORF3. Although we obtained the complete sequence of MrV40S ORF3, no homologous protein sequence was identified by BLAST analysis using all currently available databases.

**FIG 6:**
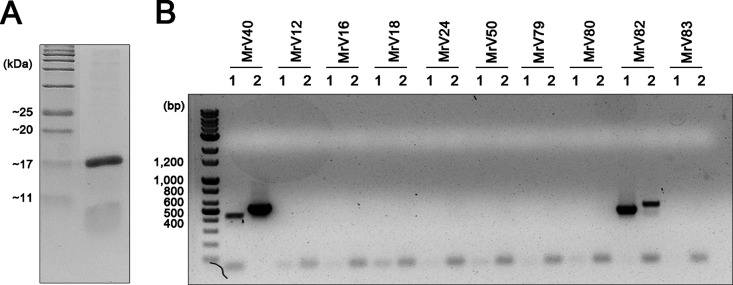
Characterization and similarity analysis of MrV40S. (A) The protein encoded by ORF3 was heterologously expressed in E. coli and purified using a His tag column. (B) RT-PCR results. The primers specific to MrV40S were used to amplify satellite dsRNA from other *M. restricta* strains containing small dsRNA segments. The following primers were used: lane 1, MrV40S_F5 and V40S_SP1; lane 2, MrV40S_ORF_F1 and MrV40S_R1 (see Table S2 in the supplemental material).

Mycoviruses belonging to *Totivirus*, particularly the S. cerevisiae L-A virus, often possess a satellite dsRNA segment known as M dsRNA, which is responsible for producing a killer toxin that excludes neighboring yeast cells. Because MrV40L resembles M dsRNA, we predicted that MrV40S produces a protein that inhibits other *Malassezia* strains and/or other fungal and/or bacterial cells residing on the skin. To test the toxin-like activity of the protein produced from MrV40S, ORF3 was cloned and the protein was heterologously expressed in Escherichia coli and purified (Fig. 6A). The activity of the purified protein was evaluated against several pathogenic fungi and bacteria including *M. restricta*, C. albicans, Cryptococcus neoformans, E. coli, and Staphylococcus aureus. Unexpectedly, the purified protein displayed no growth-inhibitory effect on the microbial cells tested (data not shown). Based on these results, we concluded that the novel protein produced from ORF3 likely has no toxin-like activity against the microorganisms tested, and further functional studies are required to characterize its function.

M dsRNA in S. cerevisiae produces several variants of killer toxins, namely, M1, M2, M3, and Mlus, and they showed limited homology in their protein sequences (4–6). Based on this information, we compared the sequences of satellite dsRNA genomes between MrV40S and those in other *M. restricta* strains containing the small dsRNA segments. RT-PCR was conducted using a series of primers specific to MrV40S. The results suggested that among the nine strains tested, only the satellite dsRNA in *M. restricta* KCTC 27582 possessed a similar dsRNA segment. These data indicate that the satellite dsRNA genome sequences are highly variable (Fig. 6B).

### MrV40 altered transcriptome profiles in the host *M. restricta* strain.

To understand the influence of MrV40 on the host physiology, we compared the transcriptomes of virus-infected and virus-cured *M. restricta* KCTC 27540 strain pairs by RNA sequencing. Transcriptome analysis showed that 258 and 809 genes were up- and downregulated by more than 2-fold, respectively, in the virus-infected strain compared to those in the virus-cured strain (Table 1 and Table S1). These results indicate that the presence of the virus may impact a number of physiological processes in *M. restricta*.

**TABLE 1:** Number of differentially expressed genes

Category	No. of differentially expressed genes (+virus/−virus)				
	2-fold	3-fold	4-fold	>5-fold	Total
Upregulated	204	32	12	10	258
Downregulated	597	152	70	60	809

10.1128/mBio.01521-20.7 TABLE S1 Expression of genes calculated from transcriptome sequencing (RNA-seq) data. Download Table S1, PDF file, 1.8 MB. Copyright © 2020 Park et al. 2020 Park et al. This content is distributed under the terms of the Creative Commons Attribution 4.0 International license.

In S. cerevisiae, numerous genes are known to be involved in maintaining its dsRNA virus (36) and are categorized into two groups: *MAK* (maintenance of killer) and *SKI* (superkiller). At least 25 *MAK* genes have been reported (37); among them, *MAK3*, *MAK10*, and *PET18* were shown to be required for the maintenance of both S. cerevisiae L-A virus and its M satellite, whereas all other *MAK* genes are responsible only for the M satellite (38–43). We could not find homologs of *MAK10* or *PET18* in the genome of *M. restricta*, suggesting that a different or modified virus maintenance mechanism may be present in the fungus compared to S. cerevisiae (Table 2). The results also showed that among the *MAK* homologs, *MAK1*, *MAK5*, *MAK11*, *MAK16*, *MAK21*, *MAK7*, and *MAK8* were downregulated by more than 2-fold. In S. cerevisiae, these genes, except for *MAK1*, were involved in 60S ribosomal subunit biosynthesis and M satellite maintenance. Thus, we predicted that MrV40 reduced ribosomal biogenesis within the host. Additionally, *M. restricta* contained a series of *SKI* homologs. However, no *SKI* homologs were differentially expressed in the virus-infected *M. restricta* KCTC 27540 compared to those in the virus-cured strain, indicating that the function of *SKI* genes was not critical for maintaining the virus. In addition to the genes involved in virus maintenance, we observed upregulation of numerous genes required for the tricarboxylic acid (TCA) cycle and the electron transport chain, including MRET_1104 [NADH dehydrogenase (ubiquinone) Fe-S protein 7], MRET_1378 [succinate dehydrogenase (ubiquinone) iron-sulfur subunit], MRET_1953 [NADH dehydrogenase (ubiquinone) Fe-S protein 1], MRET_2042 (fumarate hydratase, class II), MRET_2097 [succinate dehydrogenase (ubiquinone)], MRET_2956 (2-oxoglutarate dehydrogenase E1 component), MRET_3173 (dihydrolipoamide dehydrogenase), and MRET_4117 (aconitate hydratase) (Table 3). These results suggest that virus maintenance and propagation may require higher energy production in the host cell.

**TABLE 2:** Differential expression of genes involved in maintaining dsRNA virus

S. cerevisiae gene	Encoded protein and/or function	Needed by S. cerevisiae virus	*M. restricta* gene ID	Fold change (+virus/−virus)	Reference(s)
*MAK3*	N-acetyltransferase modifying Gag	L-A, M	MRET_0174	0.62	39, 62
*MAK10*	Noncatalytic subunit of N-terminal acetyltransferase	L-A, M	No hits		41, 98
*PET18* (*MAK30*+*MAK31*)	Unknown	L-A, M	No hits		99, 100
*MAK1* (*TOP1*)	DNA topoisomerase I	M	MRET_0706	0.41	101
*MAK2*	60S subunit biosynthesis	M	MRET_4192	0.88	43
*MAK5*	60S subunit biosynthesis	M	MRET_0265	0.44	43
*MAK21*	60S subunit biosynthesis	M	MRET_2922	0.24	43
*MAK11*	60S subunit biosynthesis	M	MRET_2252	0.40	43, 102
*MAK16*	60S subunit biosynthesis	M	MRET_2745	0.48	43, 103
*MAK7* (*RPL4A*)	60S subunit protein L4	M	No hits		43
*MAK8* (*TCM1*)	60S subunit protein L3	M	MRET_1592	0.24	42
*MAK18* (*RPL41B*)	60S subunit protein L41	M	MRET_2856	0.60	104
*SKI1* (*XRN1*)	5′-3′ exonuclease	L-A, M	MRET_4129	0.73	105
*SKI2*	RNA helicase	L-A, M	MRET_2647	0.91	106, 107
*SKI3*	Tetratricopeptide repeat protein	L-A, M	MRET_1481	0.78	106, 107
*SKI4* (*CSL4*)	Exosome noncatalytic core component	L-A, M	MRET_2576	0.96	106, 107
*SKI6*	Exosome noncatalytic core component	L-A, M	MRET_3188	0.55	106, 107
*SKI7*	GTP-binding protein	L-A, M	No hits		106, 107
*SKI8*	WD-repeat protein	L-A, M	No hits		106, 107
*KEX1*	Cell death protease essential for hypochlorite-induced apoptosis	M	MRET_4176	0.93	108–110
*KEX2*	Kexin, a subtilisin-like protease (proprotein convertase)	M	MRET_0618	1.13	109, 110

**TABLE 3:** Differential expression of genes involved in TCA cycle and electron transport chain

Gene	Annotation	Fold change (+virus/−virus)
MRET_1104	NADH dehydrogenase (ubiquinone) Fe-S protein 7	2.85
MRET_1378	Succinate dehydrogenase (ubiquinone) iron-sulfur subunit	3.55
MRET_1709	Cytochrome *c*	2.56
MRET_1953	NADH dehydrogenase (ubiquinone) Fe-S protein 1	2.44
MRET_2042	Fumarate hydratase, class II	2.42
MRET_2097	Succinate dehydrogenase (ubiquinone) flavoprotein subunit	2.19
MRET_2956	2-Oxoglutarate dehydrogenase E1 component	2.21
MRET_3173	Dihydrolipoamide dehydrogenase	2.27
MRET_4117	Aconitate hydratase	3.55

The overall dysregulation of primary metabolism may disturb the normal cell physiology in the *M. restricta* strain infected with MrV40. Indeed, we observed upregulation of genes involved in programmed cell death in the fungal host cells. For example, the expression of MRET_3200 (p38 mitogen-activated kinase [MAP] kinase), MRET_1134 (programmed cell death 6-interacting protein), and MRET_2499 (autophagy-related protein, *ATG101*), which are associated with programmed cell death, was upregulated by 4.43-, 3.14-, and 2.83-fold in the virus-infected fungal cells, respectively. Moreover, MRET_0131 (Fas-associated factor), which is involved in Fas-induced apoptosis, was found to be strongly upregulated (8.80-fold) in the presence of the virus within the fungal host (44). It is well known that programmed cell death is triggered during virus infection (45, 46), and our findings agree with this observation. To confirm that there might be an increase in programmed cell death, we stained cells with annexin V and propidium iodide, which is commonly used to quantitate the apoptosis-like phenomenon (47, 48). Using flow cytometric analysis, we found a higher percentage of positive population in the virus-infected KCTC 27540 strain than in the virus-cured strain, implying that programmed cell death might be increased in the virus-infected *M. restricta* strain (Fig. S4). We also tested whether increased programmed cell death influences other physiological characteristics of the virus-infected cells such as susceptibility to an antifungal drug. However, antifungal susceptibility of virus-infected KCTC 27540 was similar to that of the virus-cured strain (data not shown), suggesting that the viral infection may have a limited effect on the physiological characteristics of the host cells.

10.1128/mBio.01521-20.4 FIG S3 Validation of the differential expression in transcriptome analysis. Differential expression of selected genes was confirmed by Q-RT-PCR. MRET_0131 (Fas-associated factor); MRET_2499 (autophagy-related protein, ATG101); MRET_3200 (p38 MAP kinase); MRET_1953 (NADH dehydrogenase Fe-S protein 1); MRET_2956 (2-oxoglutarated dehydrogenase E1 component); MRET_0230 (large subunit ribosomal protein L8e); MRET_1468 (large subunit ribosomal protein L22e). Download FIG S3, PDF file, 0.2 MB. Copyright © 2020 Park et al. 2020 Park et al. This content is distributed under the terms of the Creative Commons Attribution 4.0 International license.

10.1128/mBio.01521-20.5 FIG S4 Evaluation of apoptosis in *M. restricta* strains. The virus-infected and the virus-cured *M. restricta* KCTC 27540 cells were stained with annexin V and propidium iodide (PI) to evaluate apoptosis. Annexin V binds to phosphatidylserine (PS), which translocates from the inner leaflet of the plasma membrane to the outer leaflet upon apoptosis while the plasma membrane integrity is still maintained (1). PI is permeable when cell membrane was ruptured by damage or upon cell death (2). Overall apoptosis was quantified by annexin V (Ann), and early and late apoptosis was quantified by annexin V (Ann)/PI and by PI, respectively. Statistical analysis for differences between the strains was performed with unpaired *t* tests. *, *P* < 0.01. Download FIG S4, PDF file, 0.10 MB. Copyright © 2020 Park et al. 2020 Park et al. This content is distributed under the terms of the Creative Commons Attribution 4.0 International license.

### MrV40 induced the TLR3-mediated immune system.

Since it was first reported that fungal viral dsRNA induces cytokine production in rabbits (49–51), several studies have demonstrated that TLR3 plays a central role in viral dsRNA recognition and production of inflammatory cytokines in innate immune cells (52, 53). Additionally, a recent study showed that S. cerevisiae viral dsRNA stimulates the immune system through TLR3 in a human embryonic kidney cell line (54). We therefore investigated whether MrV40 itself or the virus-containing *Malassezia* cells alter the expression patterns of TLRs and cytokine production in mammalian cells. To this end, bone marrow-derived dendritic cells (BMDCs) obtained from C57BL/6 mice, which have been used as a model to study the interactions between fungal cells including *Malassezia* and the innate immune system in a mammalian host, were used in our study (55, 56).

**FIG 7:**
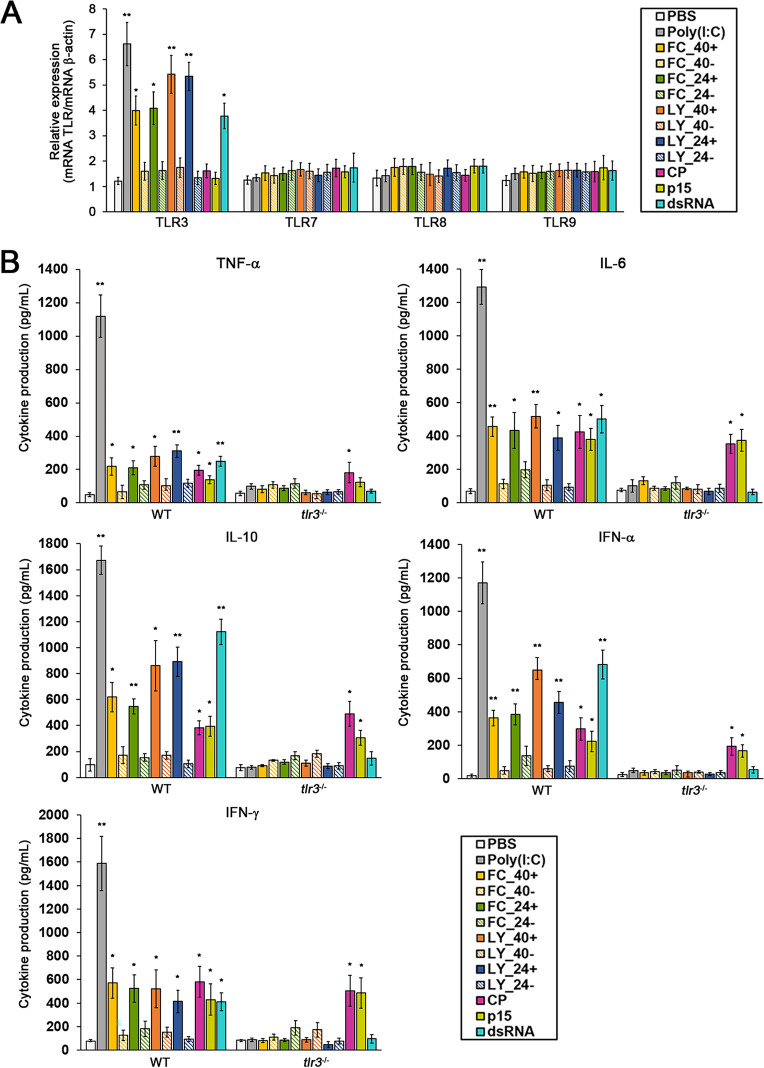
Evaluation of TLR and cytokine levels upon treatment with the viral elements. *TLR* expression (A) and cytokine production (B) in BMDCs coincubated with live fungal cells, total fungal cell lysates, purified MrV40 capsid protein, purified satellite protein, and purified MrV40 dsRNA. PBS and poly(I·C) served as negative and positive controls, respectively. WT, wild-type mice (C57BL/6); *tlr3*^−/−^, TLR3 knockout mice (B6;129S1-Tlr3^tm1Flv^/J, 005217); FC, live fungal cells; LY, cell lysate; 40+, virus-infected KCTC 27540; 40-, virus-cured KCTC 27540; 24+, virus-infected KCTC 27524; 24-, virus-cured KCTC 27524; CP, purified capsid protein; p15, purified MrV40S satellite protein; dsRNA, purified MrV40 dsRNA. Values represent the average from quadruplicates with standard deviations. Statistical analysis for differences between PBS and each sample was performed with unpaired *t* tests. *, *P* < 0.001; **, *P* < 0.0001.

Two independent strain pairs, virus-infected and virus-cured *M. restricta* KCTC 27540 strains as well as virus-infected and virus-cured *M. restricta* KCTC 27524 strains, were used for the study. Live fungal cells, fungal cell lysates, purified capsid protein, purified satellite protein (produced from MrV40S ORF3), and purified MrV40 dsRNA were prepared and coincubated with BMDCs, and the expression of TLRs and cytokines was analyzed. We found that TLR3 expression was significantly upregulated by live fungal cells and fungal cell lysates of the virus-infected *M. restricta* strains, while live cells and their lysates of the virus-cured strains did not enhance the expression of TLR3. Considering that the expression of other TLRs was unchanged upon addition of live cells and cell lysates, we concluded that host response upon treatment with virus-infected *M. restricta* cells was mainly mediated by TLR3 in murine BMDCs. Moreover, upregulation of TLR3 expression was observed in BMDCs treated with purified MrV40 dsRNA but not with purified capsid and satellite proteins, suggesting that MrV40 dsRNA is the causative agent that enhances the expression of TLR3 (Fig. 7A). Moreover, our data suggested that purified capsid and satellite proteins from MrV40 did not influence the expression of TLRs.

The expression of several inflammatory cytokines including tumor necrosis factor alpha (TNF-α), interleukin-6 (IL-6), IL-10, alpha interferon (IFN-α), and IFN-γ, in response to live fungal cells, fungal cell lysates, purified capsid protein, purified satellite protein, and purified MrV40 dsRNA, was evaluated. Particularly, IFN-α and IFN-γ were included because of their involvement in the antiviral response in mammals (57). The results of the cytokine analysis indicated that virus-infected live *M. restricta* cells and their lysates induced the production of TNF-α, IL-6, IL-10, IFN-α, and IFN-γ, while no change in cytokine expression was observed in BMDCs treated with virus-cured live *M. restricta* cells and their cell lysates. Similarly, capsid protein and MrV40 dsRNA also enhanced the expression of all cytokines tested (Fig. 7B). Cytokine profiles were also measured in homozygous TLR3 knockout mice, as our observations suggested a TLR3-mediated host response in BMDCs treated with virus-infected fungal cells. As shown in Fig. 7B, cytokine levels in BMDCs isolated from TLR3 knockout mice were not significantly altered except in those treated with the capsid and satellite proteins. These data suggest that MrV40 increased the production of the cytokines TNF-α, IL-6, IL-10, IFN-α, and IFN-γ in a TLR3-dependent manner in BMDCs. However, as suggested above, the cytokine production induced by the capsid protein seemed to be TLR3 independent. Moreover, the purified satellite protein also caused a TLR3-independent increase in cytokine production except of TNF-α. These results suggest that the capsid and the satellite proteins also contributed to upregulation of cytokine expression in a TLR3-independent manner. Overall, our data suggest that dsRNA in MrV40 triggers an increase in the production of cytokines involved in inflammation and that TLR3 plays a central role in the host response.

## DISCUSSION

In the current study, we detected dsRNA virus in several clinical isolates of *Malassezia* species. Among them, MrV40 identified in *M. restricta* KCTC 27540 was selected, and its genome structure and effects on host gene expression and on the mammalian immune response were evaluated. Our data showed that MrV40 consists of two RNA segments, which we named MrV40L and MrV40S. The results of the genome sequence analysis suggested that these segments were 4,606 and 1,355 bp in length, respectively, and belong to the genus *Totivirus*. Typically, the genomes of the viruses belonging to the genus *Totivirus* consist of nonsegmented dsRNA with sizes between 4.6 and 7.0 kb and contain two ORFs, *gag* and *pol*. Studies have specifically examined the genome structure of *Totivirus* because of the overlapping nature of the two ORFs, where a −1 frameshift occurs, resulting in translation of the fusion protein (2). The overlapping ORFs and frameshift were frequently observed in a compact viral genome to translate proteins and were found in several dsRNA and ssRNA viruses; these ORFs allow ribosomes to translate CP and RDRP continuously with a missing CP termination codon (11, 58, 59). The mechanism of frameshifting in the viral genome is based on the pseudoknot structure of the mRNA for efficient slipping via a slippery site (60). For example, in the genome of the S. cerevisiae L-A virus, the 5′ ORF (*gag*) encodes a 76-kDa CP and the 3′ ORF (*pol*) encodes an RDRP, which is expressed as a 180-kDa CP-RDRP fusion protein generated by a −1 ribosomal frameshift (8, 11). In our study, MrV40L (the major dsRNA segment in MrV40) contained two overlapping ORFs, ORF1 and ORF2. We identified a putative slippery site heptamer, 5′-GGGTTTT-3′, at the region from nt 1968 to 1974 for the −1 ribosomal frameshift, which may be associated with production of the fusion protein of 170 kDa in MrV40L. A previous study suggested that in the S. cerevisiae L-A virus, the rate of ribosomal frameshifts was approximately 1.8%, giving 120 CP and 2 CP-RDRP fusion protein molecules per virus particle (11). Considering the low efficiency of producing the fusion proteins by ribosomal frameshifting, we expected to observe a significantly lower translation rate of the fusion protein; indeed, the putative 170-kDa band was not detected by SDS-PAGE. In addition to MrV40L, we determined the sequence of the satellite dsRNA segment MrV40S and found that it consists of 1,355 nt containing a single ORF, ORF3, producing a novel 15.6-kDa protein. As observed for other totiviruses, the possible toxin-like activity of the protein was investigated in our study, but no growth-inhibitory activity against several bacteria and fungi was observed.

It has been estimated that 30 to 80% of fungal species in nature are infected with viruses, and a fungal host normally shows no specific symptoms upon infection (14). However, several genes were shown to be required for maintaining and propagating viruses in the host fungal cells. In S. cerevisiae, numerous chromosomal genes are known to be involved in viral propagation and expression of the viral killer toxin (36). Furthermore, several genes are known to be responsible for maintaining the L-A virus and M dsRNA in S. cerevisiae. The *MAK* genes are required for the propagation and maintenance of the L-A virus and M dsRNA in S. cerevisiae (61). Among the *MAK* genes, *MAK3*, *MAK10*, and *PET18* are required for the maintenance of both L-A virus and M dsRNA, whereas all other *MAK* genes are responsible only for M dsRNA (38–43). Particularly, *MAK3* encodes an N-acetyltransferase and is required for N-acetylation of the coat protein (38, 62). A previous study showed that the coat proteins without acetylation failed to self-assemble, resulting in the loss of all dsRNA viruses (39). *MAK10* and *PET18* (*MAK30*+*MAK31*) encode a noncatalytic subunit of N-terminal acetyltransferase and a protein of unknown function, respectively. Mutant strains lacking each gene contained unstable viral particles, indicating that the genes are involved in the structural stability of L-A virus and M dsRNA (40). *MAK1* (*MAK17*, *TOP1*) encodes DNA topoisomerase I, and other *MAK* genes including *MAK2*, *MAK5*, *MAK11*, *MAK16*, *MAK21*, *MAK7* (*RPL4A*), *MAK8* (*TCM1*), and *MAK18* (*RPL41B*) are related to 60S ribosomal subunit assembly (43, 63, 64). All mutant strains lacking the above genes showed decreased levels of free 60S ribosomal subunits and the inability to maintain M dsRNA, suggesting that stable propagation of the satellite dsRNA depends on 60S ribosome synthesis (42, 43). In addition to the *MAK* genes, the *SKI* gene family has been shown to be involved in the maintenance and propagation of virus in S. cerevisiae. *SKI1* (*XRN1*) encoding a 5′-to-3′ exonuclease is involved in the degradation of uncapped mRNA including viral mRNA, and *SKI2*, *SKI3*, *SKI6*, *SKI7*, and *SKI8* block the translation of viral mRNAs (65–67).

In the current study, homologs of most *MAK* and *SKI* genes were identified in *M. restricta*. The results of the transcriptome analysis suggested that most *MAK* genes were downregulated, which may in turn reduce ribosome synthesis in the *M. restricta* strain containing MrV40. In contrast, no *SKI* homolog showed significantly altered transcript levels in the *M. restricta* strain harboring MrV40. Moreover, we found enhanced expression of genes involved in energy metabolism and programmed cell death in the *M. restricta* strain containing the virus. S. cerevisiae also displayed relatively small changes in fungal host gene expression upon virus infection, possibly because of coadaptation of the virus within the fungal host (68). Maintenance and propagation of virus within the fungal host may be involved in the posttranscriptional mechanism and may contribute to the minimal changes in host gene expression. Notably, the possibility that an RNA silencing pathway in *M. restricta* cells influences virus maintenance was excluded because of the absence of homologous genes required for the pathway in the genome of the fungus.

In addition to transcriptome analysis, we directly investigated whether the virus influences the cellular morphology of *M. restricta* and the structures of its intracellular organelles by TEM. We observed significantly larger vacuoles in virus-infected *M. restricta* cells. An increased vacuole size upon virus infection has been reported previously. The phytopathogenic fungus Botrytis porri infected with dsRNA virus 1 showed the formation of abundant vacuoles (69), and turnip mosaic virus induced the formation of a large central vacuole in Nicotiana benthamiana plant cells (70). Particularly, in N. benthamiana, turnip mosaic virus particles were shown to accumulate in vacuoles and be protected by the organelle membranes against the harsh host environment, particularly during xylem vessel differentiation. Furthermore, virus accumulation in vacuoles in plant-to-plant virus transmission has been suggested (70). Although the expression of genes involved in vacuole biogenesis was not altered in transcriptome analysis in virus-infected *M. restricta* cells, there may be a relationship between vacuole functions and MrV40 in *M. restricta*. Therefore, additional physiological studies are needed.

TLR3 is well conserved in most vertebrates, localizes on the endosomal transmembrane, and plays a role in immune and nonimmune cells. It has been suggested that TLR3 senses viral dsRNA taken up through endocytosis and contributes to defending the host against viral infection by regulating the expression of a range of cytokines (52, 71). A previous study demonstrated a significant decrease in cytokines (IFN-α/β, IFN-γ, and IL-12p40) and increase in viral PFU in the spleen of *Tlr3*^−/−^ mice infected with mouse cytomegalovirus (72). We found that TLR3 expression was significantly increased by the live cells of two independent virus-infected *M. restricta* strains and their cell lysates, while no change was found by the live cells and cell lysates of the virus-cured strains. The causative agent was very likely to be dsRNA of MrV40 in the virus-infected *M. restricta* strains since purified dsRNA from the MrV40 particles enhanced TLR3 expression. The virus-infected live *M. restricta* cells, their cell lysates, and purified dsRNA from the MrV40 particles also showed increased production of cytokines involved in inflammation. However, in *Tlr3*^−/−^ BMDCs, the production of all cytokines in the cells treated with the virus-infected live *M. restricta* cells, their cell lysates, and purified dsRNA from the MrV40 particles was attenuated, suggesting that TLR3 plays a central role in the host response against dsRNA of MrV40 and mediates the production of the cytokines.

Similarly, the *Leishmania* parasite L. guyanensis infected with the dsRNA virus belonging to *Totivirus* induced a TLR3-mediated inflammatory immune response within the vertebrate host, indicating that the *Leishmania* RNA virus (LRV1) functions as an innate immunogen. Moreover, in *Leishmania*, LRV1 was suggested to stimulate the inflammatory immune response and increase the severity and persistence of the parasite (73). However, whether MrV40 contributes to hyper- or hypopathogenicity of *M. restricta* remains unclear. Therefore, further studies to characterize the potential function of MrV40 as an innate immunogen are needed. In addition, since our data suggest contribution of the MrV40 capsid and the satellite proteins to host cytokine expression in a TLR3-independent manner, additional studies are needed to define a role of the capsid and the satellite proteins in interactions between these viral components and the mammalian host cells.

Overall, our study demonstrated the existence of a dsRNA virus within *M. restricta*. We also determined the sequences and structure of the viral genome along with the independent RNA segment. Moreover, we identified viruses not only from different strains of *M. restricta* but also from other *Malassezia* species, although variations were observed in the viral genomes. Evidence that the viral nucleic acid from MrV40 induces a TLR3-mediated inflammatory immune response was obtained, suggesting that a viral element contributes to the pathogenicity of *Malassezia*.

## MATERIALS AND METHODS

### Strains and growth conditions.

*M. restricta* KCTC 27512, KCTC 27516, KCTC 27518, KCTC 27524, KCTC 27540, KCTC 27543, KCTC 27550, KCTC 27879, KCTC 27880, KCTC 27882, and KCTC 27883, *M. globosa* CBS 7966, *M. pachydermatis* KCTC 27587, and *M. sympodialis* KCTC 27817 were obtained as previously described and cultured on Leeming and Notman agar (LNA) medium at 34°C for 3 days (29, 74–76). Among these strains, *M. restricta* KCTC 27540 was used to identify dsRNA viruses. E. coli DH10B and BL21 were grown in Luria-Bertani broth at 37°C.

### Purification of virus particles.

The virus particles were purified as previously described with some modifications (69). Briefly, approximately 10 g of *M. restricta* cells was harvested and suspended in 10 ml of extraction buffer (0.1 M sodium phosphate buffer containing 3% [vol/vol] Triton X-100, pH 7.0). The suspended cells were vortexed with glass beads and centrifuged at 10,000 × *g* at 4°C for 20 min to remove cell debris. The supernatant containing crude extracts was ultracentrifuged at 119,000 × *g* at 4°C for 2 h to precipitate all particles. The resulting pellet was suspended in 1 ml of 0.1 M sodium phosphate buffer (pH 7.0), and the suspension was centrifuged at 16,000 × *g* at 4°C for 30 min to remove large particles. The supernatant was overlaid on sucrose solution with a gradient concentration ranging from 10% to 40% (wt/vol) and centrifuged at 70,000 × *g* at 4°C for 2 h. Fractions (700 μl/each fraction) were collected from the top of the ultracentrifuged sucrose gradient solution. Each fraction was analyzed by 1% agarose gel electrophoresis and 8% SDS-PAGE to detect dsRNA segments and protein, respectively. Methods for cell fixation and TEM are described in Text S1 in the supplemental material.

### Determination of MrV40L and MrV40S sequences.

The reference genome sequence was required to obtain the complete sequence of MrV40L by Illumina MiSeq. We first obtained the partial fragment of MrV40L and determined its nucleotide sequence. Briefly, 1 μg of cDNA obtained as described above was amplified using 1 μM tagged oligo_1 primer to bind the tag and 1.25 units of *Taq* DNA polymerase (Bioneer, Daejeon, South Korea). The amplicons were cloned into the pGEM-T vector (Promega, Madison, WI, USA), transformed into E. coli DH10B, and sequenced with the universal M13 primers. The resulting partial sequences of MrV40L showed high similarity with the genome of *Scheffersomyces segobiensis* virus L isolate NRRL Y-11571, which was used to assemble the reference genome in our study. To sequence the entire genome of the virus from *M. restricta* KCTC 27540, dsRNA was purified using cellulose A (Advantec, Taipei, Taiwan) from total cell extract as described previously (77). After purification, purified dsRNA was treated with Ambion DNase I (Thermo Fisher Scientific, Waltham, MA, USA) to remove remaining genomic DNA. The sequencing library was constructed using the TruSeq stranded total RNA sample prep kit (Illumina, San Diego, CA, USA) following the manufacturer’s instructions and excluding poly(A) selection. The resulting library was sequenced on an Illumina MiSeq instrument according to the manufacturer’s instructions. The generated raw sequences, 250-bp paired-end reads, were assembled by CLC Genomics Workbench v7.5 (Qiagen, Hilden, Germany), and the resulting contigs were identified by BLASTN search of the NCBI nucleotide database. Based on the reference genome, two contigs of MrV40L were assembled. The gap between the contigs was sequenced by gap-filling RT-PCR using primers Linkage1_V40 and Linkage2_V40) (see Table S2), and both termini were sequenced by RACE PCR using a 5′/3′ RACE kit, second generation (Roche, Basel, Switzerland), according to the 5′ RACE protocol described by the manufacturer.

10.1128/mBio.01521-20.8 TABLE S2 Primers used for sequencing and heterologous expression of viral proteins. Download Table S2, PDF file, 0.02 MB. Copyright © 2020 Park et al. 2020 Park et al. This content is distributed under the terms of the Creative Commons Attribution 4.0 International license.

To determine the MrV40S genome, the RACE PCR method was used because we failed to find any contig of MrV40S. Like MrV40L, the partial sequence of cDNA from MrV40S was amplified using the tagged oligo_1 primer. The amplicons were cloned into the pGEM-T vector (Promega) and sequenced. Using the partial sequence obtained, 5′ RACE PCR was performed and repeated until no additional sequence was found.

### *In silico* analysis.

To identify the ORFs in MrV40L and MrV40S, the nucleotide sequences of the genomes were analyzed by ORFfinder (https://www.ncbi.nlm.nih.gov/orffinder/). To predict ribosomal frameshift signals, a putative slippery site was identified using FSFinder2 (78). The amino acid sequences of CP and RDRP of MrV40L were analyzed using the Pfam database (http://pfam.xfam.org/) and BLASTP (https://blast.ncbi.nlm.nih.gov/). For sequence alignment, Jalview2.0 was used (79). The phylogenetic tree showing the relationship of MrV40L with RDRP of other dsRNA fungal viruses was constructed using MEGA X, and phylogenetic reconstruction analysis was performed by the neighbor-joining method with 1,000 bootstrap replications (80, 81).

### Mice and cell culture.

Wild-type C57BL/6 mice were purchased from Orient Bio Co. (Gyeonggi-do, South Korea). *TLR3*^−/−^ (B6;129S1-Tlr3^tm1Flv^/J, 005217) mice were obtained from Jackson Laboratory (Bar Harbor, ME, USA). All animal experimental procedures were reviewed and approved by the Institutional Animal Care and Use Committee of Hanyang University (protocol 2018-0085) and performed in accordance with Korean Food and Drug Administration guidelines. All animals were maintained in a specific-pathogen-free environment. Primary BMDCs were isolated from C57BL/6 mice and cultured in Dulbecco’s modified Eagle’s medium for 3 to 5 days in the presence of 20 ng/ml recombinant granulocyte-macrophage colony-stimulating factor (R&D Systems, Minneapolis, MN, USA) as described previously (56). Cell cultures were stained to detect dendritic cells with CD11c-fluorescein isothiocyanate (FITC) (eBiosciences, San Diego, CA, USA).

### Cytokine measurement.

Mouse cytokines in the culture supernatants were measured with a BD OptEIA enzyme-linked immunosorbent assay (ELISA) kit (BD Biosciences, Franklin Lakes, NJ, USA) as described previously (82). All assays were performed according to the manufacturer’s instructions. Two independent strain pairs, virus-infected and virus-cured *M. restricta* KCTC 27540 and virus-infected and virus-cured *M. restricta* KCTC 27540, were used. To treat BMDCs with live fungal cells, a 20:1 ratio (2.0 × 10^6^ and 1.0 × 10^5^ cells/ml of *M. restricta* and BMDCs, respectively) was used. A final concentration of 100 μg/ml (total protein/volume) of fungal cell lysates was used to treat BMDCs. A final concentration of 1 μM purified MrV40 capsid and satellite proteins and 500 ng/ml of purified MrV40 dsRNA from virus-infected *M. restricta* KCTC 27540 strain were incubated with BMDCs. Phosphate-buffered saline (PBS) and poly(I)-poly(C) [poly(I·C), 25 μg/ml] served as negative and positive controls, respectively. Cytokines were measured 6 h after treatment. All experiments were repeated four times.

### Other methods.

For descriptions of cDNA synthesis, RNA isolation, transcriptome analysis by RNA sequencing, quantitative real-time PCR, heterologous expression and purification of MrV40 proteins, cell fixation and transmission electron microscopy, and flow cytometric analysis, see Text S1 in the supplemental material.

### Data availability.

The complete genome sequences of MrV40L and MrV40S were deposited into GenBank under accession numbers MN603497 and MN603498, respectively. The transcriptome data were deposited in Gene Expression Omnibus under the accession number GSE138985.

10.1128/mBio.01521-20.6 FIG S5 Purification of the MrV40 capsid protein. The capsid protein encoded by ORF1 of MrV40 was heterologously expressed in E. coli, purified using a His tag column, and used to analyze the expression of TLRs and cytokines in BMDCs. Download FIG S5, PDF file, 0.02 MB. Copyright © 2020 Park et al. 2020 Park et al. This content is distributed under the terms of the Creative Commons Attribution 4.0 International license.

10.1128/mBio.01521-20.9 TABLE S3 Primers used for Q-RT-PCR. Download Table S3, PDF file, 0.09 MB. Copyright © 2020 Park et al. 2020 Park et al. This content is distributed under the terms of the Creative Commons Attribution 4.0 International license.
