# Supplementary material for: A Novel Virus Alters Gene Expression and Vacuolar Morphology in Malassezia Cells and Induces a TLR3-Mediated Inflammatory Immune Response
Source: mBio. 2020 Sep 1;11(5):e01521-20. doi: 10.1128/mBio.01521-20 (PMC7468201; doi:10.1128/mBio.01521-20)
Supplement: FIG S1 [file mBio.01521-20-sf001.pdf]

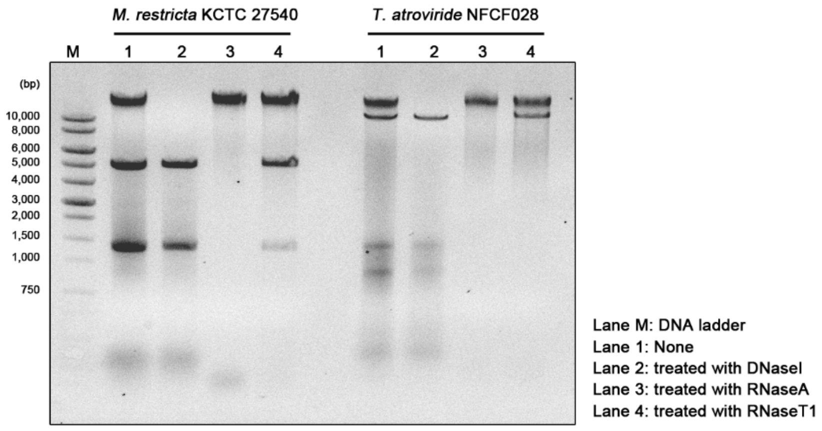

Fig. S1. Extrachromosomal dsRNA segments in *Malassezia restricta* KCTC 27540 and *T. atroviride* NFCF028. Nucleic acids from the strains were separated on a 0.7% agarose gel. Lane 1, total nucleic acids; lane 2, total nucleic acids treated with DNase I; lane 3, total nucleic acids treated with RNase A; lane 4, total nucleic acids treated with RNase T1.
