# Supplementary material for: A Novel Virus Alters Gene Expression and Vacuolar Morphology in Malassezia Cells and Induces a TLR3-Mediated Inflammatory Immune Response
Source: mBio. 2020 Sep 1;11(5):e01521-20. doi: 10.1128/mBio.01521-20 (PMC7468201; doi:10.1128/mBio.01521-20)
Supplement: FIG S2 [file mBio.01521-20-sf002.pdf]

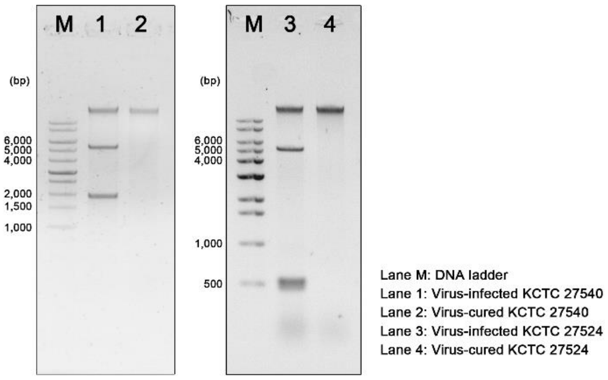

Fig. S2. Confirmation of the virus-cured *Malassezia restricta* strain. Total nucleic acids from the virus-infected and the virus-cured *M. restricta* KCTC 27540 and KCTC 27524 strains were extracted and treated with RNase T1. The dsRNA viral segments were not detected in the virus-cured *M. restricta* KCTC 27540 and KCTC 27524 strains (lane 2 and lane 4).
