# Supplementary material for: A Novel Virus Alters Gene Expression and Vacuolar Morphology in Malassezia Cells and Induces a TLR3-Mediated Inflammatory Immune Response
Source: mBio. 2020 Sep 1;11(5):e01521-20. doi: 10.1128/mBio.01521-20 (PMC7468201; doi:10.1128/mBio.01521-20)
Supplement: TABLE S1 [file mBio.01521-20-st001.pdf]

Table S1. Expression of genes calculated from RNA-seq data

Gene ID	Annotation	Reads (TPM)	
		+Virus	-Virus
MRET_0001	uncharacterized protein	0	0
MRET_0002	iron transport multicopper oxidase	0	0
MRET_0003	carboxypeptidase D	41.14	75.11
MRET_0004	Csr1-phosphatidylinositol transfer protein	640.09	634.52
MRET_0005	Bromodomain associated protein	25.39	48.84
MRET_0006	ATP-dependent RNA helicase SUPV3L1/SUV3	31.01	52.81
MRET_0007	diphthine-ammonia ligase	8.97	16.23
MRET_0008	oligosaccharyltransferase complex subunit beta	42.48	77.19
MRET_0009	pheromone-dependent cell cycle arrest protein Far11	27.31	59.25
MRET_0010	mitochondrial distribution and morphology protein 10	53.53	60.18
MRET_0011	pre-mRNA-processing factor 8	317.53	248.21
MRET_0012	DUF410 domain protein	116.97	363.53
MRET_0013	acetyltransferase (GNAT) family	886.71	746.57
MRET_0014	small nuclear ribonucleoprotein G	136.09	136.61
MRET_0015	metal homeostatis protein BSD2	97.2	363.97
MRET_0016	uncharacterized protein	205.34	226.76
MRET_0017	DnaJ domain protein	595.35	439.68
MRET_0018	6-phosphofructokinase 1	107.33	105.28
MRET_0019	lipase	3678.04	10249.18
MRET_0020	uncharacterized protein	73.69	139.31
MRET_0021	cytoplasmic GTPase-activating protein	18.59	58.32
MRET_0022	uncharacterized protein	15.86	36.13
MRET_0023	U4/U6 small nuclear ribonucleoprotein SNU13	154.25	540.65
MRET_0024	DnaJ homolog subfamily B member 4	1343.75	1798.91
MRET_0025	AAA family ATPase	47.17	88.2
MRET_0026	PHD finger domain protein	10.69	48.08
MRET_0027	pre-mRNA-splicing factor ATP-dependent RNA helicase DHX16	12.58	17.89
MRET_0028	RING-H2 domain core subunit of multiple ubiquitin ligase complexes	1039.03	886.43
MRET_0029	elongator complex protein 2	51.24	54.29
MRET_0030	leucine-rich repeat protein	22.33	30.33
MRET_0031	helicase associated domain (HA2) containing protein	30.68	70.09
MRET_0032	kinetochore protein Mis13/DSN1	15.49	41.56
MRET_0033	THO complex subunit 3	365.31	429.76
MRET_0034	microtubule-associated protein, RP/EB family	39.38	163.24

MRET_0035	cysteine desulfurase	1059.79	1001.13
MRET_0036	optic atrophy 3 protein (OPA3)	107.16	253.76
MRET_0037	polyadenylate-binding protein 2	139.53	357.88
MRET_0038	26 proteasome complex subunit DSS1	235.2	452.65
MRET_0039	oligosaccharyltransferase complex subunit alpha (ribophorin I)	77.09	74.43
MRET_0040	Tic20-like protein	42.86	60.56
MRET_0041	rRNA-processing protein CGR1	82.16	248.54
MRET_0042	NADH dehydrogenase (ubiquinone) Fe-S protein 6	226.47	534.7
MRET_0043	pheromone-regulated membrane protein	318.41	171.24
MRET_0044	transcription initiation factor TFIIH subunit 2	90.72	138.64
MRET_0045	protein transport protein SEC31	375.03	165.41
MRET_0046	vacuolar protein sorting-associated protein 18	58.11	50.48
MRET_0047	triose/dihydroxyacetone kinase/FAD-AMP lyase (cyclizing)	1609.71	1305.02
MRET_0048	mitochondrial DNA replication protein	54.54	99.03
MRET_0049	ESCRT-II complex subunit VPS22	132.98	218.96
MRET_0050	homeobox domain protein	249.46	400.45
MRET_0051	ubiquinone biosynthesis monooxygenase Coq7	434.37	294.57
MRET_0052	uncharacterized protein	78.9	127.91
MRET_0053	2-(3-amino-3-carboxypropyl)histidine synthase	127.44	124.66
MRET_0054	pyruvate dehydrogenase protein X component, mitochondrial precursor	609.73	442.64
MRET_0055	palmitoyltransferase ZDHHC13/17	107.28	68.46
MRET_0056	uncharacterized protein	24.84	48.55
MRET_0057	large subunit ribosomal protein L34	148.95	126.91
MRET_0058	DNA-binding protein HGH1	40.62	65.52
MRET_0059	NTF2-related export protein 1/2	467.57	417.02
MRET_0060	uncharacterized protein	325.15	197.08
MRET_0061	solute carrier family 36 (proton-coupled amino acid transporter)	57.58	114.74
MRET_0062	C-4 methylsterol oxidase	564.39	498.99
MRET_0063	TBC1 domain family member 8/9	346.67	205.3
MRET_0064	uncharacterized protein	56.26	49.76
MRET_0065	transcription elongation factor	39.11	66.91
MRET_0066	SCY1-like protein 2	47.52	104.7
MRET_0067	protein YIH1	102.06	210.81
MRET_0068	dehydrogenase	168.04	375.42
MRET_0069	dehydrogenase	62.07	119.21
MRET_0070	golgi reassembly stacking protein	63.84	132.79
MRET_0071	glycoside hydrolase family 5 protein	20.64	45.03

MRET_0072	protein of unknown function (DUF1769)	93.34	141.99
MRET_0073	Bsp1 protein	17.88	60.7
MRET_0074	uncharacterized protein	31.79	152.64
MRET_0075	protein of unknown function (DUF2422)	21.48	90.94
MRET_0076	uncharacterized protein	108.32	93.63
MRET_0077	4-coumarate-coA ligase	201.03	163.55
MRET_0078	pyridoxamine 5'-phosphate oxidase	308.01	345.85
MRET_0079	enhancer of yellow 2 transcription factor	408.33	463.06
MRET_0080	LCCL domain protein	16	26.94
MRET_0081	syntaxin-binding protein 5	76.69	53.03
MRET_0082	C2 domain protein	247.29	88.97
MRET_0083	fungal Zn(2)-Cys(6) binuclear cluster domain protein	274.99	269.52
MRET_0084	ataxin-3	1148.37	829.45
MRET_0085	RuvB-like protein 2	78.2	188.97
MRET_0086	uncharacterized protein	3.92	11
MRET_0087	glutathione synthase	33.82	33.08
MRET_0088	sphingolipid 4-desaturase/C4-monooxygenase	90.22	58.85
MRET_0089	uncharacterized protein	16.19	46.05
MRET_0090	NADH dehydrogenase (ubiquinone) flavoprotein 1	162.13	141.94
MRET_0091	SURF1-like protein	22.26	41.84
MRET_0092	exocyst complex component 2	21.69	36.03
MRET_0093	26S proteasome regulatory subunit N5	71.94	112.16
MRET_0094	adenosine kinase	181.06	183.68
MRET_0095	transporter	33.17	20.91
MRET_0096	uncharacterized protein	128.65	95.02
MRET_0097	transcriptional regulation protein GF11	25.84	106.14
MRET_0098	aarF domain kinase	18.97	45.26
MRET_0099	nucleoside-diphosphate kinase	473.29	784.92
MRET_0100	uncharacterized protein	35.77	65.25
MRET_0101	uncharacterized protein	44.41	103.1
MRET_0102	glutathione peroxidase	15418.96	13664.52
MRET_0103	ornithine carbamoyltransferase	101.7	153.1
MRET_0104	small EDRK-rich factor	244.58	241.61
MRET_0105	uncharacterized protein	50.15	64.47
MRET_0106	protein FRG1	334.53	161.8
MRET_0107	6-phosphofructo-2-kinase/fructose-2,6-biphosphatase 4	276.53	142.81
MRET_0108	uncharacterized protein	78.53	109.79

MRET_0109	uncharacterized protein	45.12	84.15
MRET_0110	DNA repair protein	431.89	327.87
MRET_0111	2-dehydropantoate 2-reductase	44.28	45.64
MRET_0112	subunit of the DSC ubiquitin ligase complex	40.33	54.21
MRET_0113	transcription initiation factor TFIID subunit 1	26.29	49.52
MRET_0114	TatD DNase family protein	25.73	42.17
MRET_0115	uncharacterized protein	118.34	832.44
MRET_0116	COP9 signalosome complex subunit 2	84.92	216.53
MRET_0117	mitochondrial organizing structure protein 2	211.68	332.09
MRET_0118	coiled-coil domain protein 130	37.43	95.66
MRET_0119	protein farnesyltransferase/geranylgeranyltransferase type-1 subunit alpha	34.57	96.13
MRET_0120	uncharacterized protein	568.87	1244.42
MRET_0121	uncharacterized protein	658.24	1145.85
MRET_0122	uncharacterized protein	709.81	1125.89
MRET_0123	cystathionine	851.59	577.53
MRET_0124	membrane-associated progesterone receptor component	1072.3	1128.67
MRET_0125	DUF1992 domain protein	96.86	368.42
MRET_0126	SMR domain protein	291.66	365.67
MRET_0127	ATP-dependent helicase IRC3	172.54	149.91
MRET_0128	MEMO1 family protein	273.51	128.45
MRET_0129	uncharacterized protein	105.51	100.82
MRET_0130	uncharacterized protein	101.88	97.34
MRET_0131	FAS-associated factor 2	494.03	56.17
MRET_0132	cortical ER protein involved in ER-plasma membrane tethering	34.04	21.15
MRET_0133	Na+/H+ antiporter	80.67	13.62
MRET_0134	methyltransferase	104.93	290.32
MRET_0135	alpha/beta-hydrolase	42.24	45.9
MRET_0136	pseudouridylate synthase/pseudouridine kinase	75.49	63.44
MRET_0137	sulfhydryl oxidase	329.29	137.55
MRET_0138	uncharacterized protein	1450.01	419.49
MRET_0139	uncharacterized protein	71.52	145.21
MRET_0140	protein-L-isoaspartate(D-aspartate) O-methyltransferase	190.61	117.54
MRET_0141	protein OS-9	519.38	253.6
MRET_0142	DUF1183 domain protein	222.37	212.53
MRET_0143	peptide-methionine (R)-S-oxide reductase	83.73	243.16
MRET_0144	uncharacterized protein	776.79	2537.67
MRET_0145	adenylosuccinate lyase	89.98	109.12

MRET_0146	DNA cross-link repair 1A protein	114.3	42.18
MRET_0147	cyclin-C	36.27	57.28
MRET_0148	uncharacterized protein	70.01	62.48
MRET_0149	uncharacterized protein	131.96	78.86
MRET_0150	uncharacterized protein	23.36	18.89
MRET_0151	2,5-diamino-6-(ribosylamino)-4(3H)-pyrimidinone 5'-phosphate reductase	57.92	38.22
MRET_0152	transcription factor C subunit 7	119.64	116.16
MRET_0153	HAP4 transcription factor	140.65	136.3
MRET_0154	nicotinamide N-methyltransferase	71.69	121.26
MRET_0155	poly(A) RNA-binding protein	181.94	147.34
MRET_0156	small subunit ribosomal protein S5	164.67	219.46
MRET_0157	exocyst complex component 4	44.15	45.67
MRET_0158	protein N-lysine methyltransferase METTL21D	55.66	43.2
MRET_0159	protein MPE1	114.16	114.51
MRET_0160	vacuolar protein sorting-associated protein VTA1	42.13	63.85
MRET_0161	succinyl-CoA synthetase alpha subunit	643.8	775.7
MRET_0162	deoxyhypusine monooxygenase	236.33	195.07
MRET_0163	mRNA turnover protein 4	55.44	80.84
MRET_0164	mitochondrial import inner membrane translocase subunit TIM21	87.43	134.29
MRET_0165	uncharacterized protein	3896.55	2379.36
MRET_0166	ubiquitin carboxyl-terminal hydrolase 12/46	21.1	39.57
MRET_0167	meiosis induction protein kinase IME2/SME1	10.15	40.64
MRET_0168	centractin	54.32	119.42
MRET_0169	alpha-1,3-glucosyltransferase	25.04	43.09
MRET_0170	translocation protein SEC62	48.22	102.08
MRET_0171	condensin complex subunit 1	31.69	46.45
MRET_0172	cwfJ domain protein	188.93	82.23
MRET_0173	vacuolar ATPase assembly integral membrane protein VMA21	131.9	246.11
MRET_0174	N-alpha-acetyltransferase 30	40.03	65.02
MRET_0175	alpha/beta-hydrolase	390.61	285.57
MRET_0176	alpha/beta-hydrolase	46.19	95.91
MRET_0177	alpha-1,2-glucosyltransferase	57.46	119.64
MRET_0178	tyrosine-protein kinase srms	67.18	94.95
MRET_0179	uncharacterized protein	11.1	28.1
MRET_0180	mannose-6-phosphate isomerase	29.93	67.69
MRET_0181	5'-3' exoribonuclease 2	39.35	80.67
MRET_0182	PITH domain protein	246.56	247.53

MRET_0183	NADH dehydrogenase (ubiquinone) 1 beta subcomplex subunit 7	104.17	240.07
MRET_0184	small subunit ribosomal protein YMR-31	100.74	191.54
MRET_0185	syntaxin 16	53.74	47.9
MRET_0186	protein NAR1	246.34	133.39
MRET_0187	U3 small nucleolar ribonucleoprotein protein IMP3	24.2	32.8
MRET_0188	intermediate cleaving peptidase 55	22.01	61.97
MRET_0189	kinesin family member 20	12.19	39.33
MRET_0190	RNA recognition motif domain protein	57.84	71.23
MRET_0191	pentatricopeptide repeat protein	20.94	33.39
MRET_0192	alpha 1,2-mannosyltransferase	86.32	144.83
MRET_0193	transcription initiation factor TFIID component TAF4 family	34.59	68.37
MRET_0194	inosine-5'-monophosphate dehydrogenase	58.74	221.7
MRET_0195	RuvB-like protein 1 (pontin 52)	80.91	120.08
MRET_0196	uroporphyrinogen decarboxylase	215.18	271.28
MRET_0197	protein of unknown function (DUF2456)	53.96	81.29
MRET_0198	uncharacterized protein	30.36	96.54
MRET_0199	DNA-directed RNA polymerase II subunit RPB3	39.62	129.21
MRET_0200	alpha 1,2-mannosyltransferase	16.41	59.86
MRET_0201	alpha 1,2-mannosyltransferase	69.54	110.08
MRET_0202	component of the NuA4 histone acetyltransferase complex	42.51	57.5
MRET_0203	nuclear cap-binding protein subunit 1	54.22	52.83
MRET_0204	transcription elongation factor SPT4	43.77	103.99
MRET_0205	type II pantothenate kinase	24.1	79.8
MRET_0206	translocation protein SEC63	123.3	160.65
MRET_0207	tRNA (guanine-N(7)-)-methyltransferase subunit TRM82	328.55	232.84
MRET_0208	elongation factor 3	33.43	53.32
MRET_0209	peroxin-3	41.88	39.71
MRET_0210	acyl CoA binding protein	72.49	104.85
MRET_0211	5-formyltetrahydrofolate cyclo-ligase	40.57	60.47
MRET_0212	dolichyl-phosphate-mannose-protein mannosyltransferase	96.83	94.64
MRET_0213	solute carrier family 25 (mitochondrial folate transporter), member 32	150.39	94.78
MRET_0214	polynucleotide 5'-hydroxyl-kinase GRC3/NOL9	30.1	30.64
MRET_0215	transcription factor	72.58	68.4
MRET_0216	rRNA small subunit pseudouridine methyltransferase Nep1	201.79	221.08
MRET_0217	cell division cycle 14	10.13	39.83
MRET_0218	uncharacterized protein	168	129.74
MRET_0219	protein CMC4	345.19	279.84

MRET_0220	folylpolyglutamate synthase	23.8	18.1
MRET_0221	telomere length regulation protein	19.54	26.74
MRET_0222	transcription factor	77.04	85.69
MRET_0223	variant SH3 domain protein	159.24	158.61
MRET_0224	cytochrome c oxidase subunit 15	156.99	241.54
MRET_0225	vacuolar transporter chaperone	92.76	143.1
MRET_0226	Lhp1-RNA binding protein	31.23	84.36
MRET_0227	ATP-dependent RNA helicase DDX31/DBP7	10.77	20.27
MRET_0228	protein SDA1	28.44	55.48
MRET_0229	predicted membrane protein required for lumenal ER protein retention	8.76	14.63
MRET_0230	large subunit ribosomal protein L8e	221.35	1382.14
MRET_0231	A1 cistron-splicing factor AAR2	97.63	125.91
MRET_0232	translation initiation factor IF-3	86.98	81.22
MRET_0233	golgi-specific brefeldin A-resistance guanine nucleotide exchange factor 1	105.75	51.91
MRET_0234	Ras-related protein Rab-1A	584.62	536.99
MRET_0235	solute carrier family 25 (peroxisomal adenine nucleotide transporter), member 17	90.87	77.21
MRET_0236	transcriptional repressor NF-X1	67.42	54.82
MRET_0237	uncharacterized protein	66.67	49.21
MRET_0238	DUF250 domain membrane protein	183.36	178.46
MRET_0239	DNA damage-responsive transcriptional repressor	78.51	55.21
MRET_0240	uncharacterized protein	23.98	38.5
MRET_0241	seryl-tRNA synthetase	89.64	50.06
MRET_0242	dCMP deaminase	20.16	21.7
MRET_0243	solute carrier family 31 (copper transporter), member 1	20.73	33.22
MRET_0244	UDP-glucose--hexose-1-phosphate uridylyltransferase	187.72	81.44
MRET_0245	uncharacterized protein	122.07	92.11
MRET_0246	NET1-associated nuclear protein 1 (U3 small nucleolar RNA-associated protein 17)	112.8	53.13
MRET_0247	RNA polymerase II subunit A C-terminal domain phosphatase	50.91	78.75
MRET_0248	anaphase-promoting complex subunit 10	39.21	127.39
MRET_0249	DNA replication and checkpoint protein	30.32	72.27
MRET_0250	DNA repair protein RAD5	356.76	380.79
MRET_0251	alpha-1,6-mannosyltransferase	134	162.98
MRET_0252	very-long-chain (3R)-3-hydroxyacyl-CoA dehydratase	391.4	220.86
MRET_0253	alkyl hydroperoxide reductase Thiol specific antioxidant Mal allergen	269.29	385.09
MRET_0254	antiviral helicase SLH1	41.8	37.06
MRET_0255	glutaminyl-peptide cyclotransferase	163.92	106.47
MRET_0256	nitrogen permease regulator 2-like protein	79.58	61.25

MRET_0257	mitochondrial inner membrane carnitine transporter	33.8	71.24
MRET_0258	CDP-diacylglycerol--inositol 3-phosphatidyltransferase	76.51	206.65
MRET_0259	origin recognition complex subunit 3	31.76	35.02
MRET_0260	ATP-dependent RNA helicase MSS116, mitochondrial	97.03	77.54
MRET_0261	uncharacterized protein	4520.94	4076.95
MRET_0262	phosphodiesterase	117.19	255.28
MRET_0263	nicotinamide mononucleotide adenylyltransferase	210.09	428.5
MRET_0264	homeobox transcription factor	24.41	47.31
MRET_0265	ATP-dependent RNA helicase DDX24/MAK5	16.76	38.38
MRET_0266	U3 small nucleolar RNA-associated protein 4	14.97	31.39
MRET_0267	glutamate 5-kinase	44.79	82.16
MRET_0268	small subunit ribosomal protein S6	72.56	137.48
MRET_0269	replication factor C subunit 2/4	77.41	146.39
MRET_0270	chromatin assembly factor 1 subunit A	110.17	52.18
MRET_0271	kinetochore protein Spc24, fungi type	29.12	67.66
MRET_0272	C-8 sterol isomerase	41.53	86.73
MRET_0273	MFS family protein	98.83	270.16
MRET_0274	E3 ubiquitin-protein ligase RAD18	80.3	100.32
MRET_0275	zinc finger protein, C2H2 type	27.65	51.16
MRET_0276	RNA recognition motif domain protein	12.84	29.39
MRET_0277	protein JSN1	259.3	141.63
MRET_0278	uncharacterized protein	670.87	595.85
MRET_0279	M-phase inducer tyrosine phosphatase	14.98	31.38
MRET_0280	DNA polymerase phi	12.68	41.36
MRET_0281	mitochondrial import inner membrane translocase subunit TIM8	82.22	211.29
MRET_0282	casein kinase I	88.5	177.3
MRET_0283	monooxygenase	66.32	78.8
MRET_0284	mitogen-activated protein kinase organizer 1	59.22	44.07
MRET_0285	metallo-beta-lactamase superfamily protein	79.04	77.83
MRET_0286	Ran GTPase-activating protein 1	66.71	112.12
MRET_0287	myotubularin-related protein 6/7/8	38.44	101.93
MRET_0288	elongator complex protein 3	35.92	50.5
MRET_0289	fatty acid synthetase	120.84	110.5
MRET_0290	glycolipid transfer protein HET-C2	277.4	1430.08
MRET_0291	fructose-bisphosphate aldolase, class II	1343.96	1207.62
MRET_0292	Ca2+ transporting ATPase, sarcoplasmic/endoplasmic reticulum	74.34	72
MRET_0293	uncharacterized protein	116.92	115.85

MRET_0294	uncharacterized protein	10.81	25.5
MRET_0295	cytochrome c peroxidase	1230.63	504.72
MRET_0296	cytoplasmic inorganic pyrophosphatase (PPase)	198.29	246.72
MRET_0297	pyruvate dehydrogenase E1 component beta subunit	407.56	407.37
MRET_0298	casein kinase II subunit beta	114.27	193.5
MRET_0299	translocation protein SEC66	45.28	81.51
MRET_0300	serine/threonine-protein phosphatase 2A regulatory subunit B'	116.37	246.34
MRET_0301	prefoldin subunit 1	33.49	101.53
MRET_0302	cleavage stimulation factor subunit 2	41.93	75.91
MRET_0303	dTMP kinase	29.02	36.86
MRET_0304	U1 snRNP splicing complex subunit Luc7	92.55	203.12
MRET_0305	uncharacterized protein	52.55	103.41
MRET_0306	inositol polyphosphate phosphatase	763.58	344.21
MRET_0307	protein N-lysine methyltransferase METTL21A	50.91	29.02
MRET_0308	cysteinyl-tRNA synthetase	122.98	77.74
MRET_0309	vacuolar protein sorting-associated protein 33	39.1	54.74
MRET_0310	component of cytosolic iron-sulfur protein assembly	347.5	278.59
MRET_0311	transcriptional enhancer factor	37.13	19.04
MRET_0312	ribonucleases P/MRP protein subunit RPP40	101.99	59.34
MRET_0313	sphingolipid long chain base-responsive protein	53.76	49.2
MRET_0314	precorrin-2 dehydrogenase/sirohydrochlorin ferrochelatase	229.06	165.49
MRET_0315	telomere length regulation protein	75.93	38.23
MRET_0316	transcriptional regulator CBF1	186.87	567.35
MRET_0317	translation initiation factor 5A	256.57	641.85
MRET_0318	chitin synthase	23.48	25.12
MRET_0319	mitogen-activated protein kinase 1/3	423.26	164.91
MRET_0320	chitin synthase	280.44	126.31
MRET_0321	zinc finger protein	220.47	184.15
MRET_0322	serine/threonine-protein kinase receptor-associated protein	89.59	163.93
MRET_0323	cell cycle checkpoint protein	88.1	154.38
MRET_0324	SNARE domain protein	199.5	118.97
MRET_0325	DNA repair protein RAD51	1418.28	1095.21
MRET_0326	amino acid transporter	76.44	91.07
MRET_0327	ATP adenylyltransferase	133.89	190.93
MRET_0328	ribonuclease P/MRP protein subunit RPP1	8.86	35.26
MRET_0329	uncharacterized protein	241.89	196.38
MRET_0330	DASH complex subunit SPC19	39.33	182.11

MRET_0331	acylpyruvate hydrolase	119	176.49
MRET_0332	mitochondrial pyruvate carrier 2	190.53	431.34
MRET_0333	pre-mRNA polyadenylation factor fip-1	37.7	78.42
MRET_0334	protein ATG11	32.36	60.84
MRET_0335	large subunit ribosomal protein L35	28.14	66.11
MRET_0336	mitochondrial transcription factor MTF1	43.26	84.07
MRET_0337	DnaJ homolog subfamily C member 17	24.57	66.28
MRET_0338	mitochondrial import inner membrane translocase subunit TIM23	32.15	62.89
MRET_0339	ATP-dependent RNA helicase DDX6/DHH1	48.42	65.45
MRET_0340	small plasma membrane protein	61.7	152.46
MRET_0341	histone-binding protein RBBP4	66.2	124.24
MRET_0342	TELO2-interacting protein 1	54.47	51.45
MRET_0343	DNA repair protein RAD16	72.73	128.24
MRET_0344	uncharacterized protein	83.83	71.08
MRET_0345	T-complex protein 1 subunit gamma	80.8	242.52
MRET_0346	H+-transporting ATPase	472.6	393.57
MRET_0347	mitochondrial import inner membrane translocase subunit TIM22	29.09	75.73
MRET_0348	26S proteasome regulatory subunit T2	75.21	180.28
MRET_0349	histone demethylase JARID1	142.58	96.44
MRET_0350	uncharacterized protein	118.33	101.14
MRET_0351	replication factor A2	1094.71	958.53
MRET_0352	V-type H+-transporting ATPase subunit e	309.01	535.95
MRET_0353	dihydrofolate synthase	23.78	72.12
MRET_0354	MYND domain protein (SamB)	650.62	491.53
MRET_0355	mediator of RNA polymerase II transcription subunit 22	7.95	38.4
MRET_0356	uncharacterized protein	350.64	521.96
MRET_0357	thymidylate synthase	168.14	230.85
MRET_0358	protein transport protein SEC24	56.75	77.92
MRET_0359	solute carrier family 25 (mitochondrial phosphate transporter), member 23/24/25/41	25.41	95.68
MRET_0360	glyoxal/methylglyoxal oxidase	186.62	142.09
MRET_0361	glycine-rich RNA binding protein	1055.43	1349.56
MRET_0362	guanine nucleotide-binding protein subunit beta-2-like 1 protein	189.61	653.62
MRET_0363	large subunit ribosomal protein L12e	297.51	997.05
MRET_0364	uncharacterized protein	19.46	37.85
MRET_0365	uncharacterized protein	62.02	81.58
MRET_0366	uncharacterized protein	80.3	46.84
MRET_0367	UDP-glucose 4-epimerase	86.66	89.38

MRET_0368	DUF453 domain protein	598.36	211.38
MRET_0369	uncharacterized protein	10.84	25.97
MRET_0370	centromeric protein E	51.5	46.5
MRET_0371	GTPase binding protein Rid1	7.03	16.11
MRET_0372	nitrogen permease regulator 3-like protein	32.5	43.43
MRET_0373	ATP-dependent DNA helicase MPH1	149.25	167.38
MRET_0374	amidase	50.8	76.68
MRET_0375	protein involved in negative regulation of iron regulon transcription	522.5	506.39
MRET_0376	F-box protein, helicase, 18	131.29	87.89
MRET_0377	COP9 signalosome complex subunit 3	33	62.74
MRET_0378	sister chromatid cohesion protein PDS5	33.52	41.83
MRET_0379	PHD finger and SET domain protein	90.62	104.04
MRET_0380	pre-mRNA-splicing helicase BRR2	37.91	92.79
MRET_0381	protein DJ-1	340.99	459.14
MRET_0382	long-chain acyl-CoA synthetase	1352.44	2435.51
MRET_0383	serine arginine repetitive matrix 2	26.08	55.86
MRET_0384	hydroxymethylglutaryl-CoA reductase (NADPH)	52.82	150.83
MRET_0385	protein TIF31	36.35	39.07
MRET_0386	ESCRT-II complex subunit VPS36	22.78	35.27
MRET_0387	2-polyprenyl-6-hydroxyphenyl methylase/3-demethylubiquinone-9 3-methyltransferase	28.12	32.85
MRET_0388	glycosylphosphatidylinositol transamidase	416.25	523.79
MRET_0389	polyadenylation factor subunit 2	399.35	295.04
MRET_0390	phosphoinositide PI4,5P	1426.66	874.42
MRET_0391	uncharacterized protein	28.83	37.04
MRET_0392	ribonuclease Z	416.41	213.87
MRET_0393	4-hydroxysphinganine ceramide fatty acyl 2-hydroxylase	839.31	794.93
MRET_0394	COX assembly mitochondrial protein 1	19.96	90.12
MRET_0395	ATP-dependent RNA helicase DDX51/DBP6	105.91	90.39
MRET_0396	DnaJ homolog subfamily C member 8	12.06	73.13
MRET_0397	acyl-CoA-dependent ceramide synthase	33.12	83.48
MRET_0398	acetyltransferase (GNAT) family	37.25	50.1
MRET_0399	peptidyl-prolyl isomerase	3348.11	1779.06
MRET_0400	actin-related protein 6	36.73	30.11
MRET_0401	cytochrome c oxidase subunit 19	688.29	351.76
MRET_0402	SET and MYND domain protein	90.87	82.65
MRET_0403	dolichyl-phosphate mannosyltransferase polypeptide 3	191.28	256.89
MRET_0404	uncharacterized protein	54.44	121.43

MRET_0405	myosin V	34.7	43.59
MRET_0406	solute carrier family 12 (potassium/chloride transporters), member 9	29.03	43.98
MRET_0407	pre-mRNA-splicing factor 38A	23.81	39.64
MRET_0408	centromeric protein E	16.84	39.67
MRET_0409	26S proteasome regulatory subunit T5	129.53	150.17
MRET_0410	uncharacterized protein	138.27	125.33
MRET_0411	G-patch domain protein	119.42	87.5
MRET_0412	mediator of RNA polymerase II transcription subunit 4	30.87	54.25
MRET_0413	dual specificity tyrosine-phosphorylation-regulated kinase 2/3/4	36.35	52.21
MRET_0414	importin-4	22.78	60.68
MRET_0415	DnaJ homolog subfamily B member 6	364.1	423.41
MRET_0416	uncharacterized protein	18	27.86
MRET_0417	TBC1 domain family member 5	42.71	47.88
MRET_0418	uncharacterized protein	18.66	18.98
MRET_0419	amphiphysin	668.6	492.97
MRET_0420	translation initiation factor eIF-2B subunit delta	216.46	144.31
MRET_0421	dynein light chain LC8-type	101.11	156.21
MRET_0422	conserved hypothetical protein	112.45	208.46
MRET_0423	ATP-binding protein required for mismatch repair	37.5	41.64
MRET_0424	dienelactone hydrolase	148.28	130
MRET_0425	uncharacterized protein	32.56	60.29
MRET_0426	V-type H+-transporting ATPase subunit G	1174.92	829.94
MRET_0427	symplekin	83.95	78.45
MRET_0428	tryptophan synthase	95.23	93.03
MRET_0429	dienelactone hydrolase	682.85	657.21
MRET_0430	mitochondrial protein import protein ZIM17	505.29	386.17
MRET_0431	solute carrier family 30 (zinc transporter), member 5/7	78.94	166.4
MRET_0432	uncharacterized protein	29.05	30.95
MRET_0433	uncharacterized protein	95.48	100.72
MRET_0434	WD repeat protein 68	156.58	169.75
MRET_0435	DNA replication licensing factor MCM5	36.16	44.88
MRET_0436	prohibitin 2	895.92	650.39
MRET_0437	uncharacterized protein	108.93	78.71
MRET_0438	pyruvate carboxylase	139.34	112.58
MRET_0439	small subunit ribosomal protein S18e	413.52	1056.32
MRET_0440	solute carrier family 25 (mitochondrial iron transporter), member 28/37	212.18	170.24
MRET_0441	uncharacterized protein	253.15	159.38

MRET_0442	uncharacterized protein	56.12	125.67
MRET_0443	translation initiation factor 3 subunit G	39.33	85.41
MRET_0444	V-type H+-transporting ATPase subunit E	358.4	515.33
MRET_0445	large subunit ribosomal protein L54	29.03	77.56
MRET_0446	small G protein signaling modulator 3	9.42	14.66
MRET_0447	cleavage and polyadenylation specificity factor subunit 3	30.87	39.29
MRET_0448	DNA replication licensing factor MCM4	57.18	76.16
MRET_0449	mitotic spindle assembly checkpoint protein MAD2	21.35	66.08
MRET_0450	tyrosine-protein phosphatase SIW14	54.74	107.46
MRET_0451	F-type H+-transporting ATPase subunit g	423.32	372.26
MRET_0452	protein of unknown function (DUF2781)	24.69	35.41
MRET_0453	4a-hydroxytetrahydrobiopterin dehydratase	16.21	29.24
MRET_0454	U5 snRNP protein, DIM1 family	16.7	43.24
MRET_0455	protein DML1	48.81	77.57
MRET_0456	vacuolar protein sorting-associated protein 16	48.78	38.38
MRET_0457	glutamate--cysteine ligase catalytic subunit	70.65	84.72
MRET_0458	ubiquitin carboxyl-terminal hydrolase 1	22.71	33.42
MRET_0459	proteasome maturation protein	134.2	106.22
MRET_0460	ATP-dependent RNA helicase DOB1	9.85	31.69
MRET_0461	nonsense-mediated mRNA decay protein 3	130.35	152.65
MRET_0462	zinc finger protein (RING finger)	383.34	327.59
MRET_0463	transcription initiation factor TFIIE subunit alpha	16.72	37.95
MRET_0464	MFS family protein	71.69	164.09
MRET_0465	ubiquitin-like 1-activating enzyme E1 B	219.81	448.25
MRET_0466	uncharacterized protein	28.15	50.59
MRET_0467	PAPA-1-like conserved region containing protein	29.23	87.65
MRET_0468	ATP-dependent RNA helicase DHX8/PRP22	31.65	54.69
MRET_0469	uncharacterized protein	195.89	114.51
MRET_0470	chitin synthase activator	461.92	360.5
MRET_0471	calmodulin	682.64	902.4
MRET_0472	conserved hypothetical protein	179.62	220.11
MRET_0473	uncharacterized protein	53.75	96.22
MRET_0474	transcription regulator Maf1	204.71	82.28
MRET_0475	serine carboxypeptidase	29.41	101.93
MRET_0476	phosphoribosyl-ATP pyrophosphohydrolase/phosphoribosyl-AMP cyclohydrolase/histidinol dehydrogenase	231.24	179.61
MRET_0477	uncharacterized protein	103.09	46.03
MRET_0478	carnitine O-acetyltransferase	92.14	63.16

MRET_0479	SNF1-activating kinase 1	228.02	119.26
MRET_0480	XPG I-region protein	100.44	102.04
MRET_0481	large subunit ribosomal protein L11e	189.97	837.75
MRET_0482	uncharacterized protein	203.98	152.9
MRET_0483	ATP-dependent RNA helicase DDX3X	157.43	131.04
MRET_0484	deaminated glutathione amidase	388.79	121.69
MRET_0485	nucleoside hydrolase	62.05	82.18
MRET_0486	small nuclear ribonucleoprotein B and B'	657.63	691.94
MRET_0487	tryptophan aminotransferase	390.39	322.5
MRET_0488	DASH complex subunit Hsk3 like protein	468.64	324.72
MRET_0489	syntaxin-binding protein 1	60.18	75.02
MRET_0490	DNA topoisomerase 2-associated protein PAT1	61.28	98.37
MRET_0491	BRCA1 C terminus (BRCT) domain protein	64.19	73.3
MRET_0492	solute carrier family 26 (sodium-independent sulfate anion transporter), member 11	144.03	143.95
MRET_0493	nucleotide exchange factor	22.5	45.51
MRET_0494	ethanolaminephosphotransferase	29.86	56.29
MRET_0495	condensin complex subunit 2	17.52	50.2
MRET_0496	2-acylglycerol O-acyltransferase 2	54.79	68.34
MRET_0497	histidine phosphatase	19.85	33.96
MRET_0498	lysophospholipase	1443.06	1052.97
MRET_0499	translation factor GUF1, mitochondrial	34.84	48.08
MRET_0500	mRNA export factor	269.6	371.53
MRET_0501	large subunit ribosomal protein L24e	424.31	1072.06
MRET_0502	serine palmitoyltransferase	105.96	138.47
MRET_0503	aldehyde dehydrogenase	114.56	104.32
MRET_0504	COP9 signalosome complex subunit 5	24.65	37.22
MRET_0505	G2/mitotic-specific cyclin 1/2	96.43	116.47
MRET_0506	MIS18 kinetochore protein homolog A	12.07	18.05
MRET_0507	mRNA-decapping enzyme 1B	48.26	63.97
MRET_0508	replication fork protection complex subunit Tof1/Swi1	124.94	66.98
MRET_0509	DNA polymerase subunit cdc27	28.57	42.9
MRET_0510	ATP-dependent RNA helicase DDX19/DBP5	70.5	71.53
MRET_0511	uncharacterized protein	36.31	40.47
MRET_0512	uncharacterized protein	373.74	449.29
MRET_0513	CRAL/TRIO domain protein	236.31	182.17
MRET_0514	F-type H+-transporting ATPase subunit k	189.82	266.06
MRET_0515	uncharacterized protein	106.78	108.1

MRET_0516	large subunit ribosomal protein L32e	257.93	878.62
MRET_0517	large subunit ribosomal protein L13Ae	107.09	438.12
MRET_0518	uncharacterized protein	143.22	102.44
MRET_0519	DUF803 domain protein	18.98	30.02
MRET_0520	phosphatidylinositol phospholipase C, delta	192.26	118.9
MRET_0521	plasminogen activator inhibitor 1 RNA-binding protein	104.36	237.61
MRET_0522	small subunit ribosomal protein S3e	206.87	364.35
MRET_0523	dynamin-like GTPase MGM1, mitochondrial	907.65	571.84
MRET_0524	3-keto steroid reductase	121.49	60.19
MRET_0525	RNA exonuclease 1	38.09	35.75
MRET_0526	uncharacterized protein	136.22	112.67
MRET_0527	MAGE family protein	24.79	55.29
MRET_0528	phospholipase carboxylesterase	18.99	40.14
MRET_0529	HMG (high mobility group) box protein	257.97	397.19
MRET_0530	26S proteasome regulatory subunit N12	31.85	59.46
MRET_0531	uncharacterized protein	11.79	32.11
MRET_0532	cytochrome b5	140.66	192.44
MRET_0533	pyruvate dehydrogenase phosphatase	84.75	94.3
MRET_0534	vesicle-fusing ATPase	239.62	130.4
MRET_0535	vacuolar import and degradation protein	475.44	383.27
MRET_0536	translation initiation factor 3 subunit D	52.09	100.22
MRET_0537	uncharacterized protein	51.36	47.62
MRET_0538	CDP-diacylglycerol---glycerol-3-phosphate 3-phosphatidyltransferase	105.16	61.77
MRET_0539	uncharacterized protein	189.37	149.06
MRET_0540	uncharacterized protein	173.53	166.04
MRET_0541	tRNA (adenine-N(1)-)-methyltransferase non-catalytic subunit	143.44	249.37
MRET_0542	uncharacterized protein	128.62	119.24
MRET_0543	U3 small nucleolar RNA-associated protein MPP10	1237.71	5947.52
MRET_0544	bifunctional dethiobiotin synthetase/adenosylmethionine---8-amino-7-oxononanoate aminotransferase	114.62	77.84
MRET_0545	AHNAK nucleoprotein	44.23	63.99
MRET_0546	anaphase-promoting complex subunit 8	9.39	28.53
MRET_0547	cytoplasm to vacuole targeting protein	56.19	124.67
MRET_0548	large subunit ribosomal protein L38e	71.91	384.42
MRET_0549	small subunit ribosomal protein S14e	117.47	510.61
MRET_0550	nuclear movement protein NUDC	50.44	155.75
MRET_0551	DNA repair and recombination protein RAD54B	110.27	95.94
MRET_0552	outer membrane protein insertion porin family	148	148.28

MRET_0553	DNA-directed RNA polymerase III subunit RPC8	19.42	53.65
MRET_0554	GTPase-activating protein SAC7	245.93	315.71
MRET_0555	vacuolar protein sorting-associated protein 8	103.39	94.52
MRET_0556	AFG3 family protein	468.92	312.24
MRET_0557	SWI/SNF chromatin-remodeling complex subunit SWI1	48.25	53.73
MRET_0558	26S proteasome regulatory subunit N1	67.13	113.97
MRET_0559	DNA-3-methyladenine glycosylase II	87.45	113.67
MRET_0560	AHNAK nucleoprotein	112.66	119.6
MRET_0561	solute carrier family 32 (vesicular inhibitory amino acid transporter)	250.67	129.41
MRET_0562	nuclear pore complex protein Nup62	76.24	131.56
MRET_0563	20S proteasome subunit beta 7	295.2	409.93
MRET_0564	dipeptidylpeptidase	50.85	57.36
MRET_0565	replication factor C subunit 3/5	53.14	103.69
MRET_0566	alanine transaminase	122.59	236.81
MRET_0567	uncharacterized protein	32.77	39.12
MRET_0568	small subunit ribosomal protein S11	329.42	290.54
MRET_0569	small subunit ribosomal protein S9	121.03	161.49
MRET_0570	uncharacterized protein	265.3	193.01
MRET_0571	phosphatidylinositol 4-kinase A	82.34	47.52
MRET_0572	coiled-coil domain protein 12	64.3	65.07
MRET_0573	mitochondrial splicing suppressor protein 51	703.39	391.93
MRET_0574	urease accessory protein	835.56	530.45
MRET_0575	endoplasmic reticulum-based factor for assembly of V-ATPase	143.53	88.7
MRET_0576	peroxisome-assembly ATPase	81.62	60.19
MRET_0577	checkpoint serine/threonine-protein kinase	24.19	58.91
MRET_0578	ribosome biogenesis protein SSF1/2	260.3	358.3
MRET_0579	T-complex protein 1 subunit alpha	52.65	95.91
MRET_0580	calcium/calmodulin-dependent protein kinase I	1459.68	716.77
MRET_0581	protein of unknown function (DUF2423)	38.94	38.25
MRET_0582	CDK inhibitor PHO81	201.8	172.51
MRET_0583	exportin-1	324.34	235.74
MRET_0584	golgi SNAP receptor complex member 1	187.46	105.34
MRET_0585	DNA binding regulatory protein AmdX	83.79	99.15
MRET_0586	bZIP transcription factor	126.32	34.01
MRET_0587	monothiol glutaredoxin	975.59	906.18
MRET_0588	pre-rRNA-processing protein TSR4	50.22	89.77
MRET_0589	methylenetetrahydrofolate reductase (NADPH)	58.84	70.99

MRET_0590	uncharacterized protein	80.46	75.33
MRET_0591	origin recognition complex subunit 1	38.28	50.42
MRET_0592	alpha-1,2-mannosyltransferase	54.87	61.07
MRET_0593	phospholipase C	104.27	116.39
MRET_0594	uncharacterized protein	248.98	184.11
MRET_0595	IPT/TIG domain protein	365.73	220.47
MRET_0596	uncharacterized protein	305.2	238.45
MRET_0597	protein SEY1	157.31	141.12
MRET_0598	NTF2 and RRM domain protein	46.73	43.19
MRET_0599	dynamin 1-like protein	680.26	419.21
MRET_0600	Cut9 interacting protein Scn1	83.95	121.02
MRET_0601	G2 mitotic-specific protein	30.49	33.53
MRET_0602	zinc finger HIT domain protein 1	5.16	16.5
MRET_0603	uncharacterized protein	24.49	30.2
MRET_0604	uncharacterized protein	106.21	47.9
MRET_0605	protein farnesyltransferase subunit beta	81.84	48.85
MRET_0606	protein disulfide-isomerase A6	33.92	47.91
MRET_0607	uncharacterized protein	13.54	21.67
MRET_0608	protein of unknown function (DUF3405)	480.71	227.25
MRET_0609	dolichyl-diphosphooligosaccharide---protein glycosyltransferase	87.17	104.22
MRET_0610	peroxin-6	192.64	92.2
MRET_0611	malate dehydrogenase	445.03	348.13
MRET_0612	conserved oligomeric golgi complex subunit 7	90.1	60.4
MRET_0613	3-deoxy-7-phosphoheptulonate synthase	179.23	210.36
MRET_0614	importin subunit beta-1	68.88	49.79
MRET_0615	SWI/SNF-related matrix-associated actin-dependent regulator of chromatin subfamily A member 5	147.63	101.86
MRET_0616	MICOS complex subunit MIC12	143.03	154.77
MRET_0617	DUF2407 ubiquitin-like domain protein	77.38	74.4
MRET_0618	kexin	94.64	83.72
MRET_0619	calnexin	211.41	181.2
MRET_0620	argininosuccinate lyase	290.57	139.49
MRET_0621	DnaJ homolog subfamily A member 2	310.55	262.69
MRET_0622	25S rRNA (cytosine2278-C5)-methyltransferase	11.59	12.31
MRET_0623	mannosyl-glycoprotein endo-beta-N-acetylglucosaminidase	55.44	42.48
MRET_0624	SNARE domain protein	109.66	94.65
MRET_0625	transcription initiation factor TFIID subunit 6	55.61	70.3
MRET_0626	methylenetetrahydrofolate dehydrogenase (NAD+)	28.92	34.11

MRET_0627	translation initiation factor 4B	212.5	103.52
MRET_0628	ubiquitin-protein ligase involved in ER-associated protein degradation	142	108.46
MRET_0629	phospholipid-translocating ATPase	80.73	45.76
MRET_0630	vacuolar protein sorting-associated protein 35	151.86	79.89
MRET_0631	large subunit ribosomal protein L18Ae	712.09	1386.04
MRET_0632	RNA-binding protein 26	36.99	70.59
MRET_0633	large subunit ribosomal protein L40e	1102.26	2167.89
MRET_0634	pre-60S factor REI1	241.64	292.62
MRET_0635	CUE domain protein	51.17	27.35
MRET_0636	stress response protein NST1	119.53	112.16
MRET_0637	mitochondrial FAD-linked sulfhydryl oxidase	419.58	168.86
MRET_0638	ubiquitin carboxyl-terminal hydrolase 8	16.29	23.66
MRET_0639	chromatin structure-remodeling complex protein RSC7	53.08	60.02
MRET_0640	uncharacterized protein	181.97	108.04
MRET_0641	translation initiation factor 2 subunit 3	98.6	174.97
MRET_0642	golgi traffic protein SFT2	64.64	75.7
MRET_0643	F-type H+-transporting ATPase subunit h	617.26	946.61
MRET_0644	ATPase inhibitor, mitochondrial	2746.03	2255.54
MRET_0645	regulator of nonsense transcripts	100.05	76.56
MRET_0646	OTU domain protein 3	14.22	27.89
MRET_0647	anaphase-promoting complex subunit 1	7.15	8.54
MRET_0648	18S rRNA (adenine1779-N6/adenine1780-N6)-dimethyltransferase	14.11	29.76
MRET_0649	AP complex subunit beta	44.28	57.16
MRET_0650	protein of unknown function (DUF3074)	234.99	217.19
MRET_0651	uncharacterized protein	345.88	282.93
MRET_0652	aldose 1-epimerase	20.82	23.58
MRET_0653	dolichyl-phosphate-mannose-protein mannosyltransferase	32.62	47.23
MRET_0654	uncharacterized protein	42.19	29.92
MRET_0655	long-chain-alcohol oxidase	580.62	512.15
MRET_0656	7SK snRNA methylphosphate capping enzyme	510.19	457.97
MRET_0657	DNA-directed RNA polymerase, mitochondrial	79.39	81.4
MRET_0658	uncharacterized protein	25.76	20.58
MRET_0659	uncharacterized protein	20.5	68.14
MRET_0660	superoxide dismutase, Fe-Mn family	236.57	272.63
MRET_0661	exportin-4	120.6	70.65
MRET_0662	histone-lysine N-methyltransferase SETD2	49.58	60.09
MRET_0663	DNA-repair protein complementing XP-A cells	14.76	27.88

MRET_0664	uncharacterized protein	17.85	19.27
MRET_0665	prolactin regulatory element-binding protein	56.17	74.6
MRET_0666	sphingolipid 8-(E)-desaturase	285.95	117.27
MRET_0667	sphingomyelin phosphodiesterase	115.85	80.71
MRET_0668	sphingomyelin phosphodiesterase	26.72	59.33
MRET_0669	glutaredoxin-like protein	3.6	7.03
MRET_0670	SUR7/PalI family protein	3.89	31.44
MRET_0671	leucyl-tRNA synthetase	48.46	56.72
MRET_0672	mediator of RNA polymerase II transcription subunit 12, fungi type	49.29	32.17
MRET_0673	uncharacterized protein	76.93	63.23
MRET_0674	actin related protein 2/3 complex, subunit 4	357.25	307.03
MRET_0675	chromodomain-helicase-DNA-binding protein 1	37.51	39.05
MRET_0676	nuclear GTP-binding protein	40.74	51.84
MRET_0677	threonine dehydratase	281.52	108.93
MRET_0678	ABC multidrug transporter	46.31	68.12
MRET_0679	ferricrocin synthase	31.1	157.42
MRET_0680	ATP-dependent RNA helicase DDX54/DBP10	69.89	61.48
MRET_0681	uncharacterized protein	50.26	45.52
MRET_0682	serine/threonine-protein phosphatase PPG1	25.36	48.98
MRET_0683	pyridoxal phosphate phosphatase PHOSPHO2	43.52	71.07
MRET_0684	RhoGEF domain protein	16.99	29.05
MRET_0685	peptidoglycan-binding domain protein	38.53	43.49
MRET_0686	uncharacterized protein	33.11	68.82
MRET_0687	protein of unknown function (DUF1168)	36.16	82.79
MRET_0688	2-methylcitrate dehydratase	110.67	63.42
MRET_0689	cysteine synthase A	69.62	122.13
MRET_0690	dual specificity protein kinase YAK1	192.8	115.06
MRET_0691	nuclear distribution protein nudE homolog 1	66.62	68.04
MRET_0692	ER to golgi transport-related protein	48.9	75.75
MRET_0693	uncharacterized protein	12.46	14.22
MRET_0694	nuclear GTP-binding protein	162.86	899.77
MRET_0695	20S proteasome subunit beta 1	74.73	134.27
MRET_0696	serine/threonine-protein phosphatase 2B regulatory subunit	100.56	210.98
MRET_0697	small subunit ribosomal protein S13	35.66	107.59
MRET_0698	serine/threonine-protein kinase	50.69	47.55
MRET_0699	acetoacetyl-CoA synthetase	55.58	55.23
MRET_0700	H/ACA ribonucleoprotein complex subunit 1	180.77	260.92

MRET_0701	urea-proton symporter	119.04	93.77
MRET_0702	vacuolar protein sorting-associated protein 26	40.44	54.07
MRET_0703	autophagy-related protein 27	128.23	102.41
MRET_0704	di- and tripeptidase	18.35	30.87
MRET_0705	DNA-directed RNA polymerase III subunit RPC6	29.45	47.78
MRET_0706	DNA topoisomerase I	29.52	72.45
MRET_0707	transcription activator of gluconeogenesis ERT1	4.86	23.57
MRET_0708	multifunctional methyltransferase subunit TRM112	30.52	106.73
MRET_0709	phosphoenolpyruvate carboxykinase (ATP)	325.68	892.72
MRET_0710	mitochondrial pyruvate carrier 1	101.83	602.41
MRET_0711	DUF803 domain membrane protein	1.67	26.25
MRET_0712	adenylate cyclase	3.38	24.72
MRET_0713	solute carrier family 25 (mitochondrial phosphate transporter), member 3	187.77	189.07
MRET_0714	sulfate adenylyltransferase	49.39	81.68
MRET_0715	histone-like transcription factor (CBF/NF-Y)	238.81	258.91
MRET_0716	ATP-dependent DNA helicase 2 subunit 1	77.12	40.26
MRET_0717	peroxisomal 2,4-dienoyl-CoA reductase	571.83	266.27
MRET_0718	large subunit ribosomal protein L39e	188.88	667.18
MRET_0719	thiamin pyrophosphokinase-related protein	312.93	284.42
MRET_0720	actin cortical patch component	270.44	166.16
MRET_0721	peroxin-5	443.49	119.81
MRET_0722	HATPase_c domain protein	17.49	19.34
MRET_0723	diphthamide biosynthesis protein 2	16.11	31.89
MRET_0724	MRC1-like domain protein	23.76	38.12
MRET_0725	uncharacterized protein	12.39	31.57
MRET_0726	phosphatidylinositol glycan, class P	268.8	236.92
MRET_0727	cell division control protein	38.3	73.45
MRET_0728	tyrosyl-tRNA synthetase	171.68	166.98
MRET_0729	uncharacterized protein	72.13	84.4
MRET_0730	mitochondrial import inner membrane translocase subunit TIM17	236.33	302.28
MRET_0731	beclin	55.02	133.97
MRET_0732	F-type H+-transporting ATPase subunit delta	402.03	524.07
MRET_0733	ATP-binding cassette, subfamily D (ALD), peroxisomal long-chain fatty acid import protein	246.2	167.12
MRET_0734	ornithine decarboxylase antizyme	342.66	365.34
MRET_0735	protein transport protein SEC61 subunit alpha	152.7	321.71
MRET_0736	DNA-directed RNA polymerases I, II, and III subunit RPABC5	104.63	265.92
MRET_0737	uncharacterized protein	90.26	122.19

MRET_0738	E3 ubiquitin-protein ligase BRE1	16.24	38.85
MRET_0739	sphinganine-1-phosphate aldolase	408.63	288.13
MRET_0740	transcription initiation factor TFIIA large subunit	793.21	714.76
MRET_0741	chromatin remodeling	104.33	120.74
MRET_0742	Hamartin protein	93.5	59.37
MRET_0743	glutaminyl-tRNA synthetase	48.73	45.91
MRET_0744	transmembrane 9 superfamily member 2/4	93.13	109.7
MRET_0745	trafficking protein particle complex subunit 1	307.71	159.35
MRET_0746	diphosphomevalonate decarboxylase	81.79	77.78
MRET_0747	large subunit ribosomal protein L27e	119.36	492.46
MRET_0748	small subunit ribosomal protein S23e	572.37	1437.31
MRET_0749	mitochondrial protein required for assembly of cytochrome bc1 complex	384.21	361.49
MRET_0750	ribosome biogenesis protein BRX1	157.54	267.59
MRET_0751	uncharacterized protein	6.3	7.34
MRET_0752	ergosterol biosynthesis protein	639.75	442.54
MRET_0753	uncharacterized protein	30.48	23.67
MRET_0754	uncharacterized protein	9.86	13.09
MRET_0755	lysyl-tRNA synthetase, class II	40.26	70.31
MRET_0756	endonuclease LCL3	188.23	152.84
MRET_0757	zinc finger protein (RING finger)	289.98	194.29
MRET_0758	phosphoribosylformylglycinamidine synthase	342.74	115.13
MRET_0759	transcriptional adapter 2-alpha	29.55	65.7
MRET_0760	splicing factor U2AF 35 kDa subunit	67.54	168.99
MRET_0761	Rab family GTPase	127.71	204.55
MRET_0762	palmitoyltransferase	44.98	71.89
MRET_0763	uncharacterized protein	43.04	117
MRET_0764	uncharacterized protein	28.79	43.95
MRET_0765	pentatricopeptide repeat protein	7.88	19.89
MRET_0766	phosphatidylinositol 4-kinase type 2	37.23	36.99
MRET_0767	RNA polymerase I specific initiation factor	290.37	113.57
MRET_0768	protein phosphatase PTC1	713.67	658.99
MRET_0769	required for meiotic nuclear division 5 homolog	320.78	197.28
MRET_0770	ATP synthase mitochondrial F1 complex assembly factor 1	29.6	35.35
MRET_0771	phosphatidylinositol glycan, class W	18.15	30.7
MRET_0772	F-type H+-transporting ATPase subunit O	499.14	705.96
MRET_0773	translation initiation factor 3 subunit C	73.05	101.7
MRET_0774	regulatory subunit for Cdc7p protein kinase	285.99	127.18

MRET_0775	ribonuclease HI	23.23	25.69
MRET_0776	NADPH-ferrihemoprotein reductase	80.42	113.79
MRET_0777	DNA excision repair protein ERCC-1	19.91	44.86
MRET_0778	ribosome biogenesis protein NSA2	44.34	131.25
MRET_0779	protein transport protein YIP1	35.48	65.74
MRET_0780	DNA mismatch repair protein MLH1	56.05	82.56
MRET_0781	uncharacterized protein	43.15	52.56
MRET_0782	cyclin H	408.01	186.71
MRET_0783	exosome complex component RRP45	318.17	154.06
MRET_0784	uncharacterized protein	2841.3	1910.96
MRET_0785	serine/threonine-protein phosphatase 2A activator	164.47	92.05
MRET_0786	WD domain, G-beta repeat protein	15.58	24.52
MRET_0787	TBC1 domain family member 10	69.1	120.36
MRET_0788	NIMA-related kinase 2	18.3	62.31
MRET_0789	protein FMP21	324.18	292.87
MRET_0790	SRP40, C-terminal domain protein	38.17	89.2
MRET_0791	signal peptidase complex subunit 3	56.42	123.83
MRET_0792	uncharacterized protein	237.98	223
MRET_0793	oxysterol-binding protein 1	125.27	122.07
MRET_0794	DASH complex subunit Duo1	87.44	170.31
MRET_0795	golgi pH regulator	22.99	59.6
MRET_0796	protein EFR3	48.09	53.35
MRET_0797	serine hydrolase (FSH1)	27.39	39.93
MRET_0798	UV excision repair protein RAD23	25.06	59.97
MRET_0799	homeobox domain protein	29.9	65
MRET_0800	carbamoyl-phosphate synthase small subunit	75.44	174.67
MRET_0801	uncharacterized protein	15.05	39.72
MRET_0802	U4/U6.U5 tri-snRNP component SNU23	21.22	38.83
MRET_0803	isoleucyl-tRNA synthetase	59.39	75.97
MRET_0804	F-type H+-transporting ATPase subunit gamma	434.08	489.5
MRET_0805	translation initiation factor 2 subunit 2	248.14	434.74
MRET_0806	HIT finger domain protein	23.13	40.99
MRET_0807	SAP domain ribonucleoprotein	46.76	131.14
MRET_0808	uncharacterized protein	62.82	146.81
MRET_0809	uncharacterized protein	93.4	156.01
MRET_0810	ADP-ribosylation factor	22.38	44.07
MRET_0811	SWI/SNF-related matrix-associated actin-dependent regulator of chromatin subfamily A	19.55	36.22

MRET_0812	geranylgeranyl transferase type-2 subunit alpha	30.13	98.47
MRET_0813	protein Yae1	32.11	144.53
MRET_0814	emopamil binding protein	61.46	148.37
MRET_0815	uncharacterized protein	451.68	689.26
MRET_0816	uncharacterized protein	198.57	191.26
MRET_0817	histone acetyltransferase	25.16	101.84
MRET_0818	phosphatidylinositol-binding clathrin assembly protein	43.03	38.02
MRET_0819	uncharacterized protein	11.03	46.35
MRET_0820	25S rRNA (uracil2634-N3)-methyltransferase	29.93	40.17
MRET_0821	DnaJ homolog subfamily C member 3	54.97	99.99
MRET_0822	uncharacterized protein	453.73	409.24
MRET_0823	iron-sulfur cluster assembly protein ISA1	410.2	241.46
MRET_0824	GTPase	34.58	33.85
MRET_0825	membrane protein TMS1	64.92	55.46
MRET_0826	folic acid synthesis protein	37.51	58.75
MRET_0827	uncharacterized protein	104.31	142.88
MRET_0828	DNA polymerase epsilon subunit 2	317	286.61
MRET_0829	phosphatidylinositol glycan, class A	103.67	141.65
MRET_0830	alanyl-tRNA synthetase	34.11	59.72
MRET_0831	zinc finger protein, C2H2 type	517.82	206.12
MRET_0832	3-dehydrosphinganine reductase	40.92	20.39
MRET_0833	uncharacterized protein	43.33	69.06
MRET_0834	uncharacterized protein	685.46	543.67
MRET_0835	cathepsin D	217.94	143.11
MRET_0836	mRNA guanylyltransferase	23.88	62.44
MRET_0837	tRNA-splicing endonuclease subunit Sen2	29.66	42.87
MRET_0838	dentin sialophosphoprotein	1044.49	747.63
MRET_0839	programmed cell death protein 5	27.38	153.21
MRET_0840	conserved hypothetical protein	31.1	85.07
MRET_0841	RanBD	134	125.37
MRET_0842	translation initiation factor eIF-2B subunit epsilon	86.14	104.34
MRET_0843	phosphopantothenoylcysteine decarboxylase	51.53	38.76
MRET_0844	L-galactose dehydrogenase	193.03	116.14
MRET_0845	phosphoglycerate kinase	1515.02	839.74
MRET_0846	AMP deaminase	27.3	27.17
MRET_0847	merozoite surface protein msp-1	247.67	117.84
MRET_0848	zinc finger protein	108.51	61.25

MRET_0849	pyridoxine kinase	67.26	76.51
MRET_0850	uncharacterized protein	7.5	32.14
MRET_0851	DNA polymerase alpha subunit A	59.5	41.79
MRET_0852	centromere protein Scm3	57.96	67.6
MRET_0853	uncharacterized protein	194.5	181.33
MRET_0854	large subunit ribosomal protein L35Ae	359.24	891.7
MRET_0855	bud site selection protein 20	35.47	57.4
MRET_0856	FAD dependent oxidoreductase	170.71	213.57
MRET_0857	DUF1014 domain protein	1039.27	718.56
MRET_0858	uncharacterized protein	118.08	80.42
MRET_0859	large subunit ribosomal protein L27	215.08	215.75
MRET_0860	mitochondrial import inner membrane translocase subunit TIM23	83.38	152.94
MRET_0861	uncharacterized protein	630.76	388.9
MRET_0862	multi-transmembrane subunit of the DSC ubiquitin ligase complex	24.5	56.8
MRET_0863	fungal domain of unknown function (DUF1712)	61.25	53.11
MRET_0864	uncharacterized protein	546.93	232.93
MRET_0865	prephenate dehydrogenase (NADP+)	94.47	54.58
MRET_0866	PX domain protein	77.71	49.69
MRET_0867	beta-1,4-N-acetylglucosaminyltransferase	33.7	34.48
MRET_0868	uncharacterized protein	4.04	31.78
MRET_0869	syntaxin 1B/2/3	207.16	217.75
MRET_0870	large subunit ribosomal protein L15	49.85	75.22
MRET_0871	ATP-dependent RNA helicase DDX23/PRP28	245.81	152.35
MRET_0872	acetyl-CoA carboxylase, biotin containing enzyme	77.99	40.93
MRET_0873	ubiquinol-cytochrome c reductase cytochrome c1 subunit	353.55	248.38
MRET_0874	4-amino-4-deoxychorismate lyase	65.14	67.9
MRET_0875	crossover junction endonuclease MUS81	91.13	94.29
MRET_0876	small subunit ribosomal protein S15e	121.11	586.72
MRET_0877	large subunit ribosomal protein LP2	939.91	1753.94
MRET_0878	Rho GTPase-activating protein RGD1	114.56	120.05
MRET_0879	cryptococcal mannosyltransferase 1	95.06	91.97
MRET_0880	uncharacterized protein	22.66	34.39
MRET_0881	DUF803 domain membrane protein	146.06	71.29
MRET_0882	PPR repeat containing protein	93.96	33.92
MRET_0883	uncharacterized protein	498.6	810.14
MRET_0884	urea transporter	414.23	895.59
MRET_0885	transcriptional adapter 3	25.4	50.83

MRET_0886	nucleotide exchange factor for Gsp1p	20.28	23.85
MRET_0887	fatty acid elongase 3	158.67	64.57
MRET_0888	ubiquitin-conjugating enzyme E2 D	3274.93	3163.48
MRET_0889	SANT domain protein	93.1	66.14
MRET_0890	FMN binding oxidoreductase	1138.25	663.24
MRET_0891	origin recognition complex subunit 5	53.84	52.76
MRET_0892	uncharacterized protein	18.09	25.54
MRET_0893	member of the NineTeen Complex	162.66	119.38
MRET_0894	glyoxylate/hydroxypyruvate reductase	211.13	205.72
MRET_0895	ubiquinol-cytochrome c reductase core subunit 2	565.64	366.4
MRET_0896	DNA/RNA-binding protein KIN17	1810.19	723.3
MRET_0897	glycine hydroxymethyltransferase	179.36	554.4
MRET_0898	mitochondrial exoribonuclease Cyt-4	97.47	103.54
MRET_0899	DNA damage-inducible protein 1	109.39	146.32
MRET_0900	pre-mRNA-splicing factor ATP-dependent RNA helicase DHX38/PRP16	138.3	92.07
MRET_0901	nuclear pore complex protein Nup37	32.62	33.26
MRET_0902	E3 ubiquitin-protein ligase TRIP12	370.09	188.73
MRET_0903	CLIP-associating protein 1/2	252.72	158.99
MRET_0904	replication factor C subunit 3/5	67.26	76.52
MRET_0905	uncharacterized protein	54.47	50.96
MRET_0906	sorting nexin-41/42	163.65	131.33
MRET_0907	ubiquitin	262.02	104.81
MRET_0908	aspartate aminotransferase, cytoplasmic	1083.88	731.69
MRET_0909	non-structural maintenance of chromosomes element 1	53.49	38.63
MRET_0910	methylenetetrahydrofolate reductase (NADPH)	32.66	28.28
MRET_0911	actin-related protein 8	91.55	70.04
MRET_0912	SAP domain protein	367.15	236.02
MRET_0913	imidazoleglycerol-phosphate dehydratase	181.93	129.35
MRET_0914	dynein intermediate chain, cytosolic	137.21	121.52
MRET_0915	uncharacterized protein	154.84	141.16
MRET_0916	SAC3 GANP domain protein	109.14	87.27
MRET_0917	homoserine kinase	116.65	138.87
MRET_0918	kinetochore protein Nuf2	48.47	116.19
MRET_0919	glutathione-dependent oxidoreductase	619.81	433.09
MRET_0920	ubiquitin carboxyl-terminal hydrolase 4/11	66.17	56.32
MRET_0921	peptidase inhibitor activity protein	618.34	4126.24
MRET_0922	glycosyl transferase family protein	93.58	60.31

MRET_0923	acylglycerol lipase	142.31	141.33
MRET_0924	histone H3	218.28	125.65
MRET_0925	histone H4	4208.07	2174.05
MRET_0926	acetyl-CoA acyltransferase 1	716.55	394.34
MRET_0927	cyclin-dependent protein kinase complex component	12.19	34.03
MRET_0928	uncharacterized protein	31.49	87.3
MRET_0929	HMG (high mobility group) box protein	59.1	121.23
MRET_0930	secretory lipase	499.03	824.46
MRET_0931	annexin A7	512.01	288.68
MRET_0932	uncharacterized protein	186.01	96.26
MRET_0933	glutamate N-acetyltransferase/amino-acid N-acetyltransferase	32.1	40.76
MRET_0934	solute carrier family 39 (zinc transporter), member 1/2/3	65.93	63.67
MRET_0935	solute carrier family 39 (zinc transporter), member 1/2/3	59.53	11.95
MRET_0936	cell cycle control protein	36.4	67.76
MRET_0937	small subunit ribosomal protein S4e	119.97	531.01
MRET_0938	DnaJ domain protein	457.77	551.68
MRET_0939	nucleus protein	96.19	69.57
MRET_0940	SAC3 GANP domain protein	127.96	85.06
MRET_0941	solute carrier family 35, member E1	16.8	23.99
MRET_0942	uncharacterized protein	203.49	114.98
MRET_0943	ATP-dependent RNA helicase DDX10/DBP4	158.18	125.83
MRET_0944	mitochondrial carrier protein	87.5	138.8
MRET_0945	multidrug resistance protein, MATE family	33.11	36.71
MRET_0946	CTD kinase subunit beta	37.4	33
MRET_0947	DnaJ homolog subfamily C member 9	68	79.74
MRET_0948	uncharacterized protein	1692.05	1124.25
MRET_0949	uncharacterized protein	1679.59	2251.19
MRET_0950	AHA1 family protein	346.95	448.37
MRET_0951	26S proteasome regulatory subunit N6	76.36	142.91
MRET_0952	26S proteasome regulatory subunit N7	287.48	215.4
MRET_0953	UBA TS-N domain protein	144.31	85.95
MRET_0954	signal transducing adaptor molecule	180.07	181.86
MRET_0955	queuosine salvage protein	27.35	28.32
MRET_0956	DEAD/DEAH box helicase	28.99	39.3
MRET_0957	trafficking protein particle complex subunit 11	20.06	25.04
MRET_0958	midasin	173.91	137.42
MRET_0959	U3 small nucleolar RNA-associated protein 21	260.38	117.23

MRET_0960	ATP-dependent RNA helicase DDX18/HAS1	22.91	51.82
MRET_0961	periodic tryptophan protein 2	108.15	85.23
MRET_0962	Sec7 domain protein	11	33.89
MRET_0963	cyclin (Pcl1)	11.66	67.51
MRET_0964	VanZ domain protein	126.98	291.39
MRET_0965	uncharacterized protein	140.55	222.15
MRET_0966	fructose-1,6-bisphosphatase I	473.71	336.68
MRET_0967	protoporphyrinogen/coproporphyrinogen III oxidase	190.65	89.46
MRET_0968	ubiquinol-cytochrome c reductase subunit 7	325.26	378.43
MRET_0969	DUF300 domain protein	31.6	53.06
MRET_0970	RAD50-interacting protein 1	54.35	68.73
MRET_0971	uncharacterized protein	635.25	660.87
MRET_0972	translin family protein	109.01	126.38
MRET_0973	uncharacterized protein	11.79	14.32
MRET_0974	uncharacterized protein	357.21	252.37
MRET_0975	COP9 signalosome complex subunit 6	110.75	83.55
MRET_0976	DNA replication licensing factor MCM3	156.15	150.39
MRET_0977	Ras-related protein Rab-11A	179.74	286.91
MRET_0978	peroxin-4	64.98	63.34
MRET_0979	mitogen-activated protein kinase kinase kinase	98.63	87.58
MRET_0980	transportin-3	55.32	83.52
MRET_0981	vacuolar protein-sorting-associated protein 4	257.07	325.94
MRET_0982	phospholipase D1/2	15.5	29.86
MRET_0983	citrate synthase	168.01	175.27
MRET_0984	uroporphyrinogen-III synthase	32.81	57.83
MRET_0985	uncharacterized protein	43.29	52.97
MRET_0986	golgi vesicular membrane trafficking protein	91.26	102.68
MRET_0987	rhomboid family membrane protein	100.91	107.71
MRET_0988	uncharacterized protein	36.34	51.95
MRET_0989	LIM domain protein	11.75	24.38
MRET_0990	enhanced filamentous growth protein 1	98.17	103.36
MRET_0991	uncharacterized protein	30.93	53.26
MRET_0992	uncharacterized protein	53.76	78.42
MRET_0993	1,3-beta-glucan synthase	54.75	89.61
MRET_0994	sterol 22-desaturase	55.13	92.22
MRET_0995	serine/threonine-protein kinase	117.06	93.91
MRET_0996	transcription initiation factor TFIIH subunit 3	30.34	42.67

MRET_0997	cyclin	19.84	26.39
MRET_0998	charged multivesicular body protein 3	15.43	20.32
MRET_0999	histone H2B	279.61	315.19
MRET_1000	histone H2A	529	370.9
MRET_1001	lysine-specific demethylase 8	27.24	24.82
MRET_1002	isocitrate dehydrogenase (NAD+) IDH1	267.38	345.42
MRET_1003	isocitrate dehydrogenase (NAD+) IDH2	373.87	455.25
MRET_1004	component of the cleavage and polyadenylation factor I	442.45	472.92
MRET_1005	large subunit ribosomal protein L18e	223.31	488.5
MRET_1006	LisH domain protein	233.71	121.21
MRET_1007	AP-1 complex subunit mu	69.24	112.6
MRET_1008	RhoGEF domain protein	181.41	90.5
MRET_1009	BadF/BadG/BcrA/BcrD ATPase family	17.26	13.19
MRET_1010	tRNA (cytosine34-C5)-methyltransferase	54.7	31.26
MRET_1011	2-dehydropantoate 2-reductase	235.14	181.46
MRET_1012	uncharacterized protein	756.82	579.39
MRET_1013	uncharacterized protein	65.81	33.56
MRET_1014	conserved hypothetical protein	23.36	36.2
MRET_1015	uncharacterized protein	85.21	199.1
MRET_1016	peptide-methionine (S)-S-oxide reductase	1472.67	1319.02
MRET_1017	uncharacterized protein	24.37	54.15
MRET_1018	ubiquitin-conjugating enzyme E2 J1	8.74	25.92
MRET_1019	ER membrane protein	643.66	481.75
MRET_1020	fatty acid hydroxylase	89.28	151.26
MRET_1021	uncharacterized protein	25.44	57.42
MRET_1022	adenylyltransferase and sulfurtransferase	54.99	57.57
MRET_1023	Importin-11	54.54	69.59
MRET_1024	vacuolar carboxypeptidase	1121.27	939.77
MRET_1025	SNF2 family helicase ATPase	27.47	16.83
MRET_1026	alkylated DNA repair protein alkB homolog 6	34.09	17.84
MRET_1027	mitosis inhibitor protein kinase SWE1	53.3	86.51
MRET_1028	uncharacterized protein	97.4	213.26
MRET_1029	uncharacterized protein	300.03	424.42
MRET_1030	small subunit ribosomal protein S6e	138.15	629.32
MRET_1031	small subunit ribosomal protein S13e	192.33	981.01
MRET_1032	lipase	31.13	29.77
MRET_1033	NADPH-dependent medium chain alcohol dehydrogenase	126.28	140.29

MRET_1034	inositol-polyphosphate multikinase	2.32	2.69
MRET_1035	vacuolar protein sorting-associated protein 52	48.28	47.67
MRET_1036	protein regulator of cytokinesis 1	62.67	100.67
MRET_1037	protein NUD1	20.88	35.72
MRET_1038	riboflavin synthase	60.34	102
MRET_1039	bud site selection protein 31	36.5	50.81
MRET_1040	GTPase-activating protein SST2	72.5	79.24
MRET_1041	histone deacetylase 6	39.57	41.8
MRET_1042	uncharacterized protein	49.04	35.89
MRET_1043	uncharacterized protein	78.5	68.68
MRET_1044	uncharacterized protein	25.27	32.64
MRET_1045	26S proteasome regulatory subunit N11	154.1	153.18
MRET_1046	nuclear pore complex protein Nup160	34.23	25.22
MRET_1047	kinesin family member 18/19	17.3	32.34
MRET_1048	protein-S-isoprenylcysteine O-methyltransferase	13.45	22.72
MRET_1049	dihydroorotase	17.35	38.21
MRET_1050	zinc finger protein HUA1	130.25	100.26
MRET_1051	cactin protein	220.91	140
MRET_1052	anticodon-binding domain protein	55.44	122.62
MRET_1053	NADH dehydrogenase (ubiquinone) 1 alpha subcomplex subunit 2	107.23	116.44
MRET_1054	protein pelota	55.93	59.77
MRET_1055	uncharacterized protein	28.6	54.66
MRET_1056	uncharacterized protein	11.39	26.47
MRET_1057	uncharacterized protein	41.06	100.24
MRET_1058	Gly-Xaa carboxypeptidase	62.55	39.41
MRET_1059	Anion exchange family protein	36.95	68.88
MRET_1060	RNA polymerase Rpb4	22.58	54.21
MRET_1061	small subunit ribosomal protein S18	56.15	132.01
MRET_1062	thiamine-phosphate diphosphorylase/hydroxyethylthiazole kinase	118.5	105.98
MRET_1063	GINS complex subunit 3	97.22	132.08
MRET_1064	exocyst complex component 5	99.37	102.48
MRET_1065	uncharacterized protein	18.02	22.95
MRET_1066	peroxin-2	38.85	35.25
MRET_1067	magnesium-dependent phosphatase 1	513.22	146.69
MRET_1068	histone deacetylase 1/2	205.24	148.92
MRET_1069	general transcription factor 3C polypeptide 3 (transcription factor C subunit 4)	186.81	81.97
MRET_1070	uncharacterized protein	63.43	110.21

MRET_1071	18S rRNA (guanine1575-N7)-methyltransferase	29.55	56.02
MRET_1072	syntaxin 18	91.06	136.42
MRET_1073	pyruvate dehydrogenase kinase	26.59	49.24
MRET_1074	uncharacterized protein	51.58	67.22
MRET_1075	calcium calmodulin-dependent protein kinase	54.99	109.46
MRET_1076	L-threonylcarbamoyladenylate synthase	189.1	161.76
MRET_1077	cytochrome c oxidase assembly factor 6	29.52	105.01
MRET_1078	uncharacterized protein	19.18	53.79
MRET_1079	L-2-aminoadipate reductase	32.95	45.01
MRET_1080	cell morphogenesis protein (PAG1)	33.76	29.85
MRET_1081	poly(A) RNA binding protein involved in nuclear mRNA export	15.72	33.73
MRET_1082	phosphatidylinositol glycan, class F	49.24	39.2
MRET_1083	AHNAK nucleoprotein	20.23	27.94
MRET_1084	mitochondrial distribution and morphology protein 12	45.89	38.89
MRET_1085	uncharacterized protein	62.98	123.64
MRET_1086	large subunit ribosomal protein L29e	288.06	556.37
MRET_1087	FYVE zinc finger protein	63.36	41.1
MRET_1088	nucleoporin NUP82	114.26	79.55
MRET_1089	phosphatidylserine decarboxylase	510.95	232.63
MRET_1090	uncharacterized protein	1542.46	3133.36
MRET_1091	Rhodanese-like domain protein	52.69	67.11
MRET_1092	nucleolar protein 58	150.67	188.21
MRET_1093	DNA polymerase alpha subunit B	95.04	104.42
MRET_1094	general transcription factor 3C polypeptide 5 (transcription factor C subunit 1)	14.08	27.9
MRET_1095	Ran-binding protein 1	151.5	363.35
MRET_1096	p24 family protein beta-1	65.35	168.27
MRET_1097	uncharacterized protein	39.21	76.47
MRET_1098	Fe-S cluster assembly protein DRE2	539.84	530.56
MRET_1099	multidrug resistance protein fnx1	213.46	97.64
MRET_1100	serine/threonine-protein kinase RIM15	133.39	63.94
MRET_1101	uncharacterized protein	55.34	56.25
MRET_1102	phosphatidate cytidylyltransferase	34.46	129.16
MRET_1103	serine/threonine-protein kinase	58.51	34.73
MRET_1104	NADH dehydrogenase (ubiquinone) Fe-S protein 7	555.76	195.09
MRET_1105	type I protein arginine methyltransferase	69.97	117.45
MRET_1106	golgi SNAP receptor complex member 2	47.48	60.77
MRET_1107	zinc finger CCHC domain protein 9	65.57	54.36

MRET_1108	tRNA-specific adenosine deaminase 1	30.17	33.56
MRET_1109	NAD-dependent deacetylase sirtuin 2	107.11	180.74
MRET_1110	uncharacterized protein	351.08	206.85
MRET_1111	delta7-sterol 5-desaturase	113.16	308.12
MRET_1112	sorting nexin-3/12	49.91	85.35
MRET_1113	large subunit ribosomal protein L7e	162.95	598.57
MRET_1114	heat shock 70kDa protein 4	712.69	812.98
MRET_1115	conserved hypothetical protein	6966.72	3923.27
MRET_1116	conserved hypothetical protein	92.24	94.88
MRET_1117	transitional endoplasmic reticulum ATPase	1374.72	1149.33
MRET_1118	IKS protein kinase	127.95	124.11
MRET_1119	BRCA1-associated protein	57.49	65.33
MRET_1120	dolichyl-phosphate mannosyltransferase polypeptide 2, regulatory subunit	31.12	140.32
MRET_1121	uncharacterized protein	25.9	42.82
MRET_1122	La domain protein	23.45	46.43
MRET_1123	lysine methyltransferase	19.17	69.17
MRET_1124	uncharacterized protein	81.07	204.66
MRET_1125	HAT1-interacting factor 1	224.38	245.84
MRET_1126	adenylylsulfate kinase	114.49	181.18
MRET_1127	FH domain protein	23.82	41.23
MRET_1128	OHCU decarboxylase	40.67	64.67
MRET_1129	cytochrome-b5 reductase	146.02	84.15
MRET_1130	conserved oligomeric golgi complex subunit 4	116.47	75.25
MRET_1131	3-hydroxy acid dehydrogenase/malonic semialdehyde reductase	92.72	125.33
MRET_1132	spindle assembly associated Sfi1-like protein	34.73	46.24
MRET_1133	pre-rRNA-processing protein TSR2	26.84	40.17
MRET_1134	programmed cell death 6-interacting protein	138.99	44.21
MRET_1135	uncharacterized protein	126.7	64.75
MRET_1136	ubiquitin-conjugating enzyme E2 G2	193.9	228.78
MRET_1137	ribosome assembly protein 4	41.44	63.44
MRET_1138	essential protein, constituent of 66S pre-ribosomal particles	32.46	64.21
MRET_1139	DASH complex subunit DAD3	469.37	351.43
MRET_1140	protein of unknown function (DUF1769)	357.46	199.47
MRET_1141	NAD-dependent histone deacetylase SIR2	37.35	35.08
MRET_1142	R3H domain protein	235.56	113.91
MRET_1143	uncharacterized protein	16.06	25.7
MRET_1144	ubiquitin-activating enzyme E1 C	112.92	78.38

MRET_1145	deoxyhypusine synthase	19.6	43.15
MRET_1146	phosphatidylinositol glycan, class K	47.18	83.65
MRET_1147	V-type H+-transporting ATPase 21kDa proteolipid subunit	287.76	428.99
MRET_1148	oleate-activated transcription factor	27.32	54.93
MRET_1149	transcription elongation factor B, polypeptide 1	234.66	512.57
MRET_1150	protein STU1	4.24	33.65
MRET_1151	very-long-chain enoyl-CoA reductase	148.36	183.69
MRET_1152	N-lysine methyltransferase SETD6	15.2	34.12
MRET_1153	zinc finger protein, C2H2 type	25.66	32.3
MRET_1154	beta-glucan synthesis-associated protein KRE6	44.41	61.24
MRET_1155	beta-glucan synthesis-associated protein KRE6	14.19	36
MRET_1156	flap endonuclease-1	33.97	112.61
MRET_1157	uncharacterized protein	32.67	31
MRET_1158	glutamate-5-semialdehyde dehydrogenase	301.68	147.74
MRET_1159	acyl-CoA-dependent ceramide synthase	82.21	106.07
MRET_1160	riboflavin aldehyde-forming enzyme	153.3	124.41
MRET_1161	Rab geranylgeranyl transferase escort protein	35.47	64.11
MRET_1162	tripeptidyl-peptidase II	36.16	71.67
MRET_1163	SET and MYND domain protein	25.25	19.21
MRET_1164	G1 S-specific cyclin Pcl5	4418.67	4701.9
MRET_1165	RTA1 domain protein	157.96	412.85
MRET_1166	proliferating cell nuclear antigen	395.75	946.27
MRET_1167	magnesium transporter	16.84	41.56
MRET_1168	cell division control protein	58.31	188
MRET_1169	cytochrome c oxidase subunit 7c	164.68	169.42
MRET_1170	ribosome production factor 1	262.35	190.05
MRET_1171	nuclear pore complex protein Nup107	62.63	134.47
MRET_1172	transcription initiation factor TFIIB	371.73	282.03
MRET_1173	zinc finger protein	79.41	173.22
MRET_1174	histidine kinase	22.77	33.44
MRET_1175	uncharacterized protein	20.44	41.51
MRET_1176	small subunit ribosomal protein S7	46.39	92.21
MRET_1177	alkaline phosphatase D	68.24	92.66
MRET_1178	glutamine amidotransferase/cyclase	129.2	127.52
MRET_1179	secretory lipase	396.83	221.83
MRET_1180	uncharacterized protein	90.79	49.31
MRET_1181	1-phosphatidylinositol-4-phosphate 5-kinase	357.43	212.88

MRET_1182	DNA primase large subunit	89.07	68.84
MRET_1183	U3 small nucleolar RNA-associated protein 6	236.58	150.41
MRET_1184	mitochondrial ATPase complex subunit ATP10	144.22	191.15
MRET_1185	Ras-related protein Rab-8A	311.38	550.99
MRET_1186	uncharacterized protein	29.85	50.78
MRET_1187	uncharacterized protein	642.76	697.89
MRET_1188	vacuolar protein sorting-associated protein 11	39.71	32.14
MRET_1189	long-chain acyl-CoA synthetase	92.28	65.9
MRET_1190	squalene monooxygenase	41.87	102.06
MRET_1191	U3 small nucleolar RNA-associated protein 23	205.07	226.89
MRET_1192	leucine-carboxy methyltransferase	95.35	60.62
MRET_1193	solute carrier family 25 (mitochondrial carnitine/acylcarnitine transporter), member 20/29	174.72	94.24
MRET_1194	uncharacterized protein	336.21	167.59
MRET_1195	nuclear pore complex protein Nup155	51.17	35.35
MRET_1196	elongation factor Ts	98.35	95.05
MRET_1197	Ras-related protein Rab-7A	529.59	452.76
MRET_1198	Ras-related GTP-binding protein C/D	361.92	194.97
MRET_1199	translation machinery-associated protein 22	291.51	195.34
MRET_1200	COMPASS component BRE2	31.87	31.62
MRET_1201	maintenance of mitochondrial morphology protein 1	63.66	38.02
MRET_1202	DnaJ chaperone Caj1	63.69	45.23
MRET_1203	NADPH-dependent beta-ketoacyl reductase	517.92	314.92
MRET_1204	complex 1 protein (LYR family)	22.08	32.23
MRET_1205	zinc finger protein, C2H2 type	204.89	177.44
MRET_1206	phosphopantothenate---cysteine ligase (ATP)	93.24	88.57
MRET_1207	DNA methyltransferase 1-associated protein 1	30.29	36.97
MRET_1208	uncharacterized protein	21.51	24.99
MRET_1209	galactokinase	227	173.15
MRET_1210	GINS complex subunit 4	85.16	91.37
MRET_1211	putative DNA replication factor C complex subunit Ctf8	129.13	74.28
MRET_1212	GATA zinc finger	93.66	46.64
MRET_1213	transcription initiation factor TFIIA small subunit	135.59	252.34
MRET_1214	centrin-3	126.7	211.08
MRET_1215	uncharacterized protein	22.66	34.55
MRET_1216	mitochondrial export protein SOM1	85.68	98.34
MRET_1217	adenosylhomocysteinase	114.14	193.4
MRET_1218	Xaa-Pro aminopeptidase	240.43	394.24

MRET_1219	serine/threonine-protein kinase 24/25/MST4	24.45	27.62
MRET_1220	uncharacterized protein	541.4	251.75
MRET_1221	uncharacterized protein	344.15	318.23
MRET_1222	uncharacterized protein	109.89	128.17
MRET_1223	N-acetyl-gamma-glutamyl-phosphate reductase/acetylglutamate kinase	45.67	78.37
MRET_1224	ER-derived vesicles protein	51.56	92.96
MRET_1225	phosphatidylinositol transfer protein	1013.18	413.3
MRET_1226	serine/threonine-protein phosphatase 2A regulatory subunit A	129.06	114.87
MRET_1227	DNA polymerase zeta	75.47	42.36
MRET_1228	phosphatidylethanolamine N-methyltransferase	36.73	62.91
MRET_1229	2,3-bisphosphoglycerate-dependent phosphoglycerate mutase	192.59	245.96
MRET_1230	zinc finger protein, GATA type	45.38	120.3
MRET_1231	protein of unknown function (DUF2841)	2.7	13.54
MRET_1232	ubiquitin-conjugating enzyme E2 N	155.55	164.5
MRET_1233	Wiskott-Aldrich syndrome protein	43.31	38.03
MRET_1234	elongation factor 1-alpha	1299.6	1982.16
MRET_1235	RAT1-interacting protein	25.74	36.86
MRET_1236	solute carrier family 30 (zinc transporter), member 1	1163.45	735.31
MRET_1237	uncharacterized protein	38.56	45.36
MRET_1238	conserved hypothetical protein	10.58	17.08
MRET_1239	transcription initiation factor TFIID subunit 7	113.47	90.25
MRET_1240	DNA-directed RNA polymerase III subunit RPC5	350.36	270.9
MRET_1241	nucleoside-diphosphate kinase	468.7	361.18
MRET_1242	exosome complex exonuclease DIS3/RRP44	330.85	80.15
MRET_1243	sulfite reductase (NADPH) hemoprotein beta-component	323.48	157.66
MRET_1244	mannose-1-phosphate guanylyltransferase	43	84.08
MRET_1245	large subunit ribosomal protein L36	59.55	68.37
MRET_1246	Urb2/Npa2 family protein	4.48	15.79
MRET_1247	transporter	0	50.89
MRET_1248	transporter	96.45	113.32
MRET_1249	SMR domain protein	77.81	68.83
MRET_1250	mitotic cell cycle regulation protein	214.85	146.28
MRET_1251	small subunit ribosomal protein S17	77.83	82.01
MRET_1252	uncharacterized protein	27.02	76.33
MRET_1253	serine/threonine-protein kinase 16	86.15	35.18
MRET_1254	subunit of COMPASS (Set1C)	45.51	26.45
MRET_1255	DUF543 domain protein	165.9	180.86

MRET_1256	putative ATPase	95.5	65.34
MRET_1257	E3 SUMO-protein ligase PIAS1	29.91	35.99
MRET_1258	SAGA complex subunit spt20	152.02	206.8
MRET_1259	conserved hypothetical protein	251.05	227.94
MRET_1260	chromosome transmission fidelity protein 4	17.53	34.16
MRET_1261	phosphoserine aminotransferase	263.65	184.11
MRET_1262	transcription initiation factor TFIIF subunit beta	62.19	71.77
MRET_1263	zinc finger protein (RING finger)	84.42	58.42
MRET_1264	heat shock 70kDa protein 1/2/6/8	3323.97	4615.66
MRET_1265	microtubule binding protein HOOK3	67.37	100.3
MRET_1266	uncharacterized protein	31.77	30.36
MRET_1267	E3 ubiquitin-protein ligase HUWE1	252.12	148.25
MRET_1268	cullin 4	27.19	27.88
MRET_1269	biotin---protein ligase	62.76	46.59
MRET_1270	4-nitrophenyl phosphatase	49.02	61.94
MRET_1271	ion transport protein	210.78	311.66
MRET_1272	casein kinase I	712.97	838.87
MRET_1273	transporter	21.71	31.05
MRET_1274	U3 small nucleolar RNA-associated protein 11	18.39	69.61
MRET_1275	conserved hypothetical protein	18.8	64.71
MRET_1276	26S proteasome non-ATPase regulatory subunit 9	28.36	63.12
MRET_1277	uncharacterized protein	463.83	251.49
MRET_1278	S-(hydroxymethyl)glutathione dehydrogenase/alcohol dehydrogenase	1143.47	687.03
MRET_1279	carbohydrate kinase	41.74	58.86
MRET_1280	ceramide glucosyltransferase	37.85	47.17
MRET_1281	ubiquitin-conjugating enzyme E2 I	54.6	156.33
MRET_1282	tropomyosin, fungi type	98.5	261.95
MRET_1283	serine/threonine-protein kinase	280.42	166.92
MRET_1284	PHD finger domain protein	19.75	44.57
MRET_1285	uncharacterized protein	43.62	93.06
MRET_1286	negative regulator of eIF2 kinase Gcn2p	186.73	216.62
MRET_1287	FUN14 family protein	1310.68	582.57
MRET_1288	fungal protein of unknown function (DUF2015)	364.39	231.27
MRET_1289	trafficking protein particle complex subunit 12	32.95	41.29
MRET_1290	histone acetyltransferase 1	37.94	50.86
MRET_1291	type 2A phosphatase activator TIP41	163.54	165.12
MRET_1292	cell cycle checkpoint control protein RAD9A	48.26	79.88

MRET_1293	p21-activated kinase 1	28.7	80.71
MRET_1294	cryptococcal mannosyltransferase 1	30.05	26.62
MRET_1295	conserved oligomeric golgi complex subunit 2	107.13	52.58
MRET_1296	UDP-N-acetylglucosamine/UDP-N-acetylgalactosamine diphosphorylase	35.08	63.94
MRET_1297	uncharacterized protein	30.87	62.53
MRET_1298	heat shock 70kDa protein 5	510.56	726.7
MRET_1299	peroxin-10	65.02	104.15
MRET_1300	cysteine-rich secretory protein	2358.57	2077.02
MRET_1301	sphinganine C4-monooxygenase	625.2	308.25
MRET_1302	osomolarity two-component system, response regulator SSK1	38.98	42.06
MRET_1303	nuclear control of ATPase protein 2	40.18	55.16
MRET_1304	glycylpeptide N-tetradecanoyltransferase	69.16	106.35
MRET_1305	pre-mRNA-splicing factor RBM22/SLT11	100.65	106.2
MRET_1306	LYR motif containing 7	15.98	63.05
MRET_1307	E3 ubiquitin-protein ligase NEDD4	460.63	419.51
MRET_1308	integral membrane protein	16.86	79.64
MRET_1309	histone deacetylase	32.66	109.52
MRET_1310	chitin deacetylase	44.1	295.46
MRET_1311	LETM1 and EF-hand domain protein 1, mitochondrial	123.56	189.86
MRET_1312	ribosomal RNA-processing protein 12	33.75	42.41
MRET_1313	putative ferric reductase with similarity to Fre2p	183.5	379.84
MRET_1314	phosphatidylinositol glycan, class T	354.03	647.68
MRET_1315	protein ROT1	33.95	64.05
MRET_1316	uncharacterized protein	15.2	24.12
MRET_1317	coatomer subunit alpha	90.01	116.24
MRET_1318	mitochondrial ribosomal protein subunit	34.01	81.89
MRET_1319	6,7-dimethyl-8-ribityllumazine synthase	69.75	172.03
MRET_1320	3-hydroxyisobutyryl-CoA hydrolase	130.67	116.96
MRET_1321	asparaginyl-tRNA synthetase	101.18	119.83
MRET_1322	cell division cycle protein	47.5	47.56
MRET_1323	nuclear cap-binding protein subunit 2	58.57	91.89
MRET_1324	nascent polypeptide-associated complex subunit beta	31.52	89.69
MRET_1325	F-type H+-transporting ATP synthase subunit e	351.12	443.32
MRET_1326	acetyl-CoA synthetase	87.56	159.56
MRET_1327	replication factor A3	77.44	205.45
MRET_1328	sensitive to high expression protein 9, mitochondrial	33.37	52.42
MRET_1329	elongation factor 3	73.77	112.78

MRET_1330	DNA excision repair protein ERCC-2	13.61	32.59
MRET_1331	uncharacterized protein	24	30.35
MRET_1332	Rab guanine nucleotide exchange factor SEC2	41.3	52.23
MRET_1333	uncharacterized protein	440.74	372.46
MRET_1334	5-oxoprolinase (ATP-hydrolysing)	354.34	369.37
MRET_1335	mitogen-activated protein kinase kinase kinase	340.06	219.77
MRET_1336	manganese-transporting P-type ATPase	34.88	67.86
MRET_1337	solute carrier family 25 (mitochondrial dicarboxylate transporter), member 10	15.58	35.61
MRET_1338	uncharacterized protein	87.07	61.99
MRET_1339	Rab6A-GEF complex partner protein 1	13.46	20.79
MRET_1340	mitogen-activated protein kinase kinase kinase	36.36	40.57
MRET_1341	uncharacterized protein	50.61	68.38
MRET_1342	synaptobrevin homolog YKT6	193.71	381.41
MRET_1343	Vps51/Vps67 family protein	308.34	150.12
MRET_1344	PHD finger domain protein	34.51	43.28
MRET_1345	uncharacterized protein	107.19	98.58
MRET_1346	uncharacterized protein	13.21	83.01
MRET_1347	2-oxoglutarate dehydrogenase E2 component (dihydrolipoamide succinyltransferase)	1188.23	1063.41
MRET_1348	solute carrier family 25, member 38	18.72	31.21
MRET_1349	RasGEF	6.95	15.58
MRET_1350	mitogen-activated protein kinase kinase	28.87	62.36
MRET_1351	NADH dehydrogenase (ubiquinone) 1 alpha subcomplex subunit 6	64.16	76.14
MRET_1352	origin recognition complex subunit 2	24.83	25.46
MRET_1353	Integral ER membrane protein	364.05	375.76
MRET_1354	uncharacterized protein	113.63	110.08
MRET_1355	V-type H+-transporting ATPase subunit B	185.6	262.8
MRET_1356	peptidyl-tRNA hydrolase ICT1	67.42	94.95
MRET_1357	large subunit ribosomal protein L28	30.11	100.59
MRET_1358	U3 small nucleolar ribonucleoprotein protein IMP4	324.42	158.36
MRET_1359	glycerol-3-phosphate dehydrogenase	513.44	202.34
MRET_1360	cell cycle checkpoint protein	38.95	33.86
MRET_1361	importin-7	16.71	24.45
MRET_1362	succinate dehydrogenase assembly factor 2	11.78	35.68
MRET_1363	large subunit ribosomal protein L37Ae	65.54	366.7
MRET_1364	DnaJ homolog subfamily C member 19	526.01	348.2
MRET_1365	uncharacterized protein	8.32	6.67
MRET_1366	WD domain, G-beta repeat protein	34.12	30.09

MRET_1367	protein transport protein SEC23	119.42	157.12
MRET_1368	DEP domain protein 5	215.93	143.82
MRET_1369	Aur1-inositol phosphorylceramide synthase	31.48	51.31
MRET_1370	uncharacterized protein	257.68	289.74
MRET_1371	mitochondrial inner membrane protease subunit 1	20.84	35.15
MRET_1372	solute carrier family 25 (mitochondrial folate transporter), member 32	30.36	73.76
MRET_1373	PIN domain protein	26.67	62.74
MRET_1374	ubiquitin-like protein 5	156.86	193.23
MRET_1375	translation initiation factor 4E	582.48	267.23
MRET_1376	uncharacterized protein	14.74	16.87
MRET_1377	histidinol-phosphatase (PHP family)	98.59	76.2
MRET_1378	succinate dehydrogenase (ubiquinone) iron-sulfur subunit	269.8	76.09
MRET_1379	DNA ligase 1	20.85	26.18
MRET_1380	mitochondrial inner membrane protein subunit 18	50.46	64.44
MRET_1381	uncharacterized protein	14.13	19.42
MRET_1382	Spo7-like protein	287.77	185.41
MRET_1383	recyclin-1	24.79	29.14
MRET_1384	cell division protein	159.34	107.34
MRET_1385	ribosome biogenesis protein Sqt1	415.79	237.36
MRET_1386	ribose 5-phosphate isomerase A	89.15	73.64
MRET_1387	25S rRNA (adenine2142-N1)-methyltransferase	38.27	45.98
MRET_1388	glutaredoxin 3	3293.49	2176.85
MRET_1389	autophagy-related protein 18	147.21	349.41
MRET_1390	ribosomal RNA-processing protein 9	27.25	45.4
MRET_1391	telomerase activating protein Est1	61.4	39.97
MRET_1392	COP9 signalosome complex subunit 1	84.39	44.79
MRET_1393	COP9 signalosome complex subunit 4	87.18	57.34
MRET_1394	Rwd-domain protein	73.95	128.81
MRET_1395	molecular chaperone DnaK	2405.91	2020.29
MRET_1396	PX domain protein	105.43	142.69
MRET_1397	mitochondrial import receptor subunit TOM20	150.24	190.19
MRET_1398	choline/ethanolamine kinase	13.26	29.34
MRET_1399	uncharacterized protein	79.02	53.83
MRET_1400	poly(ADP-ribose) polymerase	319.27	525.55
MRET_1401	smad nuclear-interacting protein 1	259.59	233.01
MRET_1402	tRNA ligase	35.78	48.51
MRET_1403	multicopper oxidase	71.64	52.88

MRET_1404	multicopper oxidase	44.66	53.41
MRET_1405	phospholipase C	48.85	98.49
MRET_1406	phospholipase C	21.33	45.79
MRET_1407	phospholipase C	155.7	123.21
MRET_1408	phospholipase C	69	79.23
MRET_1409	uncharacterized protein	110.46	161.79
MRET_1410	histone-lysine N-methyltransferase SETD1	69.34	205.94
MRET_1411	DDHD domain protein	81.41	100.99
MRET_1412	fluoride exporter	51.02	75.57
MRET_1413	uncharacterized protein	19.74	36.14
MRET_1414	cell wall protein	17.27	11.49
MRET_1415	uncharacterized protein	26.89	26.11
MRET_1416	small subunit ribosomal protein S26e	371.08	882.48
MRET_1417	cleavage and polyadenylation specificity factor subunit 1	40.37	58.25
MRET_1418	WW domain-binding protein 4	18.83	45.16
MRET_1419	transcription factor	60.53	74.92
MRET_1420	tubulin alpha	76.81	110.55
MRET_1421	solute carrier family 25 (mitochondrial phosphate transporter), member 23/24/25/41	91.19	158.71
MRET_1422	acyl-CoA oxidase	1033.96	733.36
MRET_1423	lactoylglutathione lyase	75.54	172.63
MRET_1424	bZIP transcription factor	104.01	119.87
MRET_1425	coatomer subunit gamma	63.25	79.13
MRET_1426	small subunit ribosomal protein S28e	82.04	411.43
MRET_1427	peroxisomal membrane anchor protein PEX14p	67.97	78.34
MRET_1428	DNA helicase INO80	77.9	55.44
MRET_1429	splicing factor 3B subunit 5	174.25	171.89
MRET_1430	3-oxo-5-alpha-steroid 4-dehydrogenase 1	120.96	99.85
MRET_1431	CTP synthase	46.85	74.07
MRET_1432	uncharacterized protein	110.76	287.47
MRET_1433	zinc finger protein	15.65	23.01
MRET_1434	uncharacterized protein	28.93	49.62
MRET_1435	pre-mRNA-splicing factor CWC26	18.27	35.15
MRET_1436	transcription initiation factor TFIIIB component B''	69.37	104.14
MRET_1437	uncharacterized protein	235.35	260.94
MRET_1438	translation machinery-associated protein 16	69.81	137.46
MRET_1439	conserved hypothetical protein	29.86	19.05
MRET_1440	inositol-pentakisphosphate 2-kinase	6	10.16

MRET_1441	SRP40, C-terminal domain protein	20.88	32.05
MRET_1442	tRNASer (uridine44-2'-O)-methyltransferase	13.65	19.72
MRET_1443	splicing factor 3B subunit 3	55.04	105.32
MRET_1444	RhoGEF domain protein	20.21	42.05
MRET_1445	oxidation resistance protein 1	34.43	46.89
MRET_1446	uncharacterized protein	60.75	51.55
MRET_1447	F-box domain protein	62.4	77.9
MRET_1448	tubulin beta	561.68	476.46
MRET_1449	aspartyl-tRNA synthetase	50.54	85.72
MRET_1450	acylglycerol lipase	85.73	119.58
MRET_1451	carnitine O-acetyltransferase	184.12	156.48
MRET_1452	uncharacterized protein	269.35	329.3
MRET_1453	protein kinase	68.51	64.4
MRET_1454	heparinase II III family protein	221.1	226.74
MRET_1455	AMP-binding enzyme	19.96	28.91
MRET_1456	DNA-directed RNA polymerases I, II, and III subunit RPABC2	307.03	670.95
MRET_1457	zinc finger protein, C2H2 type	225.18	198.57
MRET_1458	DNA mismatch repair protein MSH3	5.22	12.63
MRET_1459	geranylgeranyl transferase type-2 subunit beta	158.02	181.79
MRET_1460	AHNAK nucleoprotein	25	33.29
MRET_1461	pre-rRNA-processing protein TSR3	137.35	116.28
MRET_1462	isocitrate lyase	620.96	392.67
MRET_1463	integral membrane protein (Ptm1)	52.46	148.62
MRET_1464	SEL1 domain protein	309.08	199.99
MRET_1465	peroxisomal biogenesis factor	349.56	489.23
MRET_1466	translation initiation factor 3 subunit B	88.05	179.16
MRET_1467	AdoMet-dependent methyltransferase SPB1	61.48	107.24
MRET_1468	large subunit ribosomal protein L22e	160.63	987.68
MRET_1469	H/ACA ribonucleoprotein complex subunit 4	781.43	720.3
MRET_1470	cytochrome c oxidase subunit 11	150.02	311.2
MRET_1471	small subunit ribosomal protein S5e	602.71	1230.58
MRET_1472	transducin (beta)-like 1	104.41	113.33
MRET_1473	rhomboid family protein	319.11	161.52
MRET_1474	PPR repeat containing protein	26.45	46.63
MRET_1475	mitochondrial 54S ribosomal protein YmL8	23.75	48.88
MRET_1476	NADH dehydrogenase (ubiquinone) 1 alpha/beta subcomplex 1	1421.6	945.26
MRET_1477	serine/threonine-protein kinase SCH9	345.09	519.26

MRET_1478	N-alpha-acetyltransferase 15/16, NatA auxiliary subunit	18.03	34.59
MRET_1479	elongation factor 2	148.93	305.42
MRET_1480	prefoldin subunit 4	20.66	45.46
MRET_1481	superkiller protein 3	18.18	23.19
MRET_1482	UDP-N-acetylglucosamine--dolichyl-phosphate N-acetylglucosaminephosphotransferase	22.36	33.79
MRET_1483	inosine triphosphate pyrophosphatase	36.85	72.19
MRET_1484	NAD dependent epimerase dehydratase family protein	876.25	322.07
MRET_1485	phosphatidylinositol 4-phosphatase	114.19	322.69
MRET_1486	pre-mRNA-processing factor 19	78.87	156.59
MRET_1487	anthranilate synthase/indole-3-glycerol phosphate synthase/phosphoribosylanthranilate isomerase	108.85	224.91
MRET_1488	actin-related protein 9	67.32	92.75
MRET_1489	phosphoacetylglucosamine mutase	209.38	187.46
MRET_1490	mannose-P-dolichol utilization defect 1	106.43	124.33
MRET_1491	translation initiation factor 3 subunit H	75.72	181.62
MRET_1492	zinc finger protein, C2H2 type	18.23	33.55
MRET_1493	RNA polymerase II subunit A C-terminal domain phosphatase SSU72	194.05	260.45
MRET_1494	recombining binding protein suppressor of hairless	108.74	83.14
MRET_1495	ribosomal biogenesis protein LAS1	65.08	94.69
MRET_1496	conserved hypothetical protein	1979.36	988.17
MRET_1497	phosphoribosylaminoimidazole-succinocarboxamide synthase	57.54	109.29
MRET_1498	kinesin family member 11	18.24	53.82
MRET_1499	COMPASS component SWD3	35	59.92
MRET_1500	DNA-directed RNA polymerase II subunit RPB4	108.09	144.12
MRET_1501	mitochondrial serine protease	52.46	123.44
MRET_1502	kynurenine aminotransferase	622.51	515.67
MRET_1503	sorting nexin	334.23	423.41
MRET_1504	serine/threonine-protein phosphatase	223.13	231.33
MRET_1505	translation initiation factor 5B	131.87	144.47
MRET_1506	carbamoyl-phosphate synthase large subunit	76.68	28.33
MRET_1507	alpha/beta-hydrolase lipase	37.88	58.25
MRET_1508	uncharacterized protein	475.91	370.77
MRET_1509	complement component 1 Q subcomponent-binding protein, mitochondrial	124.42	150.9
MRET_1510	disulfide isomerase	194.2	248.58
MRET_1511	rapamycin-insensitive companion of mTOR	30.96	47.16
MRET_1512	cytochrome c oxidase assembly factor 1	72.43	166.12
MRET_1513	mannoprotein MP88	2080.55	1306.28
MRET_1514	small subunit ribosomal protein S16	111.89	158.49

MRET_1515	AHNAK nucleoprotein	159.33	111.66
MRET_1516	conserved hypothetical protein	27.93	44.57
MRET_1517	membrane magnesium transporter	134.26	257.19
MRET_1518	actin beta/gamma 1	2269.4	1819.97
MRET_1519	nucleolar protein 56	80.71	178.67
MRET_1520	20S proteasome subunit alpha 1	139.82	217.67
MRET_1521	RNA polymerase-associated protein CTR9	39.64	56.91
MRET_1522	5'-phosphate synthase pdxT subunit	98.75	97.83
MRET_1523	MT-A70 family protein	34.93	35.63
MRET_1524	T-complex protein 1 subunit beta	129.86	243.03
MRET_1525	cytochrome c oxidase subunit 4	351.97	299.24
MRET_1526	O-acetylhomoserine/O-acetylserine sulfhydrylase	279.87	478.24
MRET_1527	cystathionine beta-lyase	73.98	63.71
MRET_1528	RNA 3'-terminal phosphate cyclase-like protein	24.31	42.67
MRET_1529	conserved hypothetical protein	223.93	203.1
MRET_1530	microfibrillar-associated protein 1	168.63	118.87
MRET_1531	ADP-ribosylation factor	72.11	67.81
MRET_1532	helicase SWR1	186.96	124.28
MRET_1533	F-type H+-transporting ATPase subunit epsilon	238.09	334.99
MRET_1534	protein of unknown function (DUF423)	1458.25	1234.35
MRET_1535	uncharacterized protein	26.89	48.15
MRET_1536	TBC1 domain family, member 13	43.25	40.5
MRET_1537	LEM3 CDC50 family protein	26.56	65.12
MRET_1538	GTP-binding protein 1	570.91	332.28
MRET_1539	tyrosine-protein phosphatase SIW14	164.66	368.14
MRET_1540	oxysterol-binding protein-related protein 8	212.33	485.76
MRET_1541	transcriptional co-repressor	20.89	43.65
MRET_1542	pyridoxal 5'-phosphate synthase pdxS subunit	158.1	225.91
MRET_1543	ubiquinol-cytochrome c reductase iron-sulfur subunit	473.57	628.3
MRET_1544	FACT complex subunit SPT16	83.47	232.49
MRET_1545	GABA(A) receptor-associated protein	79.23	301.88
MRET_1546	serine/threonine-protein phosphatase 4 regulatory subunit 1	29.2	56.78
MRET_1547	oligopeptide transporter	43.79	95.71
MRET_1548	phosphodiesterase	712.63	618.01
MRET_1549	glutamate synthase (NADPH/NADH)	98.06	72.65
MRET_1550	U4/U6 small nuclear ribonucleoprotein PRP31	43.69	74.02
MRET_1551	adenylosuccinate synthase	93.41	127.17

MRET_1552	succinate dehydrogenase (ubiquinone) membrane anchor subunit	139.39	100.23
MRET_1553	DNA-directed RNA polymerase II subunit RPB9	148	465.75
MRET_1554	pyruvate dehydrogenase E1 component alpha subunit	739.04	547.25
MRET_1555	protein HPT1	53.44	73.28
MRET_1556	DUF962 domain protein	146.65	227.12
MRET_1557	trafficking protein particle complex subunit 13	16.96	18
MRET_1558	conserved hypothetical protein	40.18	46.26
MRET_1559	E3 ubiquitin-protein ligase ZNF598	40.79	62.14
MRET_1560	DASH complex subunit DAD1	66.33	78.84
MRET_1561	tRNA (guanine10-N2)-methyltransferase	159.09	172.51
MRET_1562	ATP dependent DNA ligase domain protein	42.58	44.49
MRET_1563	uncharacterized protein	20.91	25.91
MRET_1564	V-type H+-transporting ATPase subunit H	92.85	95.61
MRET_1565	large subunit ribosomal protein L46	171.37	152.66
MRET_1566	5'-AMP-activated protein kinase, regulatory gamma subunit	60.46	90.36
MRET_1567	cytochrome b pre-mRNA-processing protein 3	1027.85	458.79
MRET_1568	phosphoribosylformimino-5-aminoimidazole carboxamide ribotide isomerase	362.98	485.56
MRET_1569	nuclear pore complex protein Nup93	72.61	150.34
MRET_1570	serine/threonine-protein kinase SRPK3	429.14	305.31
MRET_1571	ubiquitin-conjugating enzyme	83.74	290.5
MRET_1572	uncharacterized protein	32.83	73.64
MRET_1573	6-phosphogluconolactonase	68.11	89.78
MRET_1574	threonyl-tRNA synthetase	32.64	119.97
MRET_1575	component of the SMC5-SMC6 complex	60.65	212.11
MRET_1576	exocyst complex component 6	47.32	80.77
MRET_1577	ATP-dependent RNA helicase DDX41	30.82	71.71
MRET_1578	ADP-ribosylation factor 6	45.81	73.84
MRET_1579	dynactin 1	33.72	42.38
MRET_1580	ATP-dependent Lon protease	52.9	42.69
MRET_1581	glutamyl-tRNA synthetase	47	43.72
MRET_1582	Ras-induced vulval development antagonist	86.67	52.54
MRET_1583	translation initiation factor 5	67.7	137.91
MRET_1584	trafficking protein particle complex subunit 5	49.78	88.51
MRET_1585	serine/threonine-protein kinase/endoribonuclease IRE1	19.72	33.67
MRET_1586	arsenite/tail-anchored protein-transporting ATPase	187.2	227.27
MRET_1587	N-terminal acetyltransferase B complex catalytic subunit	45.5	65.48
MRET_1588	UV radiation resistance-associated protein	12.38	39.26

MRET_1589	cell division control protein 45	18.25	56.65
MRET_1590	ATP-dependent helicase STH1/SNF2	1201.75	646.46
MRET_1591	universal stress protein	1143.44	1287.65
MRET_1592	large subunit ribosomal protein L3e	100.16	423.54
MRET_1593	PHD finger and BAH domain protein (Snt2)	23.83	29.85
MRET_1594	2OG-Fe(II) oxygenase superfamily	11.13	49.2
MRET_1595	MFS sugar transporter	25.09	27.59
MRET_1596	cyclin	198.48	211.61
MRET_1597	zinc finger protein	4.62	9.15
MRET_1598	transporter	52.6	89.17
MRET_1599	splicing factor 3B subunit 4	18.4	39.13
MRET_1600	Mn2 homeostasis protein (Per1)	30.35	85.35
MRET_1601	translation initiation factor 3 subunit J	43.27	120.18
MRET_1602	isoleucyl-tRNA synthetase	65.64	37.71
MRET_1603	mRNA (guanine-N7-)-methyltransferase	15.26	25.92
MRET_1604	N-alpha-acetyltransferase 35, NatC auxiliary subunit	11.32	10.04
MRET_1605	phosphatidylinositol glycan, class U	15.87	44.28
MRET_1606	calcium binding protein 39	17	50.06
MRET_1607	AP-1 complex subunit gamma-1	25.55	66.69
MRET_1608	MFS family protein	84.83	76.03
MRET_1609	regulator of nonsense transcripts 1	22.34	43.49
MRET_1610	molybdopterin binding domain protein	2524.71	1738.06
MRET_1611	SH3 domain YSC84-like protein 1	214.16	268.61
MRET_1612	eukaryotic translation initiation factor 2-alpha kinase 4	85.29	101.17
MRET_1613	type IV protein arginine methyltransferase	56.47	108.1
MRET_1614	nucleoporin p58/p45	52.69	139.72
MRET_1615	ATP-binding cassette, subfamily B (MDR/TAP), member 1	71.01	72.06
MRET_1616	uncharacterized protein	35.32	54.25
MRET_1617	uncharacterized protein	267.14	135.59
MRET_1618	cathepsin D	9.35	24.63
MRET_1619	uncharacterized protein	55.3	47.79
MRET_1620	electron transfer flavoprotein alpha subunit	434.97	248.79
MRET_1621	NAD-dependent histone deacetylase SIR2	24.86	42.93
MRET_1622	Rdx family protein	159.76	104.64
MRET_1623	uncharacterized protein	563.34	350.92
MRET_1624	transcription initiation factor TFIID complex subunit 8	51.74	157.32
MRET_1625	Cdc25 family phosphatase	80.44	102.96

MRET_1626	adiponectin receptor	25.87	28.4
MRET_1627	protein phosphatase PTC2/3	61.69	250.26
MRET_1628	translation initiation factor 3 subunit I	66.18	282.94
MRET_1629	pachytene checkpoint protein 2	20.61	59.95
MRET_1630	non-canonical poly(A) RNA polymerase PAPD5/7	54.4	45.95
MRET_1631	ATP-dependent RNA helicase DDX46/PRP5	119.74	73.16
MRET_1632	uncharacterized protein	54.96	66.29
MRET_1633	protein KTI12	81.58	115.19
MRET_1634	bZIP transcription factor	468.44	327.76
MRET_1635	protein SSH4	132.04	132.65
MRET_1636	serine/threonine-protein kinase 24/25/MST4	30.88	37.64
MRET_1637	uncharacterized protein	118.63	81.34
MRET_1638	scaffold protein involved in the formation of early endocytic sites	96.26	74.84
MRET_1639	oxidoreductase	25.88	67.42
MRET_1640	20S proteasome subunit beta 2	69.49	181.54
MRET_1641	SH3-binding, glutamic acid-rich protein	10.95	46.44
MRET_1642	splicing factor 3B subunit 2	49.1	86.98
MRET_1643	translation initiation factor 3 subunit A	30.31	60.03
MRET_1644	solute carrier family 41	15.76	33.4
MRET_1645	mitochondrial chaperone BCS1	24.64	61.12
MRET_1646	voltage-dependent calcium channel	6.31	16.64
MRET_1647	tubulin gamma	26.83	220.85
MRET_1648	rRNA 2'-O-methyltransferase fibrillarin	649.92	668.67
MRET_1649	cytochrome-b5 reductase	664.43	546.07
MRET_1650	predicted ATPase of the ABC class	73.22	74.09
MRET_1651	chitin synthase	29.04	45.69
MRET_1652	uncharacterized protein	1106.44	491.19
MRET_1653	solute carrier family 35, member F5	147.53	182.37
MRET_1654	chromatin structure-remodeling complex subunit RSC1/2	20.95	37.76
MRET_1655	casein kinase II subunit alpha	47.72	138.32
MRET_1656	tryptophanyl-tRNA synthetase	59.38	105.86
MRET_1657	U6 snRNA-associated Sm-like protein LSm2	70.57	137.04
MRET_1658	avl9 protein	19.3	49.04
MRET_1659	uncharacterized protein	43.32	98.79
MRET_1660	DNA-directed RNA polymerases I, II, and III subunit RPABC1	162.05	458.79
MRET_1661	KH domain protein	27.94	33.51
MRET_1662	RhoGAP	11.93	14.11

MRET_1663	protein involved in microtubule-related processes	50.29	73.13
MRET_1664	DUF6 domain protein	19.23	49.16
MRET_1665	tRNA (uracil-5-)-methyltransferase	25.46	79.95
MRET_1666	uncharacterized protein	152.96	179.21
MRET_1667	uncharacterized protein	76.43	153.87
MRET_1668	GINS complex subunit 2	38.09	70.28
MRET_1669	golgi membrane protein involved in vesicular trafficking and spindle migration	151.73	181.21
MRET_1670	cytochrome c heme-lyase	241.08	312.21
MRET_1671	DNA-directed RNA polymerase III subunit RPC3	180.56	294.38
MRET_1672	cell wall protein	17.11	30.29
MRET_1673	heat shock transcription factor	58.71	82.05
MRET_1674	transcription initiation factor TFIID TATA-box-binding protein	1420.8	1227.88
MRET_1675	YidC/Oxa1 family membrane protein insertase	91.88	156.76
MRET_1676	auxiliary protein of DNA polymerase delta	14.63	41.16
MRET_1677	chromatin structure-remodeling complex subunit RSC9	55.28	82.36
MRET_1678	sorting nexin-1/2	139.67	224.3
MRET_1679	S-adenosylmethionine synthetase	234.8	207.48
MRET_1680	uncharacterized protein	8.17	43.22
MRET_1681	anaphase-promoting complex subunit 2	8.78	19.11
MRET_1682	Sec7 domain protein	52.54	106.67
MRET_1683	protein STE50	142.12	91.99
MRET_1684	integral membrane protein	35.73	98.04
MRET_1685	uncharacterized protein	59.18	91.14
MRET_1686	mitochondrial intermembrane space import and assembly protein 40	2182.72	1368.25
MRET_1687	peroxin-7	224	229.71
MRET_1688	MFS phosphate transporter	60.16	71.54
MRET_1689	cation-transporting P-type ATPase 13A3/4/5	38.27	60.75
MRET_1690	uncharacterized protein	20.64	45.88
MRET_1691	uncharacterized protein	8.56	29.27
MRET_1692	pericentrin-AKAP-450 domain of centrosomal targeting protein	3.66	16.03
MRET_1693	uncharacterized protein	22.17	27.3
MRET_1694	uncharacterized protein	217.81	257.99
MRET_1695	ATP-dependent DNA helicase 2 subunit 2	39.68	71.17
MRET_1696	beta-1,4-mannosyltransferase	11.86	16.62
MRET_1697	ubiquitin conjugation factor E4 B	30.7	41.61
MRET_1698	translation initiation factor 2 subunit 1	44.72	145.31
MRET_1699	T-complex protein 1 subunit epsilon	158.43	146.88

MRET_1700	peptidyl-prolyl isomerase domain and WD repeat protein 1	30.08	38.82
MRET_1701	mitochondrial carrier protein	312.21	171.68
MRET_1702	uncharacterized protein	16.13	18.81
MRET_1703	uncharacterized protein	589.65	287.21
MRET_1704	Ca2+:H+ antiporter	35.44	44.42
MRET_1705	aspartate-semialdehyde dehydrogenase	77.4	126.35
MRET_1706	dihydroxy-acid dehydratase	78.58	153.21
MRET_1707	phosphatidylserine decarboxylase	30	49.59
MRET_1708	TRIAP1/MDM35 family protein	87.45	154.29
MRET_1709	cytochrome c	167.02	65.18
MRET_1710	GATA zinc finger	13.34	22.38
MRET_1711	large subunit ribosomal protein L15e	69.26	367.85
MRET_1712	large subunit ribosomal protein L5e	53.7	311.17
MRET_1713	repressible acid phosphatase	173.72	159.26
MRET_1714	uncharacterized protein	29.36	89.18
MRET_1715	uncharacterized protein	61.51	165.16
MRET_1716	NADH dehydrogenase (ubiquinone) 1 alpha subcomplex subunit 4	430.12	650.35
MRET_1717	delta14-sterol reductase	208.51	284.39
MRET_1718	prenylcysteine oxidase/farnesylcysteine lyase	34.68	47.3
MRET_1719	cofilin	482.8	546.5
MRET_1720	Ras homolog, member A	635.27	966.17
MRET_1721	guanosine-diphosphatase	191.38	114.66
MRET_1722	derlin	991.65	528.33
MRET_1723	m7GpppX diphosphatase	20.96	52.41
MRET_1724	N-terminal domain of NEFA-interacting nuclear protein NIP30	44.78	64.44
MRET_1725	tubulin-specific chaperone D	14.67	31.85
MRET_1726	osomolarity two-component system, phosphorelay intermediate protein YPD1	145.7	205.15
MRET_1727	ubiquitin carboxyl-terminal hydrolase MINDY-1/2	212.01	139.58
MRET_1728	proliferation-associated protein 1	90.9	113.18
MRET_1729	map microtubule affinity-regulating kinase	51.45	36.61
MRET_1730	uncharacterized protein	1130.38	688.4
MRET_1731	ATP-dependent Clp protease ATP-binding subunit ClpB	1288.82	1368.03
MRET_1732	protein BCP1	23.24	42.19
MRET_1733	carboxy-terminal domain RNA polymerase II polypeptide A small phosphatase	25.28	61.62
MRET_1734	p-loop containing nucleoside triphosphate hydrolase protein	35.55	80.45
MRET_1735	mitochondrial intermembrane space protein	2099.54	1621.16
MRET_1736	ribosome biogenesis protein BMS1	190.58	172.67

MRET_1737	cohesin loading factor subunit SCC2	52.85	77.18
MRET_1738	uncharacterized protein	158.59	103.64
MRET_1739	membrane associated DnaJ chaperone	11.65	46
MRET_1740	OPT oligopeptide transporter protein	19.4	36.77
MRET_1741	uncharacterized protein	19.98	58.21
MRET_1742	phosphatidyl synthase	115.52	195.96
MRET_1743	integral membrane protein required for ER to golgi transport	87.73	189.44
MRET_1744	uncharacterized protein	10.9	38.51
MRET_1745	acyl-coenzyme A thioesterase 13	78.06	251.05
MRET_1746	ATP-dependent RNA helicase DHX37/DHR1	33	73.96
MRET_1747	V-type H+-transporting ATPase subunit d	117.77	239.58
MRET_1748	histone deacetylase HOS3	352.37	343.41
MRET_1749	RIO kinase 1	10.68	29.3
MRET_1750	RhoGAP and Fes CIP4 domain protein	12.18	35.31
MRET_1751	U2 small nuclear ribonucleoprotein B''	573.46	553.12
MRET_1752	small subunit ribosomal protein S19e	112.13	648.62
MRET_1753	classical protein kinase C alpha type	232.05	425.87
MRET_1754	uncharacterized protein	56.7	163.63
MRET_1755	senataxin	9.89	16.82
MRET_1756	tetratricopeptide repeat domain protein	18.04	28.07
MRET_1757	DNA damage-binding protein 1	37.03	32.08
MRET_1758	uncharacterized protein	18.78	24.77
MRET_1759	voltage-dependent anion channel protein 2	785.42	822.62
MRET_1760	mRNA stabilization protein	1821.25	1545.5
MRET_1761	subunit 21 of mediator complex	123.3	152.98
MRET_1762	helix-loop-helix DNA-binding domain protein	138.7	239.65
MRET_1763	ATP-dependent DNA helicase Q1	9.22	14.31
MRET_1764	uncharacterized protein	56.85	47.39
MRET_1765	N6-L-threonylcarbamoyladenine synthase	388.71	314.72
MRET_1766	prolyl-tRNA synthetase	100.52	91.58
MRET_1767	glycoside hydrolase family 16 protein	206.22	120.99
MRET_1768	conserved hypothetical protein	29.96	30.07
MRET_1769	NADH dehydrogenase (ubiquinone) 1 alpha subcomplex subunit 9	110.26	148.58
MRET_1770	WD repeat protein 22	86.21	64.96
MRET_1771	adaptin ear-binding coat-associated protein 1/2	257.32	174.5
MRET_1772	uncharacterized protein	185.92	106.55
MRET_1773	oxidoreductase, short chain dehydrogenase reductase family	22.67	33.41

MRET_1774	ribonuclease P/MRP protein subunit POP1	17.25	33.77
MRET_1775	conserved hypothetical protein	149.08	61.88
MRET_1776	serine carboxypeptidase	149.01	82.83
MRET_1777	uncharacterized protein	292.48	218.75
MRET_1778	uncharacterized protein	63.02	106.31
MRET_1779	uncharacterized protein	37.33	34.56
MRET_1780	uncharacterized protein	131	201.08
MRET_1781	tyrosyl-DNA phosphodiesterase 1	611.78	315.76
MRET_1782	transcription initiation factor TFIIE subunit beta	92.71	83.08
MRET_1783	mitogen-activated protein kinase	99.03	115.94
MRET_1784	PHD finger domain protein	103.01	175.57
MRET_1785	casein kinase II subunit beta	57.92	90.71
MRET_1786	nucleoporin NUP159	28.88	60.85
MRET_1787	tRNA-specific adenosine deaminase 3	21.62	59.83
MRET_1788	Na+/H+ antiporter	44.86	132.22
MRET_1789	uncharacterized protein	197.59	395.52
MRET_1790	mitochondrial import receptor subunit TOM22	151.42	320.43
MRET_1791	enhancer of polycomb-like protein	855.45	547.04
MRET_1792	rRNA-processing protein	19.31	53.97
MRET_1793	U3 small nucleolar RNA-associated protein 3	14.54	35.55
MRET_1794	actin related protein 2/3 complex, subunit 1A/1B	1160.36	931.02
MRET_1795	phosphate transporter (Pho88)	72.03	210.38
MRET_1796	lipid intermediate transporter	34.19	98.17
MRET_1797	cullin 1	103.96	173.47
MRET_1798	elongation factor-2 kinase	407.09	404.55
MRET_1799	Elongation factor-2 kinase	714.93	779.13
MRET_1800	U4/U6 snRNA-associated-splicing factor PRP24	54.74	73.46
MRET_1801	DUF2373 domain protein	9.91	33.86
MRET_1802	ATP-dependent RNA helicase DHX33	22.12	49.95
MRET_1803	ubiquitin-protein ligase E3 C	93.34	56.42
MRET_1804	conserved hypothetical protein	115.86	166.53
MRET_1805	mitochondrial Rho GTPase 1	14.5	27.98
MRET_1806	HD family hydrolase	7.4	26.8
MRET_1807	sentrin-specific protease 7	11.21	28.66
MRET_1808	malate dehydrogenase	400.57	376.11
MRET_1809	malate dehydrogenase	953.98	867.9
MRET_1810	uncharacterized protein	53.6	60.24

MRET_1811	DEAD/DEAH box helicase	32.46	60.26
MRET_1812	pre-mRNA-processing factor 6	18.72	26.25
MRET_1813	DNA binding transcription factor	78.68	101.62
MRET_1814	protein SCO1/2	848.53	796.83
MRET_1815	nulp1-pending protein	34.83	53.92
MRET_1816	protoporphyrin/coproporphyrin ferrochelatase	66.88	64.29
MRET_1817	exportin-T	12	31.94
MRET_1818	uncharacterized protein	82.31	46.61
MRET_1819	capping protein (actin filament) muscle Z-line, alpha	175.89	79.95
MRET_1820	derlin	62.75	88.92
MRET_1821	mitochondrial mRNA processing protein PET127	27.61	58.85
MRET_1822	large subunit ribosomal protein L6e	320.63	840.62
MRET_1823	folylpolyglutamate synthase	19.36	46.8
MRET_1824	mitochondrial translocator assembly and maintenance protein 41	44.47	130.82
MRET_1825	vesicle transport protein	2412.99	2392.86
MRET_1826	GPI inositol-deacylase	29.86	78.22
MRET_1827	SH3 domain YSC84-like protein 1	1234.93	555.07
MRET_1828	calcineurin-binding protein	24.18	100.91
MRET_1829	large subunit ribosomal protein L36e	186.02	482.81
MRET_1830	translation initiation factor 3 subunit E	56.7	110.62
MRET_1831	NADH dehydrogenase (ubiquinone) 1 alpha subcomplex subunit 7	49.97	65.74
MRET_1832	DNA-directed RNA polymerase I subunit RPA49	167.09	115.92
MRET_1833	uncharacterized protein	107.73	183.42
MRET_1834	ADP-ribosylation factor 1	1972.67	1642.32
MRET_1835	uncharacterized protein	21.09	30.75
MRET_1836	uncharacterized protein	80.33	115.18
MRET_1837	thioredoxin-like protein	32.51	70.81
MRET_1838	uncharacterized protein	13.46	19.9
MRET_1839	tRNA pseudouridine55 synthase	41.06	46.99
MRET_1840	methionyl-tRNA formyltransferase	96.21	71.95
MRET_1841	DUF500 domain protein	147.33	157.64
MRET_1842	S-formylglutathione hydrolase	247.1	181.4
MRET_1843	DnaJ domain protein	104.41	135.88
MRET_1844	prenyl protein peptidase	68.5	109.5
MRET_1845	uncharacterized protein	1362	289.19
MRET_1846	3'(2'), 5'-bisphosphate nucleotidase	70.29	92.71
MRET_1847	nicotinate-nucleotide pyrophosphorylase (carboxylating)	216.36	185.52

MRET_1848	AAA domain (dynein-related subfamily)	182.39	107.5
MRET_1849	regulatory associated protein of mTOR	189	83.64
MRET_1850	importin-9	95.23	68.71
MRET_1851	zinc finger protein, C3H1 type	69.94	185.4
MRET_1852	uncharacterized protein	52.85	162.31
MRET_1853	BolA-like protein 3	75.88	84.57
MRET_1854	cell division cycle 2-like protein	6.62	25.1
MRET_1855	sterol-4alpha-carboxylate 3-dehydrogenase (decarboxylating)	109.54	125.75
MRET_1856	zinc finger protein	1092.6	1011.88
MRET_1857	diazepam-binding inhibitor (GABA receptor modulator, acyl-CoA-binding protein)	1083.04	2122.52
MRET_1858	oligosaccharyltransferase complex subunit delta (ribophorin II)	40.7	65.24
MRET_1859	transcriptional activator SPT8	109.25	82.96
MRET_1860	tRNAThr (cytosine32-N3)-methyltransferase	56.57	44.23
MRET_1861	threonine aldolase	240.19	95.53
MRET_1862	26S proteasome regulatory subunit N2	80.12	91.24
MRET_1863	prefoldin subunit 2	62.38	141
MRET_1864	NADH-ubiquinone oxidoreductase 12 kda subunit	155.36	348.73
MRET_1865	tRNA (guanine-N7-)-methyltransferase	33.79	50.77
MRET_1866	translation initiation factor 3 subunit F	221.26	276.93
MRET_1867	mitochondrial import receptor subunit TOM7	211.4	270.81
MRET_1868	N-alpha-acetyltransferase 40	36.59	43.9
MRET_1869	calcium/calmodulin-dependent protein kinase kinase 2	88.5	74.13
MRET_1870	putative transcription factor	1208.63	774.1
MRET_1871	uncharacterized protein	22.14	35.17
MRET_1872	cell growth-regulating nucleolar protein	24.14	41.31
MRET_1873	G1 S-specific cyclin	27.34	30.34
MRET_1874	large subunit ribosomal protein L30e	1154.35	1174.89
MRET_1875	large subunit ribosomal protein L37e	545.98	1400.66
MRET_1876	large subunit ribosomal protein L9e	232.14	831.28
MRET_1877	pre-mRNA cleavage complex 2 protein Pcf11	166.96	197
MRET_1878	conserved oligomeric golgi complex subunit 3	30.33	45.63
MRET_1879	NADPH2:quinone reductase	47.95	69.4
MRET_1880	aspartate kinase	125.48	120.33
MRET_1881	exportin-2 (importin alpha re-exporter)	163.25	389.81
MRET_1882	protein SSD1	63	54.86
MRET_1883	ATP-dependent RNA helicase DBP3	56.14	103.09
MRET_1884	WD repeat protein JIP5	24.81	40.16

MRET_1885	branched-chain amino acid aminotransferase	148.03	106.06
MRET_1886	alpha/beta-hydrolase	3.94	5.19
MRET_1887	small subunit ribosomal protein S29e	155.46	660.89
MRET_1888	N-terminal acetyltransferase 2	41.59	154.59
MRET_1889	ATP-dependent bile acid permease	78.16	89.82
MRET_1890	DNA mismatch repair protein	16.61	38.56
MRET_1891	proteasome assembly chaperone 2	13.34	37.41
MRET_1892	ATP-dependent bile acid permease	116.61	115.96
MRET_1893	CCR4-NOT transcription complex subunit 1	61.43	62.94
MRET_1894	protein involved in GPI anchor synthesis	64.74	71.45
MRET_1895	calcium/calmodulin-dependent protein kinase I	125.43	206.63
MRET_1896	histone-lysine N-methyltransferase, H3 lysine-79 specific	53.42	104.02
MRET_1897	methylated-DNA-protein-cysteine methyltransferase related protein	58.45	76.55
MRET_1898	uncharacterized protein	695	460.33
MRET_1899	phenylalanyl-tRNA synthetase alpha chain	68.82	76.55
MRET_1900	abhydrolase domain protein 12	83.77	161.85
MRET_1901	dehydrodolichyl diphosphate syntase complex subunit NUS1	10.7	19.82
MRET_1902	uncharacterized protein	19.32	35.95
MRET_1903	GTP cyclohydrolase II	51.31	43.47
MRET_1904	protein AATF/BFR2	101.99	143.54
MRET_1905	U4/U6 small nuclear ribonucleoprotein PRP3	123.88	121.71
MRET_1906	importin subunit alpha	33.75	79.12
MRET_1907	ankyrin repeat domain protein	11.68	20.77
MRET_1908	lipid-binding protein	36.69	56.15
MRET_1909	template-activating factor I	109.31	324.68
MRET_1910	uncharacterized protein	433.02	796.67
MRET_1911	E3 ubiquitin-protein ligase HECTD2	277.07	383.53
MRET_1912	essential RNA-binding component of cleavage and polyadenylation factor	62.47	50.3
MRET_1913	thioesterase	116.26	245.13
MRET_1914	anaphase-promoting complex subunit 5	82.61	109.43
MRET_1915	phosphatase with a broad substrate specificity	46.68	44.36
MRET_1916	uncharacterized protein	472.33	230.57
MRET_1917	NADH-ubiquinone oxidoreductase	713.04	1182.24
MRET_1918	cation efflux family protein	44.14	117.42
MRET_1919	essential protein that forms a complex with Rli1p and Yae1p	211.68	313.59
MRET_1920	uncharacterized protein	36.29	100.56
MRET_1921	HUS1 checkpoint protein	50.59	183.44

MRET_1922	oxidative stress survival svf1-like protein	62.73	157.52
MRET_1923	protein transport protein SEC61 subunit beta	151.54	272.61
MRET_1924	uncharacterized protein	127.67	621.46
MRET_1925	acetyltransferase (GNAT) family	17.11	99.61
MRET_1926	PPR repeat containing protein	34.65	71.21
MRET_1927	uncharacterized protein	361.25	195.13
MRET_1928	uncharacterized protein	6.65	13.56
MRET_1929	phosphomannomutase	57.68	94.17
MRET_1930	protein CWC21	297.31	309.76
MRET_1931	CCR4-NOT transcription complex subunit 9	31.81	51.72
MRET_1932	double-strand break repair protein MRE11	71.7	75.28
MRET_1933	phosphatidylglycerol phosphatidylinositol transfer protein	22.69	36.4
MRET_1934	RIO kinase 2	83.38	111.92
MRET_1935	palmitoyltransferase ZDHHC9/14/18	82.45	145.53
MRET_1936	sister chromatid separation protein	342.68	250.61
MRET_1937	uncharacterized protein	49.51	64.72
MRET_1938	20S proteasome subunit alpha 7	233	257.77
MRET_1939	U1 small nuclear ribonucleoprotein 70kDa	65.29	77.6
MRET_1940	dolichol kinase	85.19	74.83
MRET_1941	ESCRT-I complex subunit VPS28	72.7	77.24
MRET_1942	uncharacterized protein	185.92	75.63
MRET_1943	tRNA-dihydrouridine synthase 1	108.3	74.32
MRET_1944	kinetochore protein Spc7/SPC105	24.47	50.79
MRET_1945	ATP-dependent RNA helicase DDX52/ROK1	98.17	70.76
MRET_1946	nucleolar MIF4G domain protein 1	46.07	51.06
MRET_1947	minor histocompatibility antigen H13	70.43	122.81
MRET_1948	ribulose-phosphate 3-epimerase	463.64	417.47
MRET_1949	pre-mRNA-processing factor 17	251.73	176.01
MRET_1950	regulator of ribosome biosynthesis	17.93	60.09
MRET_1951	interactor of little elongation complex ELL subunit 2	21.91	46.38
MRET_1952	ADP-ribosylation factor related protein 1	180.07	177.19
MRET_1953	NADH dehydrogenase (ubiquinone) Fe-S protein 1	296.57	121.47
MRET_1954	integral membrane protein	50.82	79.26
MRET_1955	uridine kinase	145.22	142.56
MRET_1956	serine/threonine-protein phosphatase PP1 catalytic subunit	112.09	214.58
MRET_1957	CCR4-NOT complex subunit CAF16	32.41	45.76
MRET_1958	U3 small nucleolar RNA-associated protein 7	54.27	158.55

MRET_1959	glycosyl transferases group 1	1057.11	847.2
MRET_1960	WD domain, G-beta repeat protein	112.72	72.32
MRET_1961	RNA-binding protein 39	39.29	41.2
MRET_1962	general transcription factor IIIA	12.26	24.64
MRET_1963	guanyl-nucleotide exchange factor	22.17	95.74
MRET_1964	uncharacterized protein	1901.71	2027.31
MRET_1965	tubulin-tyrosine ligase family protein	41.9	62.53
MRET_1966	uncharacterized protein	33.49	85.52
MRET_1967	nucleolar protein 9	52.18	51.31
MRET_1968	argininosuccinate synthase	156.19	191.06
MRET_1969	BolA-like protein 1	406.97	254.57
MRET_1970	trehalose 6-phosphate synthase	39.94	106.28
MRET_1971	universal stress protein	90.18	122.74
MRET_1972	uncharacterized protein	34.14	17.7
MRET_1973	17beta-estradiol 17-dehydrogenase	71.99	86.01
MRET_1974	NAD+ synthase (glutamine-hydrolysing)	32.72	35.46
MRET_1975	uncharacterized protein	31.37	21.07
MRET_1976	uncharacterized protein	47	35.25
MRET_1977	conserved hypothetical protein	49.41	88.24
MRET_1978	cell polarity protein	52.26	64.97
MRET_1979	replication factor C subunit 2/4	30.16	67.12
MRET_1980	uncharacterized protein	43.33	43.78
MRET_1981	adiponectin receptor	311.53	181.12
MRET_1982	pyridoxamine 5'-phosphate oxidase	33.95	33.69
MRET_1983	mitochondrial protein	105.34	159.96
MRET_1984	Ras-related GTP-binding protein A/B	50.86	95.92
MRET_1985	uncharacterized protein	38.24	60.6
MRET_1986	sphingolipid C9-methyltransferase	56.61	62.27
MRET_1987	uncharacterized protein	132.38	100.32
MRET_1988	peptidyl-prolyl cis-trans isomerase NIMA-interacting 1	113.12	165.08
MRET_1989	tRNA A64-2'-O-ribosylphosphate transferase	13.69	22.88
MRET_1990	glycosyl hydrolase family 88	592.91	308.77
MRET_1991	isocitrate dehydrogenase IDP1	1676.11	1395.44
MRET_1992	mitochondrial ornithine carrier protein	402.18	432.57
MRET_1993	conserved hypothetical protein	161.77	117.74
MRET_1994	acid phosphatase	1125.79	757.06
MRET_1995	ditrans,polycis-polyprenyl diphosphate synthase	131.71	384.8

MRET_1996	uncharacterized protein	30.86	40.91
MRET_1997	coenzyme Q-binding protein COQ10	20.97	44.74
MRET_1998	NADPH-dependent medium chain alcohol dehydrogenase	44.73	125.5
MRET_1999	ubiquinol-cytochrome-c reductase complex subunit (QCR10)	154.74	215.53
MRET_2000	carbonic anhydrase	88.56	102.07
MRET_2001	uracil-DNA glycosylase	87.6	112.96
MRET_2002	ubiquitin carboxyl-terminal hydrolase L3	107.94	59.55
MRET_2003	uncharacterized protein	52.75	46.51
MRET_2004	PUA domain protein	158.23	95.64
MRET_2005	prolyl oligopeptidase	41.07	43.49
MRET_2006	large subunit GTPase 1	90.98	77.27
MRET_2007	U3 small nucleolar RNA-associated protein 25	19.54	25.55
MRET_2008	uncharacterized protein	33.58	51.84
MRET_2009	SHO1 osmosensor	974.17	1257.57
MRET_2010	cytochrome c oxidase subunit 5b	431.9	655.05
MRET_2011	cytochrome b561	36.88	52.69
MRET_2012	large subunit ribosomal protein L49	41.05	112.1
MRET_2013	protein transport protein SEC61 subunit gamma and related proteins	130.93	366.73
MRET_2014	large subunit ribosomal protein L35e	171.78	682.47
MRET_2015	uncharacterized protein	48.1	58.89
MRET_2016	Ras-related C3 botulinum toxin substrate 1	218.2	522.62
MRET_2017	uncharacterized protein	39.15	62.92
MRET_2018	uncharacterized protein	29.21	55.46
MRET_2019	nuclear protein localization protein 4 homolog	423.39	300.81
MRET_2020	cardiolipin-specific phospholipase	69.85	92.05
MRET_2021	recombination hotspot-binding protein	39.12	476.12
MRET_2022	CCAAT-binding factor complex subunit	98.84	186.93
MRET_2023	upstream activation factor subunit UAF30	166.8	351.19
MRET_2024	mitochondrial protein involved in assembly of succinate dehydrogenase	122.65	138.73
MRET_2025	conserved hypothetical protein	83.28	123.03
MRET_2026	prohibitin 1	1427.76	663.73
MRET_2027	uncharacterized protein	1386.33	301.62
MRET_2028	uncharacterized protein	248.11	160.55
MRET_2029	D-glycerate 3-kinase	329.73	238.24
MRET_2030	ssDNA-binding protein essential for mitochondrial genome maintenance	110.26	211.7
MRET_2031	sucrase/ferredoxin-like protein	201.23	81.6
MRET_2032	2-methoxy-6-polyprenyl-1,4-benzoquinol methylase	67.63	106.45

MRET_2033	uncharacterized protein	218.08	304.7
MRET_2034	inositol polyphosphate 5-phosphatase	165.04	158.35
MRET_2035	uncharacterized protein	310.22	227.54
MRET_2036	mitochondrial fission process protein 1	115.59	87.73
MRET_2037	thioredoxin	13276.84	8853.33
MRET_2038	monooxygenase	507.76	227.01
MRET_2039	cellular morphogenesis protein	157.75	179.18
MRET_2040	Pescadillo homolog	110.71	237.66
MRET_2041	small nuclear ribonucleoprotein	314.17	232.29
MRET_2042	fumarate hydratase, class II	634.43	262.16
MRET_2043	conserved hypothetical protein	184.3	64.73
MRET_2044	37S ribosomal protein RSM22	119.8	42.82
MRET_2045	protein DGCR14	12.06	14.26
MRET_2046	guanine nucleotide-binding protein G(I)/G(S)/G(T) subunit beta-1	36.23	48.33
MRET_2047	solute carrier family 24 (sodium potassium calcium exchanger), member 1	145.75	176.99
MRET_2048	thioesterase	878.88	183.68
MRET_2049	ribosome assembly protein RRB1	108.46	205.97
MRET_2050	phosphoribosylamine--glycine ligase/phosphoribosylformylglycinamidine cyclo-ligase	371.5	246.11
MRET_2051	transporter	74.28	64.88
MRET_2052	uncharacterized protein	40.99	38.88
MRET_2053	serine/threonine-protein kinase haspin	44.71	54.98
MRET_2054	large subunit ribosomal protein L1	109.67	98.91
MRET_2055	SUR7/PalI family protein	96.88	72.56
MRET_2056	uncharacterized protein	44.73	37.16
MRET_2057	component of the EKC/KEOPS complex	39.74	39.78
MRET_2058	adenine phosphoribosyltransferase	50.99	84.87
MRET_2059	uncharacterized protein	28.02	33.07
MRET_2060	uncharacterized protein	33.84	48.95
MRET_2061	citrate lyase subunit beta-like protein	254	143.44
MRET_2062	ATP-binding cassette, subfamily B (MDR/TAP), member 6	153.43	58.25
MRET_2063	uncharacterized protein	197.48	283.64
MRET_2064	ER membrane protein SH3	822.13	614.89
MRET_2065	mitochondrial pyruvate dehydrogenase kinase	77.47	102.99
MRET_2066	vesicle transport through interaction with t-SNAREs 1	1784.08	1122.44
MRET_2067	sensor protein CreC	54.56	137.77
MRET_2068	IGR motif protein	32.33	91.77
MRET_2069	uncharacterized protein	22.26	70.2

MRET_2070	platelet-activating factor acetylhydrolase	15.86	45.44
MRET_2071	ubiquitin-conjugating enzyme (huntingtin interacting protein 2)	75.94	275.46
MRET_2072	oxalate---CoA ligase	160.96	177.51
MRET_2073	ATP-dependent NAD(P)H-hydrate dehydratase	74.18	91.58
MRET_2074	uncharacterized protein	39.54	116.54
MRET_2075	uncharacterized protein	130.56	168.69
MRET_2076	cytidine deaminase	110.45	143.68
MRET_2077	5-oxoprolinase (ATP-hydrolysing)	246.73	152.85
MRET_2078	cytochrome c oxidase subunit 23	328.32	165.25
MRET_2079	WD repeat protein 61	179.91	134.14
MRET_2080	uncharacterized protein	77.46	50.72
MRET_2081	solute carrier family 25 (mitochondrial carnitine/acylcarnitine transporter), member 20/29	167.66	91.61
MRET_2082	coatomer subunit epsilon	53.95	74.11
MRET_2083	cytochrome c peroxidase	725.57	282.01
MRET_2084	uncharacterized protein	86.63	68.82
MRET_2085	Rab GDP dissociation inhibitor	577.16	461.74
MRET_2086	adenylate kinase	391.44	326.82
MRET_2087	large subunit ribosomal protein L22	71.84	109.11
MRET_2088	aldehyde dehydrogenase (NAD+)	96.73	116.46
MRET_2089	peptidyl-prolyl cis-trans isomerase B (cyclophilin B)	269.77	349.71
MRET_2090	RhoGAP	15.13	31.48
MRET_2091	subunit of the heterohexameric cochaperone prefoldin complex	52.95	113.14
MRET_2092	peptidyl-prolyl isomerase D	129.96	131.21
MRET_2093	alanine-glyoxylate transaminase/serine-glyoxylate transaminase/serine-pyruvate transaminase	221.97	156.8
MRET_2094	putative methyltransferase	146.94	67.28
MRET_2095	actin related protein 2/3 complex, subunit 2	203.6	181.85
MRET_2096	ATP-binding protein involved in chromosome partitioning	221.45	127.96
MRET_2097	succinate dehydrogenase (ubiquinone) flavoprotein subunit	191.34	87.18
MRET_2098	altered inheritance of mitochondria protein 13	143.84	252.14
MRET_2099	zinc finger protein, C2HC5-type	34.03	61.72
MRET_2100	F-box and leucine-rich repeat protein 10/11	78.97	62.23
MRET_2101	THO complex subunit 1	63.12	86.6
MRET_2102	RNA-binding protein 8A	104.39	279.98
MRET_2103	small subunit ribosomal protein S27Ae	4467.41	5795.3
MRET_2104	dual specificity kinase	224.64	149.69
MRET_2105	DUF1713 domain protein	230.68	153.07
MRET_2106	mitochondrial 54S ribosomal protein RML2	38.02	86.28

MRET_2107	S-phase kinase-associated protein 1	623.24	706.14
MRET_2108	SNF2 chromatin remodeling protein	220.74	100.33
MRET_2109	NADH dehydrogenase (ubiquinone) 1 alpha subcomplex subunit 1	233.02	172.97
MRET_2110	mitochondrial import inner membrane translocase subunit TIM44	378.56	342.93
MRET_2111	CCCH zinc finger and SMR	40.42	43.98
MRET_2112	RNA polymerase II-associated factor 1	84.32	121.45
MRET_2113	PAB-dependent poly(A)-specific ribonuclease subunit 2	81.83	61.21
MRET_2114	uncharacterized protein	137.71	131.38
MRET_2115	SRP40, C-terminal domain protein	21.67	36.65
MRET_2116	sugar transporter	402.17	294.19
MRET_2117	proteasome activator subunit 4	48.39	39.54
MRET_2118	YdiU domain protein	388.45	138.84
MRET_2119	phosphatidylinositol glycan, class M	11.44	33.08
MRET_2120	magnesium transporter	17.5	16.36
MRET_2121	SUR7/PalI family protein	276.23	129.96
MRET_2122	uncharacterized protein	99.72	68.07
MRET_2123	uncharacterized protein	24825.52	20449.66
MRET_2124	acyl-CoA dehydrogenase	460.21	397.13
MRET_2125	CBS PB1 domain protein	52.19	75.57
MRET_2126	uncharacterized protein	91.13	89.4
MRET_2127	phospholipid:diacylglycerol acyltransferase	91.59	58.99
MRET_2128	diphthamide biosynthesis protein 4	36.2	44.75
MRET_2129	sentrin-specific protease 1	32.68	82.93
MRET_2130	regulatory factor Sgt1	51.36	96.97
MRET_2131	NADH dehydrogenase (ubiquinone) Fe-S protein 3	161.73	224.54
MRET_2132	pre-mRNA-splicing factor SPF27	110.7	165.78
MRET_2133	rRNA processing protein RRP15	82.59	176.19
MRET_2134	succinyl-CoA synthetase beta subunit	289.66	367.09
MRET_2135	mitochondrial 37S ribosomal protein MRPS8	66.52	119.07
MRET_2136	S-adenosylmethionine-dependent methyltransferase	77.79	174.59
MRET_2137	small subunit ribosomal protein S27e	449.38	944.15
MRET_2138	large subunit ribosomal protein L19e	298.07	906.54
MRET_2139	protein of unknown function (DUF2413)	31.16	120.53
MRET_2140	uncharacterized protein	48.79	369.47
MRET_2141	serine/threonine-protein phosphatase 2B catalytic subunit	71.46	91.4
MRET_2142	20S proteasome subunit beta 6	124.9	174.2
MRET_2143	Dsk2-ubiquitin-like protein	199.44	204.79

MRET_2144	glycerol-3-phosphate phosphatase	222.55	120.6
MRET_2145	glycerol-3-phosphate phosphatase	27.7	72.37
MRET_2146	SNF1 kinase complex beta-subunit Gal83	32.6	60.21
MRET_2147	anaphase-promoting complex subunit 11	20.88	33.53
MRET_2148	kinesin family member C1	36.21	83.93
MRET_2149	nuclear segregation protein	64.05	205.88
MRET_2150	CTD kinase subunit alpha	68.2	77.35
MRET_2151	fungal Zn(2)-Cys(6) binuclear cluster domain protein	875.7	452.31
MRET_2152	xeroderma pigmentosum group C-complementing protein	22.92	29.92
MRET_2153	carnosine N-methyltransferase	15.3	39.2
MRET_2154	cytomegalovirus gH-receptor family protein	35.95	66.27
MRET_2155	uncharacterized protein	66.64	70.88
MRET_2156	rRNA-processing protein EBP2	569.35	2751.51
MRET_2157	RNA polymerase-associated protein LEO1	46.81	67.08
MRET_2158	inverted formin	254.64	297.81
MRET_2159	zinc finger protein	76.69	50.45
MRET_2160	glucose-6-phosphate 1-dehydrogenase	1176.04	852.85
MRET_2161	superoxide dismutase, Fe-Mn family	967.52	989.01
MRET_2162	zinc finger protein (RING finger)	36.84	100.17
MRET_2163	ATP-dependent RNA helicase UAP56/SUB2	133.72	233.55
MRET_2164	methyltransferase-like protein 13	28.83	36.13
MRET_2165	pre-mRNA-splicing factor spp2	32.04	53.96
MRET_2166	saccharopepsin	1269.73	878.88
MRET_2167	uncharacterized protein	523.07	548.62
MRET_2168	dynein light chain roadblock-type	50.54	83.23
MRET_2169	mitotic spindle assembly checkpoint protein MAD1	136.61	110.87
MRET_2170	bZIP transcription factor	1022.99	488.75
MRET_2171	tubulin-specific chaperone A	100.35	55.5
MRET_2172	ribonuclease Z	63.96	74.09
MRET_2173	serine/threonine-protein kinase PRP4	214.49	263.41
MRET_2174	ATP-dependent RNA helicase DDX5/DBP2	118.54	237.25
MRET_2175	DNA-(apurinic or apyrimidinic site) lyase	40.16	36.38
MRET_2176	N6-L-threonylcarbamoyladenine synthase	126.47	81.14
MRET_2177	uncharacterized protein	176.89	160.42
MRET_2178	ribosomal protein L30p/L7e	562.1	1519.89
MRET_2179	4-hydroxybenzoate polyprenyltransferase, mitochondrial	707.89	437.85
MRET_2180	4-hydroxybenzoate polyprenyltransferase	61.17	97.91

MRET_2181	uncharacterized protein	236.76	265.11
MRET_2182	uncharacterized protein	37.72	63.96
MRET_2183	uncharacterized protein	38.54	50.8
MRET_2184	chitin deacetylase	230.83	317.22
MRET_2185	ribosome biogenesis protein YTM1	105.51	104.26
MRET_2186	endothelin-converting enzyme	290.08	146.16
MRET_2187	endothelin-converting enzyme	43.64	76.19
MRET_2188	YagE family protein	148.01	90.25
MRET_2189	coiled-coil domain protein	123.54	82.63
MRET_2190	4'-phosphopantetheinyl transferase	68.28	46.29
MRET_2191	vacuolar protein sorting 55 superfamily	55.22	97.45
MRET_2192	CCR4-NOT transcription complex subunit 4	407.34	460.17
MRET_2193	trafficking protein particle complex subunit 9	74.69	32.72
MRET_2194	uncharacterized protein	111.75	172
MRET_2195	cysteine synthase A	40.98	82.34
MRET_2196	helix-loop-helix DNA-binding domain protein	42.62	96.84
MRET_2197	beta-glucan synthesis-associated protein KRE6	24.37	67
MRET_2198	AP complex subunit beta	40.52	37.42
MRET_2199	U3 small nucleolar RNA-associated protein 15	68.22	101.17
MRET_2200	copper chaperone	2525.18	1290.35
MRET_2201	small subunit ribosomal protein S21e	108.98	399.62
MRET_2202	pumilio-family RNA binding repeat protein	43.03	47.9
MRET_2203	TBC1 domain family member 15	28.1	25.8
MRET_2204	uncharacterized protein	18.08	21.89
MRET_2205	3-oxoacid CoA-transferase	1014.8	652.94
MRET_2206	metal transporter CNNM	410.62	148.97
MRET_2207	DnaJ homolog subfamily A member 2	486.51	535.44
MRET_2208	uncharacterized protein	27.49	93.79
MRET_2209	mitochondrial 54S ribosomal protein YmL38 YmL34	30.39	77.03
MRET_2210	tRNA (guanine26-N2/guanine27-N2)-dimethyltransferase	35.13	52.63
MRET_2211	L-ascorbic acid binding protein	400.66	384.69
MRET_2212	peroxin-1	17.71	22.99
MRET_2213	mannosyl phosphorylinositol ceramide synthase SUR1	21.72	33
MRET_2214	metal resistance protein YCF1	22.58	30.83
MRET_2215	homoaconitase	118.37	90.43
MRET_2216	suppressor of G2 allele of SKP1	95.79	83.71
MRET_2217	alpha-1,3-glucosyltransferase	49.24	38.36

MRET_2218	Ras suppressor protein 1	66.06	59.25
MRET_2219	orotidine 5-phosphate decarboxylase	91.05	98.72
MRET_2220	kinesin family member 5	38.1	73.24
MRET_2221	chorismate mutase	17.94	11.71
MRET_2222	E3 ubiquitin-protein ligase listerin	100.37	35.73
MRET_2223	DUF2346 domain protein	206.23	267.85
MRET_2224	INO80 complex subunit C	82.64	187.17
MRET_2225	endonuclease/exonuclease/phosphatase family	465.51	425.28
MRET_2226	uncharacterized protein	494.48	423.54
MRET_2227	uncharacterized protein	632.99	437.95
MRET_2228	uncharacterized protein	34.34	58.63
MRET_2229	uncharacterized protein	12.91	18.58
MRET_2230	pyruvate decarboxylase	366.35	179.11
MRET_2231	protein AIR1/2	32.15	57.01
MRET_2232	cell division control protein 6	15.25	27.31
MRET_2233	uncharacterized protein	36.16	56.77
MRET_2234	mitochondrial division protein 1	23.28	49.89
MRET_2235	GET complex subunit GET2	115.97	130.87
MRET_2236	DNA topoisomerase II	40.57	46.52
MRET_2237	3-hydroxyisobutyryl-CoA hydrolase	264.63	230.46
MRET_2238	uncharacterized protein	63.34	73.74
MRET_2239	phosphatidylinositol glycan, class O	138.79	94.89
MRET_2240	COP9 signalosome complex subunit 12	39.48	58.69
MRET_2241	DNA polymerase delta subunit 2	21.73	26.6
MRET_2242	tRNA-dihydrouridine synthase 3	75.99	68.1
MRET_2243	methionyl-tRNA synthetase	90.84	155.81
MRET_2244	protein phosphatase inhibitor 2 (IPP-2)	22.01	26.25
MRET_2245	lysophospholipid hydrolase	19.86	24.12
MRET_2246	glutamine synthetase	1161.99	1498.09
MRET_2247	protein phosphatase 4 regulatory subunit 3	126.89	65.03
MRET_2248	YEATS domain protein 4	31.57	46.83
MRET_2249	GDP-mannose transporter	22.06	39.22
MRET_2250	cell division control protein 14	11.56	20.1
MRET_2251	small nuclear ribonucleoprotein E	69.24	189.93
MRET_2252	protein MAK11	69.38	172.68
MRET_2253	transporter	15.8	18.8
MRET_2254	ESCRT-II complex subunit VPS25	15.22	15.93

MRET_2255	uncharacterized protein	38.54	39.94
MRET_2256	uracil phosphoribosyltransferase	26.21	37.4
MRET_2257	LYR motif protein 4	536.04	528.74
MRET_2258	serine/threonine-protein kinase	79.23	26.97
MRET_2259	zinc cluster transcription factor Rds2	26.77	64.04
MRET_2260	ornithine decarboxylase	18.71	37.9
MRET_2261	Sds3-like protein	46.62	70.69
MRET_2262	NADH dehydrogenase (ubiquinone) 1 alpha subcomplex subunit 5	200.13	274.76
MRET_2263	nitric oxide synthase-interacting protein	129.87	139.46
MRET_2264	mitochondrial fission protein FIS1	628.03	440.89
MRET_2265	sorting nexin-4	246.91	190.61
MRET_2266	clathrin heavy chain	148.06	125.18
MRET_2267	26S proteasome regulatory subunit T4	102.45	133.44
MRET_2268	1-phosphatidylinositol-3-phosphate 5-kinase	56	39.33
MRET_2269	uncharacterized protein	87.71	64.61
MRET_2270	ADP-ribosylation factor-binding protein GGA	58.56	86.81
MRET_2271	uncharacterized protein	19.52	38.43
MRET_2272	uncharacterized protein	29.03	65.27
MRET_2273	arginyl-tRNA synthetase	36.55	88.52
MRET_2274	nuclear GTP-binding protein	190.06	262.52
MRET_2275	nuclear GTP-binding protein	141.86	184.28
MRET_2276	viral A-type inclusion protein repeat protein	5.26	48.14
MRET_2277	gamma-glutamyltranspeptidase/glutathione hydrolase	166.4	71.52
MRET_2278	mitochondrial serine protease	116.36	50.93
MRET_2279	T-complex protein 1 subunit eta	208.02	195
MRET_2280	protein ATS1	348	74.28
MRET_2281	membrane associated protein	192.42	60.38
MRET_2282	large subunit ribosomal protein L3	195.49	242.85
MRET_2283	cleavage and polyadenylation specificity factor subunit 2	37.83	31.09
MRET_2284	uncharacterized protein	510.68	309.73
MRET_2285	PHD finger domain protein	61.2	71.33
MRET_2286	U3 small nucleolar RNA-associated protein 10	100.9	47.71
MRET_2287	flap endonuclease-1	57.67	28.81
MRET_2288	uncharacterized protein	27.01	30.95
MRET_2289	diphosphoinositol-polyphosphate diphosphatase	80.1	80.21
MRET_2290	uncharacterized protein	125.37	201.04
MRET_2291	uncharacterized protein	32.43	29.17

MRET_2292	large subunit ribosomal protein L7Ae	1816.55	2761.65
MRET_2293	putative stress-responsive nuclear envelope protein	686.82	385.08
MRET_2294	DNA repair protein RAD50	40.76	65.34
MRET_2295	palmitoyltransferase ZDHHC2/15/20	754.64	354.34
MRET_2296	ubiquitin-like 1-activating enzyme E1 A	584.77	296.24
MRET_2297	uncharacterized protein	297.4	298.97
MRET_2298	RNA polymerase I-specific transcription initiation factor RRN7	74.5	55.47
MRET_2299	nucleoprotein TPR	40.73	55.3
MRET_2300	tail-anchored protein insertion receptor	35.25	156.2
MRET_2301	glutamine amidotransferase	74.93	124.92
MRET_2302	conserved hypothetical protein	273.37	378.43
MRET_2303	p24 family protein alpha	67.4	120.08
MRET_2304	chloride channel 3/4/5	42.39	43.7
MRET_2305	mRNA export factor	5.7	8.22
MRET_2306	dynein light intermediate chain 1, cytosolic	34.24	43.29
MRET_2307	uncharacterized protein	5.9	21.51
MRET_2308	PPR repeat containing protein	64.42	48.51
MRET_2309	ATP-binding cassette, subfamily D (ALD), peroxisomal long-chain fatty acid import protein	73.82	61.67
MRET_2310	CCR4-NOT transcriptional complex subunit CAF120	64.97	53.54
MRET_2311	gluconokinase	9.29	5.48
MRET_2312	uncharacterized protein	41.64	40.07
MRET_2313	fungal Zn(2)-Cys(6) binuclear cluster domain protein	48.34	75.45
MRET_2314	nucleoporin POM152	88.02	51.29
MRET_2315	large subunit ribosomal protein L33	60.91	96.47
MRET_2316	uncharacterized protein	107.41	119.71
MRET_2317	ribosomal protein L23	183.3	180.02
MRET_2318	CTD nuclear envelope phosphatase 1	23.29	23.47
MRET_2319	serine/threonine-protein kinase Chk1	114.78	75.84
MRET_2320	DUF2340 domain protein	151.36	119.95
MRET_2321	sorting nexin	48.99	34.03
MRET_2322	DNA excision repair protein ERCC-5	377.35	197.69
MRET_2323	NADH dehydrogenase (ubiquinone) 1 beta subcomplex subunit 8	198.83	159.31
MRET_2324	serine/threonine-protein phosphatase 2A catalytic subunit	1217.26	1314.11
MRET_2325	DNA replication licensing factor MCM2	36.04	47.82
MRET_2326	serine/threonine-protein kinase	29.52	42.43
MRET_2327	ubiquitin-conjugating enzyme E2 J2	87.77	164.96
MRET_2328	uncharacterized protein	30.41	51.9

MRET_2329	ABC transporter	131.71	195.86
MRET_2330	ABC transporter	105.07	88.01
MRET_2331	syntaxin 6	21.38	29.89
MRET_2332	UDP-glucose:glycoprotein glucosyltransferase	52.38	56.18
MRET_2333	SNARE associated golgi protein	59.44	69.19
MRET_2334	transferase CAF17, mitochondrial	117.61	143.36
MRET_2335	Bacterial low temperature requirement A protein (LtrA)	39.15	92.37
MRET_2336	5'-nucleotidase	59.56	61.99
MRET_2337	HMG (high mobility group) box protein	282.15	224.13
MRET_2338	glucose-6-phosphate isomerase	405.45	288.99
MRET_2339	KH domain protein	203.37	162.75
MRET_2340	uncharacterized protein	15.01	22.85
MRET_2341	uncharacterized protein	46.89	145.63
MRET_2342	uncharacterized protein	110.4	286.67
MRET_2343	DNA polymerase epsilon subunit 1	62	45.49
MRET_2344	cytosolic Fe-S cluster assembly factor NBP35	323	277.88
MRET_2345	WD repeat and FYVE domain protein 3	117.46	47.14
MRET_2346	THO complex subunit 4	390.45	522.36
MRET_2347	ubiquinol-cytochrome c reductase subunit 8	385.47	533.64
MRET_2348	conserved hypothetical protein	127.52	160
MRET_2349	mitochondrial NADH kinase	45.96	97.91
MRET_2350	histone H3	3048.02	2446.42
MRET_2351	cell division cycle 20-like protein 1, cofactor of APC complex	52.26	108.48
MRET_2352	GMP synthase (glutamine-hydrolysing)	49.09	79.37
MRET_2353	cell cycle arrest protein BUB3	128.64	131.09
MRET_2354	cystathionine gamma-synthase	78.03	103.42
MRET_2355	syntaxin 5	37.53	75.44
MRET_2356	succinate dehydrogenase (ubiquinone) cytochrome b560 subunit	53.04	73.16
MRET_2357	gamma-tubulin complex component 2	31.32	75.13
MRET_2358	uroporphyrin-III C-methyltransferase	25.83	59.66
MRET_2359	S-adenosylmethionine decarboxylase	57.11	84.9
MRET_2360	zinc metalloprotease	69.08	66.11
MRET_2361	putative MFS transporter, AGZA family, xanthine/uracil permease	9.63	23.94
MRET_2362	uncharacterized protein	145.23	276.68
MRET_2363	uncharacterized protein	603.25	325.78
MRET_2364	reverse transcriptase	635.32	207.81
MRET_2365	phospholipase C	187.36	764.21

MRET_2366	aryl-alcohol dehydrogenase	571.39	416.95
MRET_2367	uncharacterized protein	604.42	354.04
MRET_2368	protein phosphatase methylesterase 1	237.84	93.25
MRET_2369	uncharacterized protein	63.79	56.58
MRET_2370	uncharacterized protein	59.44	40.03
MRET_2371	tRNA modification GTPase	28.26	45.79
MRET_2372	uncharacterized protein	56.86	46.6
MRET_2373	uncharacterized protein	119.8	125.19
MRET_2374	asparagine	46.34	26.12
MRET_2375	DUF757 domain protein	135.25	213.88
MRET_2376	NADH-ubiquinone oxidoreductase subunit	301.07	522.11
MRET_2377	multifunctional beta-oxidation protein	526.27	312.25
MRET_2378	small nuclear ribonucleoprotein D2	21.65	116.23
MRET_2379	glutamyl-tRNA synthetase	42.71	54.32
MRET_2380	dihydrofolate reductase	20.65	27.3
MRET_2381	2'-phosphotransferase	31.26	35.94
MRET_2382	V-type H+-transporting ATPase subunit C	128.94	131.56
MRET_2383	RNA-binding motif protein, X-linked 2	95.87	72.82
MRET_2384	protein phosphatase PTC6	37.08	32.85
MRET_2385	pre-rRNA processing protein Esf1	215.86	191.04
MRET_2386	transcription elongation factor SPT5	160.82	338.58
MRET_2387	elongation factor Tu	296.28	341.9
MRET_2388	large subunit ribosomal protein L31e	125.23	512.9
MRET_2389	SMN domain protein	48.01	89.01
MRET_2390	alpha/beta-hydrolase	50.45	50.3
MRET_2391	subunit of ATP-dependent Isw2p-Itc1p chromatin remodeling complex	31.56	38.49
MRET_2392	U6 snRNA-associated Sm-like protein LSm5	672.31	654.36
MRET_2393	calcineurin-like phosphoesterase	167.92	61.3
MRET_2394	ketol-acid reductoisomerase	242.01	186
MRET_2395	homoaconitate hydratase	109.95	91.69
MRET_2396	tRNA dimethylallyltransferase	127.16	95.07
MRET_2397	leukotriene-A4 hydrolase	67.71	47.89
MRET_2398	uncharacterized protein	19.59	31.93
MRET_2399	uncharacterized protein	27.22	24.96
MRET_2400	uncharacterized protein	1927.28	1550.38
MRET_2401	pyruvate kinase	531.03	278.7
MRET_2402	mediator of RNA polymerase II transcription subunit 5	9.69	12.1

MRET_2403	NADH dehydrogenase (ubiquinone) 1 alpha subcomplex subunit 5	155.89	112.62
MRET_2404	alpha-soluble NSF attachment protein	100.21	109.13
MRET_2405	aspartyl-tRNA(Asn)/glutamyl-tRNA(Gln) amidotransferase subunit B	131.92	69.45
MRET_2406	serine/threonine-protein phosphatase 2A activator	19.39	23.75
MRET_2407	threonyl-tRNA synthetase	35.05	59.46
MRET_2408	uncharacterized protein	43.25	105.3
MRET_2409	AT-rich interactive domain protein	21.17	31.5
MRET_2410	uncharacterized protein	17.78	38.65
MRET_2411	farnesyl-diphosphate farnesyltransferase	229.77	199.03
MRET_2412	protein KRI1	23.86	56.68
MRET_2413	U3 small nucleolar RNA-associated protein 20	34.14	32.11
MRET_2414	uncharacterized protein	58.22	48.75
MRET_2415	SEL1 domain protein	210.39	115.48
MRET_2416	ER membrane protein that plays a central role in ERAD	57.97	47.9
MRET_2417	heat shock protein 70 homolog LHS1	95.58	94.56
MRET_2418	Ca2+-transporting ATPase	35.13	37.37
MRET_2419	regulator of chromosome condensation (RCC1) repeat protein	65.77	65.57
MRET_2420	ribonuclease P protein subunit POP4	168.49	118.86
MRET_2421	saccharopine dehydrogenase	3682.84	2228.95
MRET_2422	T-complex protein 1 subunit theta	77.61	124.82
MRET_2423	transportin-1	38.3	39.91
MRET_2424	xylulokinase	165.22	95.5
MRET_2425	geranylgeranyl diphosphate synthase, type III	148.66	70.87
MRET_2426	DNA-directed RNA polymerases I, II, and III subunit RPABC3	45.49	92.21
MRET_2427	TBC1 domain family member 2	240.47	155.44
MRET_2428	RNA exonuclease 4	21.3	16.01
MRET_2429	uncharacterized protein	95.25	56.03
MRET_2430	phospholipid-transporting ATPase	55.36	57.02
MRET_2431	uncharacterized protein	72.16	41.8
MRET_2432	alkaline ceramidase	186.06	123.3
MRET_2433	DUF850 domain protein	61.41	84.39
MRET_2434	structural maintenance of chromosomes protein	26.46	28.92
MRET_2435	uncharacterized protein	22.04	35.61
MRET_2436	bZIP transcription factor	333.42	238.02
MRET_2437	transcription initiation factor TFIID subunit 10	295.2	312.92
MRET_2438	uncharacterized protein	25.37	26.37
MRET_2439	leucine-rich repeat protein	44.83	58.04

MRET_2440	solute carrier family 39 (zinc transporter), member 9	454.94	210.46
MRET_2441	2-nitropropane dioxygenase	369.22	344.15
MRET_2442	ER lumen protein retaining receptor	1261.74	343.69
MRET_2443	negative regulator of differentiation 1	116.16	70.76
MRET_2444	solute carrier family 25 (mitochondrial citrate transporter), member 1	242.9	125.88
MRET_2445	cell polarity protein	57.63	38.24
MRET_2446	mitochondrial matrix iron chaperone	42.17	34.49
MRET_2447	protein kinase A	71.47	78.29
MRET_2448	cerevisin	151.82	120.8
MRET_2449	asparaginyl-tRNA synthetase	24.87	44.99
MRET_2450	essential protein required for maturation of 18S rRNA	24.34	47.33
MRET_2451	transcriptional activator SPT7	154.53	157.38
MRET_2452	translation initiation factor 4G	1168.35	1749.77
MRET_2453	uncharacterized protein	90.24	29.57
MRET_2454	AHNAK nucleoprotein	561	1233.22
MRET_2455	mitochondrial GTPase MTG1	287.74	332.45
MRET_2456	uncharacterized protein	8.67	16.44
MRET_2457	pseudouridine synthase	41.3	57.45
MRET_2458	uncharacterized protein	140.42	112.53
MRET_2459	nuclear polyadenylated RNA-binding protein	363.8	291.52
MRET_2460	U6 snRNA-associated Sm-like protein LSm4	57.71	169.08
MRET_2461	peptidyl-prolyl cis-trans isomerase	92.18	65.88
MRET_2462	mediator of RNA polymerase II transcription subunit 17, fungi type	125.9	84.13
MRET_2463	histidinol-phosphate aminotransferase	21.42	46.16
MRET_2464	pre-mRNA-splicing factor CDC5/CEF1	179.82	159.03
MRET_2465	vacuolar protein sorting-associated protein	356.82	221.97
MRET_2466	uncharacterized protein	41.06	116.01
MRET_2467	calcium sensor Efh	59.99	72.34
MRET_2468	nicotinate phosphoribosyltransferase	528.14	487.1
MRET_2469	dynactin 5	805.55	425.13
MRET_2470	dynamin-binding protein	433.17	183.42
MRET_2471	glucan 1,3-beta-glucosidase	27.25	55.43
MRET_2472	Sad1 UNC domain protein	12.79	25.62
MRET_2473	vacuole protein	70.49	105.02
MRET_2474	potassium transport protein	304.89	39.89
MRET_2475	PITH domain protein	74.87	55.48
MRET_2476	SWI/SNF-related matrix-associated actin-dependent regulator of chromatin subfamily B member 1	82.56	52.36

MRET_2477	small subunit ribosomal protein S2	18.64	55.39
MRET_2478	heat shock 70kDa protein 1/2/6/8	104.41	292.78
MRET_2479	ATP-binding cassette, subfamily B (MDR/TAP), member 1	12	9.69
MRET_2480	cyclin	74.61	8.3
MRET_2481	translational activator GCN1	10.43	17.68
MRET_2482	guanine nucleotide-binding protein G(i) subunit alpha	38.13	64.59
MRET_2483	L-lactate dehydrogenase (cytochrome)	134.75	221.67
MRET_2484	intracellular protein transport protein USO1	60.13	55.36
MRET_2485	uncharacterized protein	19.56	26.18
MRET_2486	WD repeat protein 59	19.07	23.18
MRET_2487	uncharacterized protein	24.58	63.45
MRET_2488	tyrosine-protein phosphatase 2/3	17.12	27.55
MRET_2489	lectin, mannose-binding 2	41.2	82.9
MRET_2490	oligoribonuclease	15.4	18.63
MRET_2491	calpain-7	73.98	62.35
MRET_2492	3-methyl-2-oxobutanoate hydroxymethyltransferase	28.23	53.83
MRET_2493	ABC transporter	78.86	106.47
MRET_2494	mannosyl-oligosaccharide alpha-1,3-glucosidase	76.26	70.47
MRET_2495	translation initiation factor 2A	188.59	150.1
MRET_2496	type I protein arginine methyltransferase	169.73	159.54
MRET_2497	uncharacterized protein	19.15	51.62
MRET_2498	uncharacterized protein	19.97	42.7
MRET_2499	autophagy-related protein 101	111.9	39.58
MRET_2500	zinc metalloprotease	94.64	59.67
MRET_2501	tRNA uridine 5-carboxymethylaminomethyl modification enzyme	178.26	109.81
MRET_2502	GTP binding protein	28.84	33.27
MRET_2503	uncharacterized protein	26.55	29.35
MRET_2504	inositol phospholipid synthesis and fat-storage-inducing TM	18.3	52.13
MRET_2505	Pin2-interacting protein X1	29.96	94.03
MRET_2506	farnesyl diphosphate synthase	53.43	91.38
MRET_2507	omega-amidase	225.57	123.83
MRET_2508	succinate dehydrogenase assembly factor 1	210.49	278.72
MRET_2509	mitochondrial inner membrane protease subunit 2	313.28	240.81
MRET_2510	endopolyphosphatase	86.28	150.52
MRET_2511	isocitrate lyase	488.65	188.01
MRET_2512	DNA-directed RNA polymerase III subunit RPC4	34.72	38.09
MRET_2513	conserved hypothetical protein	807.66	202.3

MRET_2514	growth hormone-inducible transmembrane protein	3309.3	1919.61
MRET_2515	profilin	75.78	65.96
MRET_2516	triosephosphate isomerase (TIM)	672.33	1189.74
MRET_2517	protein PET117	127.81	105.41
MRET_2518	uncharacterized protein	40.14	93.03
MRET_2519	phosphoribosylaminoimidazolecarboxamide formyltransferase/IMP cyclohydrolase	110.47	60.38
MRET_2520	vacuolar fusion protein MON1	13.32	11.46
MRET_2521	DUF890 domain protein	8.63	10.63
MRET_2522	lipoyl synthase	1136.01	849.79
MRET_2523	uncharacterized protein	76.29	77.11
MRET_2524	mitochondrial alcohol dehydrogenase isozyme III	3331.16	2583.11
MRET_2525	DUF202 domain protein	257.77	330.93
MRET_2526	cytochrome c oxidase subunit 17	1256.64	786.61
MRET_2527	large subunit ribosomal protein LP1	148.21	665.83
MRET_2528	trehalase	172.13	139.48
MRET_2529	uncharacterized protein	185.8	75.27
MRET_2530	acetolactate synthase I/III small subunit	203.58	246.23
MRET_2531	oxidoreductase	582.34	436.44
MRET_2532	regulatory subunit of the type I protein phosphatase	970.03	924.69
MRET_2533	AHNAK nucleoprotein	123.51	188
MRET_2534	uncharacterized protein	1705.5	1730.02
MRET_2535	DASH complex subunit DAD4	33.05	102.46
MRET_2536	ribosomal protein S21	20.7	63.6
MRET_2537	uncharacterized protein	204.5	193.14
MRET_2538	protein ROT1	134.77	20.19
MRET_2539	amino-acid N-acetyltransferase	52.31	35.44
MRET_2540	plasma membrane protein involved in remodeling GPI anchors	53.42	37.74
MRET_2541	AP-1 complex subunit sigma 1/2	46.73	91.36
MRET_2542	uncharacterized protein	42.77	64.12
MRET_2543	EKC/KEOPS complex subunit PCC1/LAGE3	219.04	224.02
MRET_2544	ATP-dependent DNA helicase PIF1	165.42	203.81
MRET_2545	translation initiation factor eIF-2B subunit beta	115.6	110.46
MRET_2546	conserved hypothetical protein	64.73	48.32
MRET_2547	origin recognition complex subunit 4	16.6	20.71
MRET_2548	elongator complex protein 4	42.87	59.18
MRET_2549	26S proteasome regulatory subunit T6	165.58	169.8
MRET_2550	mitochondrial import receptor subunit TOM71	23.57	40.45

MRET_2551	tRNA-dihydrouridine synthase 1	10.36	22.85
MRET_2552	sphingosine-1-phosphate phosphohydrolase	111.54	224.46
MRET_2553	NADH dehydrogenase (ubiquinone) 1 alpha subcomplex subunit 3	225.44	206.15
MRET_2554	peptidyl-prolyl isomerase H (cyclophilin H)	90.25	94.68
MRET_2555	para-aminobenzoate (PABA) synthase	58.14	116.46
MRET_2556	uncharacterized protein	80.9	166.89
MRET_2557	uncharacterized protein	2794.26	1774.81
MRET_2558	mitochondrial peroxiredoxin PRX1	145.33	92.55
MRET_2559	short-chain dehydrogenase reductase	40.97	45.6
MRET_2560	calcium permeable stress-gated cation channel	15.24	29.33
MRET_2561	ubiquitin related modifier 1	14.72	14.51
MRET_2562	metal transporter CNNM	23.48	56.33
MRET_2563	uncharacterized protein	79.9	146.03
MRET_2564	cytoplasmic tRNA 2-thiolation protein 1	116.57	93
MRET_2565	ubiquinone biosynthesis protein COQ4	78.48	81.52
MRET_2566	uncharacterized protein	297.52	222.53
MRET_2567	cation diffusion facilitator	128.74	87.02
MRET_2568	3-isopropylmalate dehydrogenase	101.89	118.49
MRET_2569	A transporter	25.44	43.5
MRET_2570	thioesterase	108.79	134.5
MRET_2571	transporter	116.21	41.66
MRET_2572	U4/U6.U5 tri-snRNP-associated protein 2	38.74	51.91
MRET_2573	aspartyl aminopeptidase	181.16	123.99
MRET_2574	zinc finger protein, GATA type	59.77	35.19
MRET_2575	uncharacterized protein	35.87	40.62
MRET_2576	exosome complex component CSL4	73.94	77.19
MRET_2577	protein LTV1	88.25	77.22
MRET_2578	uncharacterized protein	39.93	139.49
MRET_2579	pyruvate dehydrogenase E2 component (dihydrolipoamide acetyltransferase)	880.08	523.93
MRET_2580	RecQ-mediated genome instability protein 1	556.67	320.2
MRET_2581	proteophosphoglycan ppg4	497.89	251.5
MRET_2582	uncharacterized protein	415.21	266.07
MRET_2583	uncharacterized protein	17.37	19.32
MRET_2584	DNA-directed RNA polymerase II subunit RPB2	259.03	255.97
MRET_2585	pre-mRNA-splicing factor CWC25	7.18	18.24
MRET_2586	integral membrane protein (Ptm1)	37.12	88.05
MRET_2587	aminoacyl tRNA synthase complex-interacting multifunctional protein 1	72.8	140.83

MRET_2588	zinc finger protein, C3H1 type	35.74	46.7
MRET_2589	aminomethyltransferase	56.91	99.2
MRET_2590	transporter	53.74	37.49
MRET_2591	nuclear fragile X mental retardation-interacting protein 1 (NUFIP1)	36.74	46.59
MRET_2592	large subunit ribosomal protein L4	35.27	137.61
MRET_2593	small subunit ribosomal protein S16e	105.91	436.13
MRET_2594	SWI/SNF-related matrix-associated actin-dependent regulator of chromatin subfamily D	90.35	104.88
MRET_2595	Ras-related protein Rab-5C	91.13	191.46
MRET_2596	solute carrier family 25 (mitochondrial adenine nucleotide translocator), member 4/5/6/31	3307.35	2297.94
MRET_2597	UBX domain protein 1	966.28	683.79
MRET_2598	Tim17/Tim22/Tim23/Pmp24 family protein	201.13	316.42
MRET_2599	aminopeptidase	423.62	636.06
MRET_2600	aminopeptidase	27.14	32.31
MRET_2601	uncharacterized protein	6.58	10.43
MRET_2602	tRNA acetyltransferase TAN1	12.64	18.13
MRET_2603	8-amino-7-oxononanoate synthase	11.29	10.89
MRET_2604	Vam6/Vps39-like protein vacuolar protein sorting-associated protein 39	66.22	43.7
MRET_2605	uncharacterized protein	202.17	131.96
MRET_2606	uncharacterized protein	26.34	39.35
MRET_2607	RNA-binding protein	75.73	84.21
MRET_2608	chitin synthase	199.81	91.72
MRET_2609	SH3 domain protein	9.49	41.85
MRET_2610	4-aminobutyrate aminotransferase	173.17	289.87
MRET_2611	FH domain protein	17.63	36.81
MRET_2612	cullin 3	64.28	154.81
MRET_2613	serine/threonine-protein kinase MRCK	783.41	630.66
MRET_2614	transcription factor	140.98	151.29
MRET_2615	uncharacterized protein	414.68	406.22
MRET_2616	Hsp70 nucleotide exchange factor FES1	66.72	104.11
MRET_2617	anaphase-promoting complex subunit 6	35	70.13
MRET_2618	A/G-specific adenine glycosylase	31.74	70.26
MRET_2619	uncharacterized protein	11588.3	2892.69
MRET_2620	AP-3 complex subunit sigma	37.06	59.8
MRET_2621	ADP-ribosylation factor-like protein 2	49.15	121.71
MRET_2622	acyl-CoA dehydrogenase	839.54	1062.57
MRET_2623	uncharacterized protein	8.18	20.63
MRET_2624	exosome complex protein LRP1	8.94	15.23

MRET_2625	homeobox domain protein	6.68	22.45
MRET_2626	homeobox domain protein	15.29	10.97
MRET_2627	methionine permease	31.22	88.54
MRET_2628	ATP-dependent RNA helicase DHX29	76.97	56.84
MRET_2629	INO80 complex subunit 1	42.87	59.49
MRET_2630	uncharacterized protein	102.48	89.47
MRET_2631	DNA 5' AMP hydrolase involved in DNA repair	24.15	84.53
MRET_2632	uncharacterized protein	74.12	93.87
MRET_2633	N5-hydroxy-L-ornithine N5-transacylase	80.9	170.79
MRET_2634	L-ornithine N5-monooxygenase	92.99	176.26
MRET_2635	exocyst complex component 1	60.46	77.19
MRET_2636	DUF2305 domain protein	40.46	60.57
MRET_2637	WD repeat protein	47.04	30.93
MRET_2638	aspartate aminotransferase, mitochondrial	205.68	337.46
MRET_2639	DNA polymerase mu	46.9	107.19
MRET_2640	uncharacterized protein	35.89	84.49
MRET_2641	uncharacterized protein	45.01	132.76
MRET_2642	bacterial leucyl aminopeptidase	51.32	189.19
MRET_2643	rRNA biogenesis protein RRP5	77.07	427.21
MRET_2644	Src like adaptor protein	122.86	105.01
MRET_2645	uncharacterized protein	26.51	58.74
MRET_2646	AN1-like zinc finger protein	20.62	38.95
MRET_2647	antiviral helicase SKI2	124.11	136.85
MRET_2648	uncharacterized protein	27.7	35.75
MRET_2649	phosphatidylglycerophosphatase GEP4	18.55	31.1
MRET_2650	transporter (MirC)	19.14	106.54
MRET_2651	uncharacterized protein	45.17	121.87
MRET_2652	plastin-1	43.79	86.83
MRET_2653	low-affinity vacuolar phosphate transporter	110.77	90.39
MRET_2654	solute carrier family 25, member 39/40	266.37	160.5
MRET_2655	tRNA pseudouridine38/39 synthase	198.94	142.26
MRET_2656	SET domain protein	94.76	63.37
MRET_2657	peptidyl-prolyl isomerase E (cyclophilin E)	205.16	192.12
MRET_2658	isopentenyl-diphosphate Delta-isomerase	219.04	309.4
MRET_2659	uncharacterized protein	17.57	145.16
MRET_2660	prephenate dehydratase	15.08	44.16
MRET_2661	secreted aspartic endopeptidase	73.69	100.25

MRET_2662	hydrolase, family 43 protein	74.65	84.43
MRET_2663	GTPase KRas	309.96	539.06
MRET_2664	nucleoside diphosphatase	102.94	92.8
MRET_2665	exocyst complex component 3	13.65	17.18
MRET_2666	CCCH finger DNA binding protein	6.57	46.29
MRET_2667	outer membrane protein TOM13	0.7	15.12
MRET_2668	uncharacterized protein	5.28	78.32
MRET_2669	phosphoribosylaminoimidazole carboxylase	256.96	126.61
MRET_2670	choline oxidase	210.02	200.08
MRET_2671	uncharacterized protein	262.74	179.37
MRET_2672	E3 ubiquitin-protein ligase MARCH6	34.71	48.45
MRET_2673	aromatic-L-amino-acid/L-tryptophan decarboxylase	22.45	46.79
MRET_2674	nuclear pore complex protein Nup205	62.67	45.39
MRET_2675	uncharacterized protein	9.35	14.38
MRET_2676	DUF2315 domain protein	31.45	58.83
MRET_2677	DNA-directed RNA polymerase III subunit RPC7	127.46	126.34
MRET_2678	stress-induced-phosphoprotein 1	568.05	1032.79
MRET_2679	uncharacterized protein	42.48	73.64
MRET_2680	cleavage stimulation factor subunit 3	24.99	52.69
MRET_2681	mitochondrial protein sorting (Msf1)	119.93	108.1
MRET_2682	uncharacterized protein	66.33	48.43
MRET_2683	uncharacterized protein	32.16	70.54
MRET_2684	U3 small nucleolar RNA-associated protein 5	30.3	49.12
MRET_2685	negative regulator of the PHO system	168.12	374.12
MRET_2686	cell division cycle 20, cofactor of APC complex	42.23	186.18
MRET_2687	glucose transporter	34.28	31.71
MRET_2688	ferredoxin-2, mitochondrial	217.8	163.68
MRET_2689	DEAD/DEAH box helicase	163.31	100.14
MRET_2690	beta-glucan synthesis-associated protein KRE6	61.31	120.33
MRET_2691	cellular nucleic acid-binding protein	2038.08	1909.38
MRET_2692	signal recognition particle subunit SRP19	87.42	161.24
MRET_2693	prolyl-tRNA synthetase	26.28	54
MRET_2694	seryl-tRNA synthetase	54.48	103.31
MRET_2695	DnaJ homolog subfamily C member 2	60.62	102.55
MRET_2696	ribonuclease h-like protein	6.71	24.83
MRET_2697	glycosyl hydrolases family 8	50.19	105.7
MRET_2698	small subunit ribosomal protein S17e	77.56	326.45

MRET_2699	small subunit ribosomal protein S15Ae	151.34	666.55
MRET_2700	THO complex subunit 7	66.56	157.68
MRET_2701	large subunit ribosomal protein L14e	97.2	545.24
MRET_2702	inositol-hexakisphosphate 5-kinase	100.48	78.84
MRET_2703	serine/threonine-protein kinase	84.62	80.78
MRET_2704	uncharacterized protein	44.97	61.59
MRET_2705	uncharacterized protein	119.63	77.6
MRET_2706	mitochondrial-processing peptidase subunit alpha	427.14	363.6
MRET_2707	PHD finger domain protein	10.05	22.44
MRET_2708	uncharacterized protein	11.48	40.35
MRET_2709	ubiquinol-cytochrome c reductase subunit 9	262.25	371.17
MRET_2710	tether containing UBX domain for GLUT4	25.99	59.35
MRET_2711	synaptojanin	193.01	85.4
MRET_2712	NADH dehydrogenase	249.57	192.47
MRET_2713	amino acid transporter	93.31	67.48
MRET_2714	oxidoreductase	2411.13	708.94
MRET_2715	arp2 3 complex 34 kda subunit	25.68	26.08
MRET_2716	uncharacterized protein	121.72	108.02
MRET_2717	heat shock factor-binding protein 1	127.36	130.45
MRET_2718	CORD and CS domain protein	647.58	405.81
MRET_2719	uncharacterized protein	50.58	81.1
MRET_2720	COMPASS component SWD2	22.06	44.34
MRET_2721	enoyl-CoA hydratase/isomerase family	88.97	68.9
MRET_2722	enoyl-CoA hydratase/isomerase family	145.67	103.03
MRET_2723	leucine-rich repeat protein	219.27	140.71
MRET_2724	uncharacterized protein	46.78	50.58
MRET_2725	large subunit ribosomal protein LP0	333.49	763.31
MRET_2726	uncharacterized protein	330.48	175.01
MRET_2727	glutamate decarboxylase	140.98	187.07
MRET_2728	DNA-(apurinic or apyrimidinic site) lyase	32.85	71.79
MRET_2729	chromosome transmission fidelity protein 18	28.17	33.13
MRET_2730	U5 small nuclear ribonucleoprotein component	36.91	41.75
MRET_2731	solute carrier family 45, member 1/2/4	8.89	23.09
MRET_2732	GTP-binding protein	26.94	92.86
MRET_2733	uncharacterized protein	29.76	57.58
MRET_2734	bloom syndrome protein	102.33	74.64
MRET_2735	6-phosphogluconate dehydrogenase	2015.57	1584.63

MRET_2736	small glutamine-rich tetratricopeptide repeat protein alpha	117.45	182.75
MRET_2737	sterol-sensing domain of SREBP cleavage-activation protein	117.93	109.51
MRET_2738	cohesin complex subunit SCC1	95.06	42.93
MRET_2739	solute carrier family 38 (sodium-coupled neutral amino acid transporter), member 11	70.77	65.34
MRET_2740	GTPase-activating protein	22.73	32.06
MRET_2741	F-box protein 9	15.51	18.71
MRET_2742	transcription factor	248.67	174.88
MRET_2743	actin cytoskeleton-regulatory complex protein SLA1	279.54	209.26
MRET_2744	START domain protein	249.32	150.37
MRET_2745	protein MAK16	48.22	99.77
MRET_2746	TKL protein kinase	123.99	130.1
MRET_2747	uncharacterized protein	4997.78	1717.44
MRET_2748	uncharacterized protein	130.65	121.74
MRET_2749	uncharacterized protein	467.99	196.26
MRET_2750	cyclin-dependent protein kinase regulator Pho80	86.69	244.13
MRET_2751	pre-mRNA-splicing factor 18	157.07	146.82
MRET_2752	cytochrome c oxidase subunit 6b	1422.35	1176.63
MRET_2753	tRNA (uracil-5-)-methyltransferase TRM9	38.06	65.8
MRET_2754	chitin binding peritrophin-A domain protein	538.27	304.38
MRET_2755	diacylglycerol kinase catalytic domain protein	176.73	95.16
MRET_2756	conserved hypothetical protein	60.66	39.89
MRET_2757	uncharacterized protein	336.18	268.73
MRET_2758	T-complex protein 1 subunit delta	46.81	81.68
MRET_2759	ubiquitin fusion degradation protein 1	525.48	499.91
MRET_2760	demethylmenaquinone methyltransferase	703.23	358.15
MRET_2761	Csr1-phosphatidylinositol transfer protein	143.59	145.57
MRET_2762	zinc finger protein, C2H2 type	525.59	442.72
MRET_2763	L-methionine (R)-S-oxide reductase	32.62	83.87
MRET_2764	homoserine O-acetyltransferase	59.45	151.52
MRET_2765	histidyl-tRNA synthetase	174.39	157.1
MRET_2766	membrane fusion protein Use1	24.37	49.07
MRET_2767	2-iminobutanoate/2-iminopropanoate deaminase	170.86	183.1
MRET_2768	RhoGEF domain protein	60.07	55.92
MRET_2769	NADH dehydrogenase (ubiquinone) Fe-S protein 5	335.61	299.85
MRET_2770	phosphatidylinositol glycan, class Q	46.79	35.08
MRET_2771	Ras-related protein Rab-6A	182.32	252.41
MRET_2772	seipin	35.39	41.01

MRET_2773	immunoglobulin-binding protein 1	32.03	48.72
MRET_2774	sphingosine kinase	86.53	51.78
MRET_2775	protein disulfide-isomerase A6	357.31	274.3
MRET_2776	uncharacterized protein	7.91	13.38
MRET_2777	urease	25.92	38.5
MRET_2778	ubiquitin-like protein Nedd8	33.46	53.35
MRET_2779	peroxiredoxin Q/BCP	77.81	60.7
MRET_2780	ESCRT-I complex subunit TSG101	90.77	60.46
MRET_2781	ATP-dependent RNA helicase MRH4, mitochondrial	36.38	22.17
MRET_2782	exopolyphosphatase	228.63	189.58
MRET_2783	tyrosine-protein phosphatase SIW14	195.12	122.67
MRET_2784	YagE family protein	222.93	128.79
MRET_2785	vacuolar protein sorting-associated protein 54	46.4	39.24
MRET_2786	BTB domain and ankyrin repeat protein	15.98	37.68
MRET_2787	uncharacterized protein	26.76	60.16
MRET_2788	Obg-like ATPase 1	129.17	432.44
MRET_2789	uncharacterized protein	1939.8	1255.73
MRET_2790	ribonuclease H2 subunit A	56.95	74.65
MRET_2791	exosome complex exonuclease RRP6	72.53	97.58
MRET_2792	cellulase (glycosyl hydrolase family 5)	131.54	176.95
MRET_2793	cellulase (glycosyl hydrolase family 5)	140.67	135.47
MRET_2794	ATP-dependent RNA helicase	135.25	179.84
MRET_2795	splicing factor 3A subunit 3	46.71	90.56
MRET_2796	transcription regulator staf-5 like protein	582.94	452.36
MRET_2797	uncharacterized protein	62.92	164.76
MRET_2798	ribonuclease T2	298.02	231.64
MRET_2799	integrase	23.21	29.58
MRET_2800	DNA-directed RNA polymerase I subunit RPA1	199.55	124.88
MRET_2801	tRNA threonylcarbamoyladenosine dehydratase	189.07	206.85
MRET_2802	6-phosphofructo-2-kinase/fructose-2,6-biphosphatase 2	52.5	71.08
MRET_2803	uncharacterized protein	23.86	38.27
MRET_2804	methionyl aminopeptidase	73.18	101.64
MRET_2805	ubiquitin-conjugating enzyme E2 M	164.75	52.03
MRET_2806	thiosulfate/3-mercaptopyruvate sulfurtransferase	1054.74	454.2
MRET_2807	arrestin-related trafficking adapter 3/6	154.74	300.4
MRET_2808	nuclear pore complex protein Nup188	37.2	38.5
MRET_2809	conserved oligomeric golgi complex subunit 6	135.57	107.12

MRET_2810	uncharacterized protein	838.47	491.26
MRET_2811	mitochondrial genome maintenance protein MGR2	793.17	659.51
MRET_2812	methyltransferase-like protein 6	362.39	341.88
MRET_2813	nuclear pore complex protein Nup133	95.9	55.96
MRET_2814	protein of unknown function (DUF1682)	87.83	95.51
MRET_2815	ubiquitin carboxyl-terminal hydrolase 7	435.79	204.16
MRET_2816	chalcone-flavanone isomerase	1430.4	589.45
MRET_2817	glucosamine-phosphate N-acetyltransferase	59.54	48.72
MRET_2818	alpha/beta-hydrolase	41	59.99
MRET_2819	uncharacterized protein	47.23	70.65
MRET_2820	uncharacterized protein	93.05	126.81
MRET_2821	Taurine dioxygenase	40.91	57.21
MRET_2822	eukaryotic aspartyl protease	108.27	129.89
MRET_2823	uncharacterized protein	106.66	199.96
MRET_2824	uncharacterized protein	463.44	1914.71
MRET_2825	secreted aspartic endopeptidase	144.85	197.09
MRET_2826	lipase precursor-like protein	6.28	12.66
MRET_2827	eukaryotic aspartyl protease	0	0
MRET_2828	uncharacterized protein	21.27	109.47
MRET_2829	uncharacterized protein	23.94	55.73
MRET_2830	MFS monocarboxylate transporter	10.01	46.96
MRET_2831	phenylalanyl-tRNA synthetase beta chain	43.7	98.22
MRET_2832	electron-transferring-flavoprotein dehydrogenase	124.35	57.61
MRET_2833	Rare lipoprotein A (RlpA)-like double-psi beta-barrel	68.51	82.44
MRET_2834	prefoldin beta subunit	20.35	69.22
MRET_2835	rabenosyn-5	23.4	46.41
MRET_2836	carboxyl methyltransferase	8.64	15.74
MRET_2837	5-methyltetrahydropteroyltriglutamate--homocysteine methyltransferase	432.86	862.01
MRET_2838	uncharacterized protein	162.77	1805.05
MRET_2839	uncharacterized protein	633.64	1069.7
MRET_2840	protein YOP1	117.85	55.01
MRET_2841	protein-tyrosine phosphatase	18.63	18.48
MRET_2842	exocyst complex component 7	18.17	23.68
MRET_2843	uncharacterized protein	75.48	135.31
MRET_2844	verprolin, proline-rich actin-associated protein	417.27	213.43
MRET_2845	translation initiation factor 4E	80.17	159.83
MRET_2846	serine/threonine-protein kinase	140.98	210.71

MRET_2847	porphobilinogen synthase	521.68	556.63
MRET_2848	protein FET5	70.46	137.76
MRET_2849	protein CWC15	62.7	118.79
MRET_2850	PCI domain 2 protein	20.26	47.8
MRET_2851	tRNA (guanine9-N1)-methyltransferase	76.22	108.01
MRET_2852	serine/threonine-protein kinase	193.7	319.95
MRET_2853	glycine dehydrogenase	124.91	212.67
MRET_2854	hexokinase	793.74	626.67
MRET_2855	UDP-galactopyranose mutase	123.88	235.02
MRET_2856	large subunit ribosomal protein L44e	573.36	959.43
MRET_2857	serine/threonine-protein kinase BUR1	105.12	118.08
MRET_2858	pre-mRNA-processing factor SLU7	513.84	468.91
MRET_2859	ribosome recycling factor	827.58	319.47
MRET_2860	transcription initiation factor TFIID subunit 9B	141.14	251.54
MRET_2861	coatomer subunit delta	107.93	241.74
MRET_2862	20S proteasome subunit beta 4	186.89	349.25
MRET_2863	kinetochore protein Mis12/MTW1	8.22	27.42
MRET_2864	splicing factor 1	61.01	81.71
MRET_2865	uncharacterized protein	124.9	375.47
MRET_2866	diacylglycerol kinase (CTP)	57.23	227.85
MRET_2867	uncharacterized protein	15.09	55.84
MRET_2868	pre-mRNA-splicing factor clf1	10.17	34.2
MRET_2869	copper ion binding protein	21.73	46.4
MRET_2870	eukaryotic sulfide quinone oxidoreductase	434.16	319.71
MRET_2871	transporter	32.88	58.36
MRET_2872	mitochondrial genome maintenance protein MGM101	27.86	74.95
MRET_2873	ATP-dependent RNA helicase DDX47/RRP3	18.65	34.89
MRET_2874	serine/threonine-protein kinase	585.13	364.52
MRET_2875	mitochondrial inner membrane	195.41	171.17
MRET_2876	amino acid permease	71.08	52.39
MRET_2877	uncharacterized protein	273.02	236.81
MRET_2878	uncharacterized protein	157.66	66.85
MRET_2879	PH domain protein	27.44	55.05
MRET_2880	small subunit ribosomal protein S12	117.38	140.27
MRET_2881	uncharacterized protein	2.68	10.53
MRET_2882	elongation factor 1-beta	206.68	375.24
MRET_2883	PHD finger domain protein	97.07	136

MRET_2884	peptidyl-prolyl cis-trans isomerase	1546.97	1538.58
MRET_2885	nuclear transport factor	229.76	432.88
MRET_2886	aspartic-type endopeptidase CTSD	2613.11	7402.24
MRET_2887	uncharacterized protein	62.17	154.12
MRET_2888	transcription initiation factor TFIID subunit 2	520.79	193.75
MRET_2889	mediator of RNA polymerase II transcription subunit 7	56.12	115.12
MRET_2890	DNA replication ATP-dependent helicase DNA2	13.06	11.02
MRET_2891	amino acid transporter	47.02	42.26
MRET_2892	amino acid transporter	21.81	95.37
MRET_2893	amino acid transporter	39.11	56.97
MRET_2894	amino acid transporter	319.66	190.61
MRET_2895	SNW domain protein 1	54.29	461.32
MRET_2896	DNA polymerase eta	0.14	43.88
MRET_2897	coproporphyrinogen III oxidase	398.55	278.6
MRET_2898	conserved hypothetical protein	67.32	28.43
MRET_2899	3-phosphoinositide dependent protein kinase-1	65.99	56.83
MRET_2900	AHNAK nucleoprotein	54.91	78.58
MRET_2901	HIV Tat-specific factor 1	264.36	372.57
MRET_2902	hexaprenyl-diphosphate synthase	172.41	167.2
MRET_2903	peptidyl-tRNA hydrolase, PTH1 family	37.56	16.98
MRET_2904	PAB1 binding protein	166.37	174.45
MRET_2905	uncharacterized protein	122.14	112.02
MRET_2906	mRNA splicing protein	19.42	33.57
MRET_2907	kynureninase	36.76	56.41
MRET_2908	D-tyrosyl-tRNA(Tyr) deacylase	11.34	30.79
MRET_2909	RAD51-like protein 2	138.24	135.73
MRET_2910	myo-inositol-1(or 4)-monophosphatase	78.37	78.92
MRET_2911	nuclear pore complex protein Nup54	42.53	53.42
MRET_2912	serine dehydratase beta chain	198.16	148.34
MRET_2913	uncharacterized protein	40.06	51.11
MRET_2914	dehydrogenase	149.17	115.87
MRET_2915	uncharacterized protein	175.29	110.05
MRET_2916	chromatin assembly factor 1 subunit B	204.59	118.08
MRET_2917	UBX domain protein	75.56	92.34
MRET_2918	cell division control protein	77.53	46.74
MRET_2919	RNA exonuclease NGL2	15.09	27.53
MRET_2920	tRNA (cytidine32/guanosine34-2'-O)-methyltransferase	13.24	23.52

MRET_2921	tRNA (adenine57-N1/adenine58-N1)-methyltransferase catalytic subunit	42.27	62.05
MRET_2922	ribosome biogenesis protein MAK21	32.2	135.09
MRET_2923	transcription elongation factor S-II	38.38	131.99
MRET_2924	signal peptidase complex subunit 1	108.81	151.24
MRET_2925	DnaJ domain protein	23.81	55.43
MRET_2926	amyloid beta precursor protein binding protein 1	40.41	79.68
MRET_2927	nucleolar pre-ribosomal-associated protein 1	163.13	55.05
MRET_2928	uncharacterized protein	75.34	45.67
MRET_2929	ubiquitin carboxyl-terminal hydrolase 22/27/51	101.04	82.71
MRET_2930	protein of unknown function (DUF2424)	128.04	124.43
MRET_2931	phosphatidylinositol glycan, class C	222.37	180.82
MRET_2932	zinc finger protein, C2H2 type	1196.26	702.43
MRET_2933	monomeric glyoxalase I	453.37	339.15
MRET_2934	tyrosyl-tRNA synthetase	55.77	108.25
MRET_2935	endosomal cargo receptor (Erp3)	287.12	402.26
MRET_2936	solute carrier family 29 (equilibrative nucleoside transporter), member 1/2/3	46.75	60.97
MRET_2937	NADH-ubiquinone oxidoreductase 9.5 kDa subunit	74.09	143.82
MRET_2938	uncharacterized protein	31.7	107.33
MRET_2939	uncharacterized protein	51.9	85.53
MRET_2940	protein transport protein SEC13	195.38	344.28
MRET_2941	pre-mRNA-splicing factor ATP-dependent RNA helicase DHX15/PRP43	195.33	223.22
MRET_2942	nuclear pore complex protein Nup85	89.62	131.11
MRET_2943	large subunit ribosomal protein L23e	143.81	477.54
MRET_2944	GTP cyclohydrolase IA	40.82	109.2
MRET_2945	cytochrome b5	1411.17	776.87
MRET_2946	serine/threonine-protein kinase KIN1/2	166	150
MRET_2947	T-complex protein 1 subunit zeta	56.81	94.22
MRET_2948	WD repeat protein 48	53.55	70.96
MRET_2949	actin cytoskeleton-regulatory complex protein PAN1	413.25	182.08
MRET_2950	non-classical export protein 1	136.25	164.13
MRET_2951	chitin synthase	9.76	30.31
MRET_2952	phosphatase domain, paladin 1	285.58	96.9
MRET_2953	protein of unknown function (DUF1749)	371.18	312.18
MRET_2954	large subunit ribosomal protein L17e	249.66	258.58
MRET_2955	vesicle-associated membrane protein 4	944.45	790.66
MRET_2956	2-oxoglutarate dehydrogenase E1 component	2324.26	1051.01
MRET_2957	glutamate dehydrogenase	26.99	41.11

MRET_2958	nucleolar protein 4	43.06	50.65
MRET_2959	uncharacterized protein	559.95	432.4
MRET_2960	chromatin modification-related protein EAF6	328.91	220.31
MRET_2961	CTD kinase subunit gamma	45.48	109.05
MRET_2962	cytosolic iron-sulfur protein assembly protein 1	42.35	80.74
MRET_2963	ubiquinone biosynthesis protein COQ9	49.05	121.98
MRET_2964	Ca2+-transporting ATPase	304.95	215.51
MRET_2965	uncharacterized protein	169.12	236.03
MRET_2966	fungal specific transcription factor domain protein	95	89.38
MRET_2967	signal recognition particle subunit SRP68	25.87	47.23
MRET_2968	septum formation protein	19.68	61.45
MRET_2969	small subunit ribosomal protein S23	21.8	66.84
MRET_2970	D-3-phosphoglycerate dehydrogenase/2-oxoglutarate reductase	11.21	51.08
MRET_2971	cell division control protein 11	81.11	242.89
MRET_2972	chaperone	27.39	72.1
MRET_2973	uncharacterized protein	149.6	184.83
MRET_2974	uncharacterized protein	769.63	947.77
MRET_2975	NADH-dependent flavin oxidoreductase	1468.25	3602.99
MRET_2976	uncharacterized protein	66.05	78.51
MRET_2977	parafibromin	87.95	130.13
MRET_2978	histone H3-like centromeric protein A	123.82	122.48
MRET_2979	GTI1/PAC2 family transcription factor	79.7	53.47
MRET_2980	uncharacterized protein	70.34	129.17
MRET_2981	mitochondrial ribosomal subunit S27	35.73	146.08
MRET_2982	chorismate synthase	71.25	140.33
MRET_2983	ADP-ribosylation factor GTPase-activating protein 2/3	96.77	118.91
MRET_2984	CCR4-NOT transcription complex subunit 2	50.2	90.47
MRET_2985	uncharacterized protein	350.14	348.46
MRET_2986	serum/glucocorticoid-regulated kinase 2	169.75	149.07
MRET_2987	DNA-directed RNA polymerases I and III subunit RPAC2	92.31	204.03
MRET_2988	quinone oxidoreductase	309.76	243.51
MRET_2989	phosphoglucomutase	34	40.24
MRET_2990	tRNA-splicing endonuclease subunit Sen34	37.55	58.55
MRET_2991	phosphatidylinositol 3-kinase	6.11	13.94
MRET_2992	alpha-1,2-mannosyltransferase	22.87	44.78
MRET_2993	electron transfer flavoprotein beta subunit	144.31	181.1
MRET_2994	DNA polymerase alpha-associated DNA helicase A	54.85	75.24

MRET_2995	neuronal calcium sensor 1	978.01	882.24
MRET_2996	anthranilate phosphoribosyltransferase	15.97	36.51
MRET_2997	secretory pathway protein Ssp120	668.97	511.93
MRET_2998	ATP-binding cassette, subfamily B (MDR/TAP), member 1	220.68	204.45
MRET_2999	phosphoribosylglycinamide formyltransferase	42.79	58.2
MRET_3000	mitofusin 2	1326.63	840.83
MRET_3001	uncharacterized protein	122.51	189.12
MRET_3002	large subunit ribosomal protein L23Ae	116.86	469.52
MRET_3003	uncharacterized protein	51.91	74.5
MRET_3004	transcription factor	30.89	41.85
MRET_3005	activating transcription factor 7 interacting protein	8.05	43.24
MRET_3006	exosome complex component RRP42	53.14	67.95
MRET_3007	3-isopropylmalate dehydratase	34.23	47.58
MRET_3008	V-type H+-transporting ATPase 16kDa proteolipid subunit	407.47	435.6
MRET_3009	mitochondrial-processing peptidase subunit beta	2522.49	1644.49
MRET_3010	zinc finger protein, C3HC4 type (RING finger)	99.62	79.07
MRET_3011	mediator of RNA polymerase II transcription subunit 6	16.05	21.15
MRET_3012	uncharacterized protein	142	85.7
MRET_3013	uncharacterized protein	59.99	63.87
MRET_3014	signal peptidase I	66.92	108.75
MRET_3015	uncharacterized protein	37.71	73.51
MRET_3016	poly(A) polymerase	105.89	143.57
MRET_3017	S2P endopeptidase	32.02	18.33
MRET_3018	epsin	181.85	357.32
MRET_3019	20S proteasome subunit alpha 5	157.48	170.61
MRET_3020	trafficking protein particle complex subunit 10	27.2	31.35
MRET_3021	RNA polymerase II-associated protein 1	53.08	37.04
MRET_3022	transcription initiation factor TFIID subunit 13	44.66	54.09
MRET_3023	glutathione reductase (NADPH)	912.18	443.07
MRET_3024	Indole-diterpene biosynthesis protein PaxU	76.98	101.53
MRET_3025	WD repeat protein	31.66	56.69
MRET_3026	dephospho-CoA kinase	348.92	235.48
MRET_3027	transcription initiation factor TFIID subunit 12	70.69	145.22
MRET_3028	release factor glutamine methyltransferase	68.9	144.93
MRET_3029	U3 small nucleolar RNA-associated protein 19	79.84	122.81
MRET_3030	uncharacterized protein	76.58	78.71
MRET_3031	U3 small nucleolar RNA-associated protein 18	81.09	107.25

MRET_3032	hydroxymethylbilane synthase	56.35	65.76
MRET_3033	serine/threonine-protein kinase ATR	10.74	17.5
MRET_3034	oxysterol-binding protein-related protein 9/10/11	81.03	191.54
MRET_3035	U3 small nucleolar RNA-associated protein 12	83.75	67.66
MRET_3036	tubulin-tyrosine ligase family protein	156.07	147.94
MRET_3037	small subunit ribosomal protein S8e	453.31	911.33
MRET_3038	bZIP transcription factor	1373.51	1070.23
MRET_3039	GTPase-activating protein	46.11	100.35
MRET_3040	oxysterol-binding protein-related protein 3/6/7	303.43	522
MRET_3041	cellular morphogenesis regulator DopA	20.66	37.34
MRET_3042	exocyst complex component 8	122.17	71.83
MRET_3043	signal transduction protein Syg1	29.45	29.78
MRET_3044	small subunit ribosomal protein S15	72.03	119.32
MRET_3045	ubiquitin-protein ligase E3 D	180.52	135.79
MRET_3046	protein SHQ1	31.17	51.93
MRET_3047	WD repeat protein	331.13	192.24
MRET_3048	chitinase	195.24	157.42
MRET_3049	putative integral membrane protein that interacts with Rpp0p	21.71	37.56
MRET_3050	signal recognition particle subunit SRP14	67.33	118.69
MRET_3051	auxin efflux carrier	69.47	81.72
MRET_3052	AP-2 complex-associated kinase	23.83	28.71
MRET_3053	ribosomal large subunit biogenesis	55.5	62.54
MRET_3054	structure-specific endonuclease subunit SLX1	64.2	37.5
MRET_3055	conserved hypothetical protein	573.17	608.66
MRET_3056	ER membrane protein complex subunit 2	116.45	207.93
MRET_3057	serine/threonine-protein phosphatase PP1 catalytic subunit	603.52	1150.59
MRET_3058	Wwm1-ww domain containing protein interacting with metacaspase	1436.23	903.16
MRET_3059	polyribonucleotide 5'-hydroxyl-kinase	55.25	96.07
MRET_3060	CD2 antigen cytoplasmic tail-binding protein 2	10.26	21.54
MRET_3061	NADH dehydrogenase (ubiquinone) 1 alpha subcomplex subunit 2	1084.54	839.88
MRET_3062	transketolase	391.69	286.53
MRET_3063	2-isopropylmalate synthase	53.49	67.32
MRET_3064	uncharacterized protein	300.37	247.39
MRET_3065	SET domain protein	68.99	151.92
MRET_3066	peroxin-19	152.17	195.67
MRET_3067	palmitoyltransferase ZDHHC6	104.69	79.01
MRET_3068	myosin I	104.98	69.15

MRET_3069	acyl-protein thioesterase responsible for depalmitoylation of Gpa1p	61.26	85.04
MRET_3070	fungal Zn(2)-Cys(6) binuclear cluster domain protein	17.56	39.37
MRET_3071	uncharacterized protein	97.69	198.72
MRET_3072	large subunit ribosomal protein L34e	223.91	715.03
MRET_3073	large subunit ribosomal protein L6	57.73	120.35
MRET_3074	E3 ubiquitin-protein ligase synoviolin	666.67	397.87
MRET_3075	NAD+ kinase	59.04	58.9
MRET_3076	zinc finger protein	485.5	444.11
MRET_3077	uncharacterized protein	195.75	216.27
MRET_3078	uncharacterized protein	35.91	38.11
MRET_3079	uncharacterized protein	76.93	228.8
MRET_3080	phosducin family	81.55	220.27
MRET_3081	uncharacterized protein	118.51	305.45
MRET_3082	glucan synthesis regulatory protein	158.07	109.71
MRET_3083	mannosyl-oligosaccharide glucosidase	98.45	86.65
MRET_3084	dynamitin	15.06	13.82
MRET_3085	RNA-binding protein	22.14	39.31
MRET_3086	RAM signalling pathway protein domain protein	47.61	22.83
MRET_3087	transcription elongation factor SPT6	33.56	56.24
MRET_3088	phospholipase C	18.99	81.7
MRET_3089	histone-lysine N-methyltransferase SUV420H	335	169.95
MRET_3090	26S proteasome regulatory subunit T3	81.79	98.07
MRET_3091	pyrroline-5-carboxylate reductase	56	63.58
MRET_3092	uncharacterized protein	12.45	16.49
MRET_3093	endosomal peripheral membrane protein	20.45	21.52
MRET_3094	cytochrome p450	41.65	73.71
MRET_3095	Yqey-like protein	33.49	27.89
MRET_3096	F-box and WD-40 domain protein MET30	517.83	552.99
MRET_3097	large subunit ribosomal protein L26e	104.79	344.67
MRET_3098	GTP-binding protein SAR1	290.34	606.68
MRET_3099	glycogen synthase kinase 3 beta	390.87	415.51
MRET_3100	urease accessory protein	88.89	157.35
MRET_3101	U3 small nucleolar RNA-associated protein 24	183.66	311.2
MRET_3102	translation initiation factor 1	520.92	1159.73
MRET_3103	conserved hypothetical protein	79.45	107.62
MRET_3104	uncharacterized protein	11.64	49.07
MRET_3105	alpha-1,3/alpha-1,6-mannosyltransferase	87.1	51.87

MRET_3106	mitochondrial fusion and transport protein UGO1	156.98	124.04
MRET_3107	multisite-specific tRNA:(cytosine-C5)-methyltransferase	242.1	140.9
MRET_3108	molecular chaperone DnaJ	1416.81	1074.07
MRET_3109	low molecular weight phosphotyrosine protein phosphatase	57.67	130.41
MRET_3110	Sec1 family domain protein 1	42.55	77.89
MRET_3111	acyl-CoA thioesterase 8	99.37	121.88
MRET_3112	JmjC domain, hydroxylase	216.05	125.84
MRET_3113	GYF domain protein	25.3	29.34
MRET_3114	histone deacetylase 1/2	78.97	147.79
MRET_3115	V-type H+-transporting ATPase subunit F	103.82	156.31
MRET_3116	ubiquitin C	5358.25	4367.52
MRET_3117	peptidyl-tRNA hydrolase, PTH2 family	72.98	158.13
MRET_3118	mRNA-decapping enzyme subunit 2	96.44	161.16
MRET_3119	biotin synthase	428.49	389.08
MRET_3120	uncharacterized protein	200.61	109.2
MRET_3121	dynein heavy chain 1, cytosolic	59.68	40.69
MRET_3122	component of the septin ring that is required for cytokinesis	63.99	166.09
MRET_3123	U2 small nuclear ribonucleoprotein A'	11.01	39.76
MRET_3124	dynein heavy chain 1, cytosolic	19.57	54.74
MRET_3125	uncharacterized protein	25.84	40.36
MRET_3126	CCAAT-binding transcription factor subunit HAPB	186.89	239.14
MRET_3127	vacuolar protein sorting-associated protein 13A/C	285.54	274.52
MRET_3128	large subunit ribosomal protein L47	49.49	126.66
MRET_3129	metal iron transporter	40.13	85.97
MRET_3130	AHNAK nucleoprotein	20.12	82.91
MRET_3131	polyketide synthase	54.84	143.57
MRET_3132	nuclear pore complex protein Nup98-Nup96	92.24	78.39
MRET_3133	uncharacterized protein	361.99	196.27
MRET_3134	molecular chaperone HscB	69.92	71.18
MRET_3135	protein phosphatase PTC7	519	404.68
MRET_3136	Kelch repeats protein	69.87	148.57
MRET_3137	endoplasmic reticulum-golgi intermediate compartment protein 2	316.34	365.35
MRET_3138	U6 snRNA-associated Sm-like protein LSm7	83.81	110.69
MRET_3139	protein transport protein DSL1/ZW10	36.39	42.44
MRET_3140	serine/threonine-protein phosphatase 2A regulatory subunit B	66.95	132.42
MRET_3141	prolyl oligopeptidase	78.21	60.37
MRET_3142	solute carrier family 31 (copper transporter), member 1	525.48	213.66

MRET_3143	F-box and WD-40 domain protein CDC4	38.09	33.51
MRET_3144	DnaJ homolog subfamily C member 7	18.38	47.92
MRET_3145	tRNA pseudouridine38-40 synthase	88.2	85.57
MRET_3146	RNA polymerase-associated protein RTF1	43.25	46.75
MRET_3147	actin-related protein 2	740.44	686.24
MRET_3148	aarF domain kinase	75.61	143.49
MRET_3149	cytochrome c oxidase subunit 5a	550.33	601.09
MRET_3150	E3 ubiquitin-protein ligase SHPRH	91.54	45.99
MRET_3151	Myb-like DNA-binding domain protein	154.66	122.56
MRET_3152	RNA recognition motif domain protein	8153.67	2236.21
MRET_3153	Ras-related protein Rab-5C	715.52	417.66
MRET_3154	pantoate--beta-alanine ligase	156.69	117.81
MRET_3155	methionyl aminopeptidase	286.73	364.1
MRET_3156	cytochrome c oxidase subunit 7	197.36	254.07
MRET_3157	oxidoreductase which may be involved in DNA replication (By similarity)	49.3	53.5
MRET_3158	elongation factor 1-gamma	236.18	385.39
MRET_3159	20S proteasome subunit alpha 3	81.7	139.94
MRET_3160	small nuclear ribonucleoprotein D3	59.73	170.59
MRET_3161	high-mobility group non-histone chromatin protein	313.58	523.01
MRET_3162	solute carrier family 25 (mitochondrial citrate transporter), member 1	19.19	32.81
MRET_3163	chloride channel	103.98	60.28
MRET_3164	multiple RNA-binding domain protein 1	153.87	143.65
MRET_3165	mediator of RNA polymerase II transcription subunit 10	46.97	38.65
MRET_3166	26S proteasome regulatory subunit N3	51.56	69.19
MRET_3167	uncharacterized protein	163.42	219.26
MRET_3168	cysteine and glycine-rich protein	133.18	102.74
MRET_3169	choline-phosphate cytidylyltransferase	8.35	54.63
MRET_3170	NADH dehydrogenase (ubiquinone) 1 alpha subcomplex subunit 6	175.38	278.94
MRET_3171	serine/threonine-protein phosphatase 6 catalytic subunit	33.66	72.84
MRET_3172	uncharacterized protein	59.76	63.54
MRET_3173	dihydrolipoamide dehydrogenase	3758.82	1658.61
MRET_3174	mRNA m6A methyltransferase	158.68	95.44
MRET_3175	BAP31 domain protein	877.06	402.62
MRET_3176	DUF89 domain protein	270.97	174.99
MRET_3177	NGG1 interacting factor	253.75	167.62
MRET_3178	DNA-directed RNA polymerase III subunit RPC2	92.82	38.81
MRET_3179	protein of unknown function (DUF3128)	392.04	880.58

MRET_3180	Mago binding protein	51.1	174.85
MRET_3181	large subunit ribosomal protein L28e	96.17	339.27
MRET_3182	prefoldin subunit	15.94	35.16
MRET_3183	translation initiation factor IF-2	101.89	58.23
MRET_3184	ubiquitin-conjugating enzyme E2 G1	224.49	185.5
MRET_3185	CCR4-NOT transcription complex subunit 7/8	45.64	80.95
MRET_3186	26S proteasome regulatory subunit N10	89.38	155.17
MRET_3187	pheromone a factor receptor	124.4	0
MRET_3188	exosome complex component RRP41	64.09	117.05
MRET_3189	glyceraldehyde 3-phosphate dehydrogenase	3328.35	1924.7
MRET_3190	cell division control protein 24	194.92	115.98
MRET_3191	protein disulfide-isomerase A1	1107.8	802.57
MRET_3192	L-gulonolactone oxidase	140.36	66.72
MRET_3193	26S proteasome regulatory subunit N9	30.29	37.78
MRET_3194	phosphatidylethanolamine/phosphatidyl-N-methylethanolamine N-methyltransferase	142.41	100.96
MRET_3195	N-acetylglucosaminylphosphatidylinositol deacetylase	116.07	94.41
MRET_3196	serine/threonine-protein kinase	262.99	238.08
MRET_3197	uncharacterized protein	62.21	73.64
MRET_3198	cytochrome c oxidase subunit	496.95	230.4
MRET_3199	Fes/CIP4, and EFC/F-BAR homology domain protein	209.74	98.39
MRET_3200	p38 MAP kinase	223.81	50.52
MRET_3201	calcium channel MID1	111.29	65.23
MRET_3202	uncharacterized protein	112.79	64.35
MRET_3203	uncharacterized protein	264.53	154.55
MRET_3204	oligosaccharide translocation protein RFT1	61.22	61.13
MRET_3205	C2 domain protein	80.2	45.64
MRET_3206	RNA-binding protein Musashi	2719.81	2276.76
MRET_3207	DNA-directed RNA polymerase II subunit RPB11	151.98	203.47
MRET_3208	peroxin-14	39.32	51.84
MRET_3209	vacuolar protein sorting-associated protein 45	18.72	15.63
MRET_3210	amino acid transporter	181	91.32
MRET_3211	solute carrier family 25 (mitochondrial 2-oxodicarboxylate transporter), member 21	573.47	369.39
MRET_3212	serine/threonine-protein kinase	56.52	26.09
MRET_3213	cytoplasmic tRNA 2-thiolation protein 2	82.47	43.2
MRET_3214	PAB-dependent poly(A)-specific ribonuclease subunit 3	104.4	43.88
MRET_3215	terminal uridylyltransferase	31.37	20.96
MRET_3216	uncharacterized protein	532.6	334.67

MRET_3217	oxalate---CoA ligase	45.61	13.66
MRET_3218	dual specificity phosphatase	379.5	185.96
MRET_3219	CUE domain protein	138.52	137.04
MRET_3220	transcription factor	140.27	139.06
MRET_3221	ubiquitin carboxyl-terminal hydrolase 14	102.34	139.53
MRET_3222	nucleolar complex protein 2	129.05	118.6
MRET_3223	WD repeat protein	212.46	147.71
MRET_3224	drebrin-like protein	418.19	376.54
MRET_3225	conserved hypothetical protein	56.23	63.12
MRET_3226	uncharacterized protein	6.13	4.69
MRET_3227	uncharacterized protein	21.18	27.65
MRET_3228	chromatin modification-related protein YNG2	531.78	272.96
MRET_3229	conserved hypothetical protein	158.14	122.19
MRET_3230	FAD binding domain protein	25.3	42.73
MRET_3231	purine nucleoside permease	5793.57	8519.2
MRET_3232	ADP-ribose pyrophosphatase	38.24	101.1
MRET_3233	sterol 14-demethylase	117.97	335.74
MRET_3234	homoserine dehydrogenase	113.29	118.33
MRET_3235	uncharacterized protein	270.8	130.93
MRET_3236	20S proteasome subunit beta 5	172.1	154.34
MRET_3237	polyphosphoinositide phosphatase	81.84	44.04
MRET_3238	delta24(24(1))-sterol reductase	62.92	103.13
MRET_3239	large subunit ribosomal protein L7/L12	69.65	208.52
MRET_3240	uncharacterized protein	15.4	24.21
MRET_3241	Cu+-exporting ATPase	93.22	50.75
MRET_3242	uncharacterized protein	123.89	62.31
MRET_3243	uncharacterized protein	83.8	118.7
MRET_3244	MSF1 domain protein	7.13	10.51
MRET_3245	signal recognition particle subunit SRP54	153.2	118.23
MRET_3246	phosphatidylinositol glycan, class V	69.75	56.5
MRET_3247	protein SYS1	31.8	40.81
MRET_3248	AP-2 complex subunit sigma-1	37.59	43.91
MRET_3249	required for respiratory growth protein 9, mitochondrial	550.05	316.44
MRET_3250	NADH-ubiquinone oxidoreductase 21 kDa subunit	182.63	290.83
MRET_3251	translocation protein SEC72	20.2	52.5
MRET_3252	ATP synthase mitochondrial F1 complex assembly factor 2	73.16	72.53
MRET_3253	integral peroxisomal membrane peroxin	163.98	94.78

MRET_3254	phosphatidylinositol glycan, class S	36.92	42.17
MRET_3255	uncharacterized protein	2.55	8.61
MRET_3256	uncharacterized protein	97.23	162.53
MRET_3257	stress responsive A/B barrel domain protein	655.6	805.93
MRET_3258	gem associated protein 2	26.56	50.02
MRET_3259	ribosomal RNA-processing protein 1	292.02	231.13
MRET_3260	solute carrier family 25 (mitochondrial S-adenosylmethionine transporter), member 26	180.03	89.98
MRET_3261	aspartyl-tRNA(Asn)/glutamyl-tRNA(Gln) amidotransferase subunit A	47.26	34.48
MRET_3262	SDE2 telomere maintenance homolog	367.33	119.71
MRET_3263	uncharacterized protein	116.09	37.77
MRET_3264	tetratricopeptide repeat domain protein	849.94	944.06
MRET_3265	TFIIH basal transcription factor complex TTD-A subunit	122.71	93.87
MRET_3266	thiamine pyrophosphokinase	77.23	62.82
MRET_3267	Cys-Gly metallodipeptidase DUG1	82.15	114.46
MRET_3268	proline-rich, actin-associated protein Vrp1	28.35	24.57
MRET_3269	dolichyldiphosphatase	65.68	69.39
MRET_3270	membrane associated protein-like protein	76.32	49.4
MRET_3271	solute carrier family 25 (mitochondrial carrier protein), member 16	77.71	111.28
MRET_3272	DNA helicase II/ATP-dependent DNA helicase PcrA	12.74	14.42
MRET_3273	glycerol-3-phosphate O-acyltransferase/dihydroxyacetone phosphate acyltransferase	85.9	80.07
MRET_3274	acylphosphatase	874.3	558.17
MRET_3275	DASH complex subunit DAM1	133.97	74.75
MRET_3276	uncharacterized protein	268.07	215.35
MRET_3277	uncharacterized protein	54.89	43.3
MRET_3278	short-chain dehydrogenase	61.28	50.49
MRET_3279	uncharacterized protein	33.7	32.31
MRET_3280	protoheme IX farnesyltransferase, mitochondrial	196.39	117.78
MRET_3281	CDK-activating kinase assembly factor MAT1	21.82	28.06
MRET_3282	uncharacterized protein	328.44	164.25
MRET_3283	uncharacterized protein	250.85	251.87
MRET_3284	coronin-1B/1C/6	710.35	413.67
MRET_3285	uncharacterized protein	37.45	37.86
MRET_3286	diphthine methyl ester synthase	41.81	55.72
MRET_3287	G patch domain protein 1	100.24	42.94
MRET_3288	uncharacterized protein	199.83	82.88
MRET_3289	UPF0160 domain protein MYG1	100.14	106.72
MRET_3290	zinc finger protein	278.93	289.34

MRET_3291	urease accessory protein	7.03	9.64
MRET_3292	MFS transporter	24.66	38.87
MRET_3293	vacuole protein	97.57	71.66
MRET_3294	uncharacterized protein	23.78	36.99
MRET_3295	mitotic spindle assembly checkpoint protein MAD2B	36.06	72.38
MRET_3296	uncharacterized protein	33.11	96.28
MRET_3297	ubiquitin thioesterase protein OTUB1	52.29	143.26
MRET_3298	DNA-directed RNA polymerases I and III subunit RPAC1	33.7	68.76
MRET_3299	minichromosome maintenance protein 10	8.14	11.26
MRET_3300	protein phosphatase type 1 complex subunit Hex2 Reg1	56.5	81.01
MRET_3301	conserved hypothetical protein	92.72	26.24
MRET_3302	ATP-dependent RNA helicase DDX56/DBP9	116.97	59.51
MRET_3303	transmembrane component	16.54	6.05
MRET_3304	exopolyphosphatase	60.17	34.43
MRET_3305	peroxin-5	358.67	194.69
MRET_3306	peptidyl-prolyl cis-trans isomerase-like 2	97.29	52.39
MRET_3307	uncharacterized protein	43.67	43.38
MRET_3308	beta receptor associated protein 1	87.01	39.64
MRET_3309	tRNA nucleotidyltransferase	74.2	40.24
MRET_3310	mitogen-activated protein kinase 1/3	73.87	66.27
MRET_3311	solute carrier family 35 (UDP-galactose transporter), member B1	149.66	137.95
MRET_3312	small subunit ribosomal protein S29	376.86	236.98
MRET_3313	maintenance of ploidy protein MOB2	28.8	31.52
MRET_3314	uncharacterized protein	52.09	71.19
MRET_3315	peptidyl-prolyl cis-trans isomerase-like 4	19.31	26.08
MRET_3316	elongation factor G	124.51	94.93
MRET_3317	DUF866 domain protein	127.47	69.77
MRET_3318	CDP-diacylglycerol---serine O-phosphatidyltransferase	672.46	311.59
MRET_3319	VHS domain protein	109.53	126.12
MRET_3320	serine/threonine-protein phosphatase 4 catalytic subunit	117.81	81.26
MRET_3321	(R)-2-hydroxyglutarate---pyruvate transhydrogenase	78.99	99.49
MRET_3322	mitochondrial hypoxia responsive domain protein	3876.77	3141.9
MRET_3323	phosphodiesterase	388.64	302.04
MRET_3324	nucleolar protein 53	40.17	77.64
MRET_3325	oligosaccharyltransferase complex subunit gamma	94.22	89.62
MRET_3326	uncharacterized protein	512.7	270.23
MRET_3327	mitochondrial 54S ribosomal protein YmL47	212.34	274.34

MRET_3328	golgi vesicular membrane trafficking protein	23.95	18.2
MRET_3329	orotate phosphoribosyltransferase	11.6	34.03
MRET_3330	NACHT domain protein	82.45	44.09
MRET_3331	N-alpha-acetyltransferase 38, NatC auxiliary subunit	353.88	215.4
MRET_3332	HIT family protein 1	385.85	307.79
MRET_3333	caffeine-induced death protein 2	204.66	182.54
MRET_3334	glycerol-3-phosphate dehydrogenase (NAD+)	181.53	189.71
MRET_3335	uncharacterized protein	208.36	180.56
MRET_3336	etoposide-induced 2.4 mRNA	18.62	31.4
MRET_3337	uncharacterized protein	0.34	345.83
MRET_3338	nudix family hydrolase	607.85	449.15
MRET_3339	U1 small nuclear ribonucleoprotein C	40.48	47.56
MRET_3340	ATP-dependent RNA helicase DDX49/DBP8	35.68	45.33
MRET_3341	cell wall biogenesis protein	87.42	68.83
MRET_3342	acetylornithine aminotransferase	236.94	164.27
MRET_3343	MOB kinase activator 1	342.38	1279.53
MRET_3344	acid phosphatase	212.61	108.54
MRET_3345	amidophosphoribosyltransferase	46.45	56.95
MRET_3346	solute carrier family 25, member 33/36	276.87	195.54
MRET_3347	tyrosine-protein phosphatase OCA1	129.94	159.77
MRET_3348	aldo-keto reductase	148.92	168.93
MRET_3349	chitin synthase	114.1	84.37
MRET_3350	DASH complex subunit ASK1	307.58	228.65
MRET_3351	OTU domain protein 6	155.54	156.34
MRET_3352	chromatin modification-related protein	202.73	153.93
MRET_3353	uncharacterized protein	29.65	64.44
MRET_3354	dynactin 6	14.67	28.54
MRET_3355	uncharacterized protein	2.14	6.78
MRET_3356	phospholipase C	122.21	125.22
MRET_3357	multidrug transporter	38.86	81.36
MRET_3358	uncharacterized protein	61.13	69
MRET_3359	uncharacterized protein	362.24	154.27
MRET_3360	uncharacterized protein	16.84	15.59
MRET_3361	uncharacterized protein	108.59	216.82
MRET_3362	uncharacterized protein	26.13	53.97
MRET_3363	sporulation protein RMD1	79.59	134.04
MRET_3364	GTP-binding protein	27	61.7

MRET_3365	alpha-1,3-mannosyltransferase	57.71	101.41
MRET_3366	DUF218 domain protein	28.74	69.55
MRET_3367	transmembrane protein 33	226.79	328.95
MRET_3368	RAD51-like protein	15.16	33.49
MRET_3369	DNA-directed RNA polymerase II subunit RPB1	1034.89	979.64
MRET_3370	ubiquitin binding protein	101.48	179.29
MRET_3371	conserved hypothetical protein	19.79	50.51
MRET_3372	uncharacterized protein	17.79	43.16
MRET_3373	uncharacterized protein	157.61	150.78
MRET_3374	protein of unknown function (DUF2424)	71.15	188.01
MRET_3375	iron-sulfur cluster assembly protein ISA2	2513.78	1477.24
MRET_3376	nascent polypeptide-associated complex subunit alpha	82.53	185.83
MRET_3377	20S proteasome subunit alpha 4	268.5	524.37
MRET_3378	SNARE associated golgi protein	27.55	129.72
MRET_3379	pumilio-family RNA binding repeat protein	81.41	104.07
MRET_3380	exosome complex component RRP43	35.99	60.13
MRET_3381	phosphomevalonate kinase	15.82	21.34
MRET_3382	ATP-binding cassette, subfamily F, member 3	243.56	165.98
MRET_3383	ATP-binding cassette, subfamily F, member 3	96.81	53.77
MRET_3384	uncharacterized protein	0	0
MRET_3385	lactate 2-monooxygenase	200.37	177.16
MRET_3386	ATP-binding cassette, subfamily F, member 3	91.72	49.9
MRET_3387	uncharacterized protein	0	0
MRET_3388	lactate 2-monooxygenase	199.41	177.8
MRET_3389	transcription initiation factor TFIIH subunit 1	42.84	54.13
MRET_3390	cell division cycle protein 37	37.08	40.94
MRET_3391	protein LSM14	1148.69	1391.15
MRET_3392	fungal Zn(2)-Cys(6) binuclear cluster domain protein	98.18	128.59
MRET_3393	vacuolar protein sorting-associated protein 29	93.99	199.22
MRET_3394	uncharacterized protein	81.67	120.45
MRET_3395	lipoyl(octanoyl) transferase	15.63	32.31
MRET_3396	protein of unknown function (DUF3712)	213.37	410.13
MRET_3397	actin-related protein 5	117.36	205.25
MRET_3398	myo-inositol-1-phosphate synthase	645.95	439.24
MRET_3399	PLP dependent protein	50.19	95.25
MRET_3400	ribosome maturation protein SDO1	1209.59	933.46
MRET_3401	sulfate permease, SulP family	124.6	121.61

MRET_3402	uncharacterized protein	29.12	73.29
MRET_3403	translation initiation factor 4A	1339.27	1213.3
MRET_3404	plasma membrane sulfite pump involved in sulfite metabolism	24.82	41.28
MRET_3405	dolichyl-phosphate-mannose-protein mannosyltransferase	22.73	40.46
MRET_3406	target of rapamycin complex subunit LST8	26.76	61.55
MRET_3407	large subunit ribosomal protein L44	49.62	149.24
MRET_3408	signal recognition particle receptor subunit alpha	44.54	109.97
MRET_3409	uncharacterized protein	26.37	73.68
MRET_3410	solute carrier family 9 (sodium/hydrogen exchanger), member 6/7	42.83	101.49
MRET_3411	proline iminopeptidase	65.83	127.4
MRET_3412	SWI/SNF related-matrix-associated actin-dependent regulator of chromatin subfamily C	75.55	104.63
MRET_3413	uncharacterized protein	526.47	499.82
MRET_3414	Rho GTPase activating protein	159.4	83.55
MRET_3415	glycoside hydrolase family 5 protein	11.58	224.57
MRET_3416	uncharacterized protein	11.36	32.46
MRET_3417	cellulase (glycosyl hydrolase family 5)	68.97	124.94
MRET_3418	uncharacterized protein	5.83	18.75
MRET_3419	pre-mRNA-splicing factor SYF1	14.57	37.09
MRET_3420	ubiquitin carboxyl-terminal hydrolase 10	9.94	26.93
MRET_3421	R3H domain protein	46.95	95.49
MRET_3422	WD repeat protein involved in ribosome biogenesis	19.54	69.17
MRET_3423	transcription factor SPN1	54.05	112.88
MRET_3424	small subunit ribosomal protein S9e	145.13	398.23
MRET_3425	large subunit ribosomal protein L21e	500.38	797.6
MRET_3426	carbohydrate esterase family 9 protein	194.95	125.35
MRET_3427	ankyrin repeat domain protein	100.04	169.96
MRET_3428	Sds3-like protein	43.32	61.87
MRET_3429	pyruvate dehydrogenase kinase 2/3/4	63.52	133.16
MRET_3430	F-type H+-transporting ATPase subunit b	956.73	1311.37
MRET_3431	cyclin-dependent kinase 7	77.95	78.97
MRET_3432	Rab5 GDP/GTP exchange factor	22.14	41.87
MRET_3433	nucleolar protein 12	68.98	81.52
MRET_3434	hydroxymethylglutaryl-CoA synthase	40.57	147.25
MRET_3435	uncharacterized protein	21.66	26.75
MRET_3436	AAA family ATPase	185.05	124.44
MRET_3437	ubiquinone biosynthesis monooxygenase Coq6	69.84	24.15
MRET_3438	DNA repair/transcription protein MET18/MMS19	77.16	31.82

MRET_3439	essential RNA-binding component of cleavage and polyadenylation factor	114.58	98.55
MRET_3440	DUF602 domain protein	620.12	531.24
MRET_3441	DNA-directed RNA polymerase II subunit RPB7	656.36	670.25
MRET_3442	serine/threonine-protein kinase TTK/MPS1	54.82	83.31
MRET_3443	trehalose 6-phosphate synthase complex regulatory subunit	217.94	62.06
MRET_3444	actin related protein 2/3 complex, subunit 5	74.08	92.87
MRET_3445	serine/threonine-protein kinase CLA4	206.45	157.1
MRET_3446	splicing factor U2AF 65 kDa subunit	319.78	162.61
MRET_3447	pre-mRNA-processing factor 40	420.49	334.03
MRET_3448	heat shock protein	55.58	97.16
MRET_3449	guanylate kinase	26.5	44.59
MRET_3450	uncharacterized protein	97.11	84.53
MRET_3451	cyclin-dependent kinase	296.89	373.31
MRET_3452	peroxisomal membrane protein 4	375.11	438.75
MRET_3453	uncharacterized protein	116.69	155.42
MRET_3454	pseudouridine 5'-phosphatase	381.62	528.04
MRET_3455	charged multivesicular body protein 5	211.13	354.13
MRET_3456	bridging integrator 3	155.46	162.27
MRET_3457	serine/threonine-protein phosphatase 5	21.3	48.91
MRET_3458	ribosomal prokaryotic L21 protein	97.48	107.33
MRET_3459	F-type H+-transporting ATPase subunit alpha	1004.66	1063.28
MRET_3460	dynamin 1-like protein	505.68	421.12
MRET_3461	U3 small nucleolar ribonucleoprotein protein LCP5	15.61	44.23
MRET_3462	sterol O-acyltransferase	251.92	148.95
MRET_3463	solute carrier family 35 (UDP-xylose/UDP-N-acetylglucosamine transporter), member B4	44.99	43.36
MRET_3464	ubiquitin carboxyl-terminal hydrolase 25	8.65	18.78
MRET_3465	guanine nucleotide exchange factor LTE1	8.81	17.11
MRET_3466	ribonuclease P/MRP protein subunit POP3	10.26	24.03
MRET_3467	transcription factor	33.51	38.19
MRET_3468	sortilin	38.33	37.83
MRET_3469	uncharacterized protein	17.83	28.54
MRET_3470	uncharacterized protein	9.49	22.09
MRET_3471	tRNA-dihydrouridine synthase 4	13.5	39.86
MRET_3472	histone chaperone ASF1	47.21	102.92
MRET_3473	sterol 24-C-methyltransferase	229.68	647.67
MRET_3474	U6 snRNA-associated Sm-like protein LSm8	50.45	97.36
MRET_3475	dihydroorotate dehydrogenase	68.1	80.79

MRET_3476	zinc finger protein	150.54	116
MRET_3477	structural maintenance of chromosomes protein	129.98	75.55
MRET_3478	Prm1-pheromone-regulated multispanning membrane protein	106.39	111.17
MRET_3479	RNA-binding protein with serine-rich domain 1-like protein	31.04	60.24
MRET_3480	protein of unknown function (DUF2418)	44.47	63.75
MRET_3481	uncharacterized protein	86.49	64.37
MRET_3482	cyclin-dependent kinase regulatory subunit CKS1	420.21	402.79
MRET_3483	aldo/keto reductase family	2666.3	2843.5
MRET_3484	aldo/keto reductase family	657.43	668.51
MRET_3485	aldo/keto reductase family	67.11	102.29
MRET_3486	mitochondrial protein FMP25	33.61	62.03
MRET_3487	glycyl-tRNA synthetase	56.87	82.05
MRET_3488	uncharacterized protein	52.87	51.91
MRET_3489	H/ACA ribonucleoprotein complex subunit 3	59.3	142.02
MRET_3490	p24 family protein delta-1	293.98	263.32
MRET_3491	E3 ubiquitin-protein ligase UBR7	162.04	173.26
MRET_3492	uncharacterized protein	417.52	237.32
MRET_3493	uncharacterized protein	23.38	36.43
MRET_3494	fanconi-associated nuclease 1	61.05	58.58
MRET_3495	myosin heavy chain	68.94	142.68
MRET_3496	histone acetyltransferase	20.09	51.63
MRET_3497	CBF NF-Y family transcription factor	43.41	97.9
MRET_3498	platelet-activating factor acetylhydrolase IB subunit alpha	19.9	58.18
MRET_3499	kinesin family member 4/21/27	36.1	43.05
MRET_3500	3-deoxy-7-phosphoheptulonate synthase	54.57	61.46
MRET_3501	negative cofactor 2	43.98	123.99
MRET_3502	methylenetetrahydrofolate dehydrogenase (NADP+)	71.55	191.01
MRET_3503	RNA-binding protein	60.6	87.92
MRET_3504	uncharacterized protein	12.51	21.32
MRET_3505	F-type H+-transporting ATPase subunit beta	1016.06	842.65
MRET_3506	septin 3/9/12	220.38	385.74
MRET_3507	secretory carrier-associated membrane protein	73.55	96.86
MRET_3508	component of the RSC chromatin remodeling complex	65.48	59.66
MRET_3509	secretory pathway protein Sec39	127.13	68.96
MRET_3510	betaine lipid synthase	74.12	92.01
MRET_3511	AP-3 complex subunit delta	21.4	43.17
MRET_3512	glutamine amidotransferase	303.23	305.21

MRET_3513	uncharacterized protein	93.71	70.33
MRET_3514	exosome complex component RRP46	132.03	126.89
MRET_3515	mitochondrial import inner membrane translocase subunit TIM54	31.14	55.88
MRET_3516	tRNA (uracil-5-)-methyltransferase TRM9	52.36	83.09
MRET_3517	5'-methylthioadenosine phosphorylase	114.3	172.94
MRET_3518	mRNA capping protein	14.71	48.37
MRET_3519	catalytic protein kinase domain protein	104.06	141.27
MRET_3520	DNA mismatch repair protein MSH6	96.22	57.06
MRET_3521	serine/threonine-protein kinase mTOR	54.95	47.08
MRET_3522	phospholipase A2 activating protein	74.28	116.45
MRET_3523	PQ loop repeat protein	74.97	107.75
MRET_3524	endonuclease G, mitochondrial	81.53	149.15
MRET_3525	ornithine--oxo-acid transaminase	65.42	103.03
MRET_3526	heme steroid binding protein	115.28	117.74
MRET_3527	H/ACA ribonucleoprotein complex subunit 2	62.73	176.35
MRET_3528	tryptophanyl-tRNA synthetase	16.63	26.84
MRET_3529	peroxin-13	257.25	284.37
MRET_3530	large subunit ribosomal protein L10e	6188.26	5243.83
MRET_3531	uncharacterized protein	49.12	97.58
MRET_3532	replication factor A1	557.09	339.98
MRET_3533	phospholipid binding protein	91.88	119.41
MRET_3534	vacuolar protein sorting-associated protein IST1	6.78	11.72
MRET_3535	DNA primase small subunit	224.34	150.71
MRET_3536	CAP-Gly domain protein	351.72	167.66
MRET_3537	glutamate decarboxylase	192.78	140.33
MRET_3538	serine/threonine-protein kinase	77.75	39.86
MRET_3539	breast cancer 2 susceptibility protein	250.63	232.04
MRET_3540	kinase phosphorylation protein	635.53	353.41
MRET_3541	glucosamine---fructose-6-phosphate aminotransferase	170.88	286.62
MRET_3542	chromosome transmission fidelity protein 1	16.78	31.94
MRET_3543	phosphoglycerate mutase family protein	71.23	106.75
MRET_3544	golgi matrix protein	23.64	47.47
MRET_3545	small subunit ribosomal protein S20e	82.22	442.92
MRET_3546	large subunit ribosomal protein L27Ae	433.23	1111.11
MRET_3547	ariadne-1	203.42	148.31
MRET_3548	3-hydroxyacyl-CoA dehydrogenase	378.68	516.93
MRET_3549	dolichol-phosphate mannosyltransferase	106.16	142.42

MRET_3550	WD repeat and SOF domain protein 1	106.93	100.61
MRET_3551	conserved hypothetical protein	42.95	28.36
MRET_3552	ribose-phosphate pyrophosphokinase	98.3	143.71
MRET_3553	small nuclear ribonucleoprotein D1	264.59	293.57
MRET_3554	exonuclease 1	44.54	88.36
MRET_3555	HCNGP-like protein	47.94	96.49
MRET_3556	uncharacterized protein	61.92	101.07
MRET_3557	UDP-glucose 6-dehydrogenase	697.58	596.83
MRET_3558	conserved hypothetical protein	223.83	163.13
MRET_3559	ribosome biogenesis protein UTP30	27.52	76.78
MRET_3560	ER membrane DUF1077 domain protein	16.58	47.47
MRET_3561	cytochrome c oxidase subunit 20	34.27	74.44
MRET_3562	pyridine nucleotide-disulphide oxidoreductase	265.73	230.63
MRET_3563	small subunit ribosomal protein S24e	348.81	837.01
MRET_3564	U6 snRNA-associated Sm-like protein LSm3	62.6	112.13
MRET_3565	large subunit ribosomal protein L5	44.24	61.78
MRET_3566	DNA damage-responsive protein	14.42	22.64
MRET_3567	vacuolar protein sorting-associated protein 41	81.82	50.64
MRET_3568	NADH dehydrogenase (ubiquinone) 1 alpha subcomplex subunit 13	54.65	80.08
MRET_3569	nucleolar protein 14	55.47	95.24
MRET_3570	DCN1-like protein 1/2	267.22	262.02
MRET_3571	small nuclear ribonucleoprotein F	204.76	298.77
MRET_3572	solute carrier family 45, member 1/2/4	69.45	79.75
MRET_3573	14-3-3 protein epsilon	2900.7	2104.63
MRET_3574	AP-2 complex subunit alpha	9.4	16.09
MRET_3575	RNA polymerase I-specific transcription initiation factor RRN3	382.49	236.35
MRET_3576	GTP-binding nuclear protein Ran	339.28	459.93
MRET_3577	DNA-directed RNA polymerase I subunit RPA43	32.62	81.88
MRET_3578	thioredoxin	65.71	104.54
MRET_3579	negative regulation of cAMP metabolic process	6.23	13.98
MRET_3580	putative methyltransferase	45.74	30.35
MRET_3581	uncharacterized protein	84.37	88.97
MRET_3582	mRNA m6A methyltransferase	39.59	98.64
MRET_3583	uncharacterized protein	29.59	32.89
MRET_3584	uncharacterized protein	26.63	55.89
MRET_3585	F-box and WD-40 domain protein 1/11	86.28	247.06
MRET_3586	Pal1 cell morphology protein	232.92	260.84

MRET_3587	lipid transfer protein	1354.37	1324.07
MRET_3588	collagen	100.8	206.58
MRET_3589	putative sensor/transporter protein involved in cell wall biogenesis	80.24	98.82
MRET_3590	eukaryotic mitochondrial regulator protein	2183.62	829.5
MRET_3591	uncharacterized protein	127.83	111.54
MRET_3592	cortical actin cytoskeleton protein asp1	98.39	57.11
MRET_3593	LETM1-like protein	117.02	247.36
MRET_3594	U6 snRNA-associated Sm-like protein LSm1	86.11	223.22
MRET_3595	AdoMet-dependent methyltransferase	64.94	138.74
MRET_3596	WD40 repeat-like protein	10.18	20.23
MRET_3597	trafficking protein particle complex subunit 8	19.91	32.04
MRET_3598	integral peroxisomal membrane peroxin	20.66	11.12
MRET_3599	nitric oxide dioxygenase	380.1	54.74
MRET_3600	nucleolin	30.8	72.79
MRET_3601	DNA-directed RNA polymerase III subunit RPC1	112.06	74.33
MRET_3602	protein unc-45	27.67	27.3
MRET_3603	ubiquitin-activating enzyme E1	777.99	481.84
MRET_3604	uncharacterized protein	187.29	145.71
MRET_3605	SRP40, C-terminal domain protein	277.84	1225.93
MRET_3606	mediator of RNA polymerase II transcription subunit 13	94.7	50.21
MRET_3607	uncharacterized protein	50.74	46.43
MRET_3608	kinesin-like protein 8	20.81	18.47
MRET_3609	uncharacterized protein	28.82	17.82
MRET_3610	homoisocitrate dehydrogenase	13.62	37.26
MRET_3611	elongation factor	735.06	507
MRET_3612	elongation factor 1 alpha-like protein	782.99	641.97
MRET_3613	Myb-like DNA-binding domain protein	151.36	52.89
MRET_3614	trafficking protein particle complex subunit 2	59.18	101.26
MRET_3615	uncharacterized protein	252.15	391.99
MRET_3616	protein kinase C substrate 80K-H	27.02	51.12
MRET_3617	lysophosphatidate acyltransferase	77.1	99.2
MRET_3618	uncharacterized protein	56.56	63.38
MRET_3619	TBC domain protein	635.64	721.5
MRET_3620	Rab5-interacting protein (Rab5ip)	363.5	459.85
MRET_3621	COMPASS component SWD1	141.9	136.55
MRET_3622	ATP-dependent Clp protease ATP-binding subunit ClpB	1660.48	1980.09
MRET_3623	transcription factor	45.03	43.39

MRET_3624	RNA recognition motif domain protein	283.87	274.05
MRET_3625	aldehyde dehydrogenase (NAD+)	319.44	217.66
MRET_3626	serine/threonine-protein kinase	181.96	142.43
MRET_3627	phosphoserine phosphatase	49.92	52.59
MRET_3628	20S proteasome subunit alpha 2	104.09	198.85
MRET_3629	ribosome biogenesis protein ENP2	34.88	47.36
MRET_3630	large subunit ribosomal protein L11	13.25	93.23
MRET_3631	sorbose reductase	794.79	709.34
MRET_3632	iron-sulfur cluster assembly enzyme ISCU, mitochondrial	1888.51	1866.17
MRET_3633	charged multivesicular body protein 6	77.69	150.91
MRET_3634	ribosomal RNA-processing protein 36	38.86	52.61
MRET_3635	U3 small nucleolar RNA-associated protein 14	28.17	54.56
MRET_3636	60S ribosome subunit biogenesis protein NIP7	37.84	59
MRET_3637	AHNAK nucleoprotein	181.16	140.21
MRET_3638	ribosomal RNA-processing protein 8	64.61	59.21
MRET_3639	brefeldin A-inhibited guanine nucleotide-exchange protein	45.59	43.34
MRET_3640	translation initiation factor 6	323.04	251.76
MRET_3641	mitochondrial import inner membrane translocase subunit TIM9	49.23	227.11
MRET_3642	uncharacterized protein	16.47	62.4
MRET_3643	THO complex subunit 2	43.39	67.79
MRET_3644	DNA-directed RNA polymerases I, II, and III subunit RPABC4	279.76	262.89
MRET_3645	AP-3 complex subunit mu	39.44	58.75
MRET_3646	ubiquitin metalloprotease fusion protein	201.68	204.07
MRET_3647	glycosyl hydrolase catalytic core	116.73	508.43
MRET_3648	bud emergence protein 1	13.75	41.05
MRET_3649	uncharacterized protein	50.41	105.81
MRET_3650	proton-dependent oligopeptide transporter, POT family	46.5	70.8
MRET_3651	uncharacterized protein	198.1	225.89
MRET_3652	guanyl nucleotide binding protein	55	106.45
MRET_3653	mitochondrial 37S ribosomal protein NAM9	19.03	65.94
MRET_3654	S-adenosylmethionine synthetase	203.53	260.59
MRET_3655	uncharacterized protein	24.57	36.64
MRET_3656	sulfite reductase (NADPH) flavoprotein alpha-component	71.56	78.05
MRET_3657	small subunit ribosomal protein S7e	37.05	230.71
MRET_3658	DUF1768 domain protein	24.45	111.84
MRET_3659	mitochondrial alcohol dehydrogenase isozyme III	26.56	64.86
MRET_3660	oligosaccharyltransferase complex subunit epsilon	63.42	86.3

MRET_3661	peptide chain release factor subunit 3	454.25	387.88
MRET_3662	conserved hypothetical protein	90.44	102.18
MRET_3663	Rho GDP-dissociation inhibitor	820.72	532.63
MRET_3664	coupling of ubiquitin conjugation to ER degradation protein 1	252.88	231.35
MRET_3665	NADH dehydrogenase (ubiquinone) 1 beta subcomplex subunit 9	679.78	768.1
MRET_3666	flavin reductase domain protein	441.21	311.35
MRET_3667	trafficking protein particle complex subunit 2	77.18	129.1
MRET_3668	uncharacterized protein	244.6	207.78
MRET_3669	fungal protein of unknown function (DUF1748)	349.2	164.12
MRET_3670	serine/threonine-protein kinase ULK2	1303.73	1080.01
MRET_3671	protein-serine/threonine kinase	18.46	33.01
MRET_3672	U4/U6.U5 tri-snRNP-associated protein 1	45.17	65.43
MRET_3673	RhoGAP	45.12	86.45
MRET_3674	uncharacterized protein	179.09	105.15
MRET_3675	CCR4-NOT transcription complex subunit 3	73.11	96.05
MRET_3676	large subunit ribosomal protein L19	30.02	25.17
MRET_3677	small subunit ribosomal protein S14	74.45	47.41
MRET_3678	5-aminolevulinate synthase	706.74	224.22
MRET_3679	integral membrane protein	134.27	143.98
MRET_3680	alpha/beta-hydrolase	98.73	62.95
MRET_3681	uncharacterized protein	38.19	47.31
MRET_3682	cytochrome c oxidase assembly factor 5	274.21	559.27
MRET_3683	conserved serine/proline-rich protein	65.22	68.68
MRET_3684	uncharacterized protein	95.43	71.5
MRET_3685	Ras GTPase-activating-like protein IQGAP2/3	15.16	30.4
MRET_3686	coatomer subunit zeta	477.87	832.31
MRET_3687	TBC1 domain family member 20	219.68	134.8
MRET_3688	Ras homolog enriched in brain	44.51	80.8
MRET_3689	D-xylulose reductase	791.41	429.95
MRET_3690	uncharacterized protein	24.8	30.58
MRET_3691	gamma-tubulin complex component 3	93.22	54.03
MRET_3692	endosome-associated ubiquitin isopeptidase (AmsH)	31.84	22.49
MRET_3693	paxillin	608.56	309.34
MRET_3694	uncharacterized protein	35.54	70.82
MRET_3695	DNA replication licensing factor MCM7	117.15	103.26
MRET_3696	nucleoporin NDC1	123.38	58.54
MRET_3697	superoxide dismutase, Fe-Mn family	54.87	63.68

MRET_3698	SNARE associated golgi protein	112.79	204.68
MRET_3699	DUF431 domain protein	32.48	29.72
MRET_3700	DUF775 domain protein	253.03	125.95
MRET_3701	ribokinase	31.11	33.49
MRET_3702	general repressor of transcription	20.69	23.31
MRET_3703	threonine synthase	97.07	92.38
MRET_3704	riboflavin kinase	71.93	54.81
MRET_3705	uncharacterized protein	91.16	54.19
MRET_3706	RNA-binding protein	150.48	186.42
MRET_3707	chromatin structure-remodeling complex subunit SFH1	52.08	81.4
MRET_3708	small subunit ribosomal protein S3Ae	89.75	597.14
MRET_3709	protein of unknown function (DUF788)	135.59	12.06
MRET_3710	transcription factor	76.43	35.41
MRET_3711	RF-1 domain protein	562.04	375.09
MRET_3712	AMMECR1 family protein	13.91	8.48
MRET_3713	phospholipid-binding protein that interacts with both Ypt7p and Vps33p	46.85	75.76
MRET_3714	myosin regulatory light chain cdc4	660.44	867.74
MRET_3715	small subunit ribosomal protein S25e	257.44	880.06
MRET_3716	40S ribosomal protein S25	47.67	122.99
MRET_3717	F-type H+-transporting ATPase subunit d	513.03	696.03
MRET_3718	cAMP-dependent protein kinase regulator	135.21	161.27
MRET_3719	aspartyl-tRNA synthetase	64.57	51.07
MRET_3720	Tol-Pal system protein YbgF	900.92	1263.11
MRET_3721	conserved hypothetical protein	57.14	138.34
MRET_3722	uncharacterized protein	535.17	421.74
MRET_3723	Shwachman-Bodian-Diamond syndrome (SBDS) protein	2936.2	2704.36
MRET_3724	pleiotropic regulator 1	458.68	445.47
MRET_3725	N-acetylated-alpha-linked acidic dipeptidase	255.06	164.59
MRET_3726	20S proteasome subunit alpha 6	424.76	381.12
MRET_3727	signal recognition particle receptor beta subunit	9.24	25.71
MRET_3728	ribosome biogenesis protein ERB1	61.98	75.43
MRET_3729	oxidoreductase	104.06	59.75
MRET_3730	transporter	91.06	94.54
MRET_3731	charged multivesicular body protein 4	67.84	181.44
MRET_3732	transporter	167.28	234.12
MRET_3733	phosphatidylinositol 4-kinase B	19.82	25.64
MRET_3734	transformation/transcription domain-associated protein	291.64	231.65

MRET_3735	DNA-binding protein	17.02	19.98
MRET_3736	transporter	36.21	2.92
MRET_3737	Chs5-Arf1p-binding protein BUD7/BCH1	16.38	34.68
MRET_3738	solute carrier family 25 (mitochondrial carnitine/acylcarnitine transporter), member 20/29	22.93	88.54
MRET_3739	DnaJ homolog subfamily B member 12	63.95	424.58
MRET_3740	DNA-directed RNA polymerase I subunit RPA12	15.05	23.65
MRET_3741	hydroxyacylglutathione hydrolase	179.66	0.41
MRET_3742	short-chain dehydrogenase	541.52	985.68
MRET_3743	protein HIRA/HIR1	11.4	13.8
MRET_3744	uncharacterized protein	21.02	27.59
MRET_3745	histone acetyltransferase	110.59	85.1
MRET_3746	mitochondrial distribution and morphology protein 34	263.66	173.01
MRET_3747	TBC domain protein	16.91	38.55
MRET_3748	TBC domain protein	20.64	44.06
MRET_3749	cyclin	832.34	728.39
MRET_3750	uncharacterized protein	50.18	41.86
MRET_3751	magnesium transporter	80.54	87.85
MRET_3752	transaldolase	622.57	655.62
MRET_3753	mediator of RNA polymerase II transcription subunit 11	608.22	553.08
MRET_3754	N-acetyltransferase 10	23.35	20.46
MRET_3755	type I protein arginine methyltransferase	11.53	20.7
MRET_3756	Bromodomain associated protein	35.08	50.22
MRET_3757	NADH dehydrogenase (ubiquinone) Fe-S protein 2	266.18	308.05
MRET_3758	endoplasmic reticulum-golgi intermediate compartment protein 3	120.21	111.71
MRET_3759	response regulator receiver domain protein	32.85	44.29
MRET_3760	tubulin-specific chaperone B	67.39	37.36
MRET_3761	GINS complex subunit 1	36.75	35.29
MRET_3762	uncharacterized protein	318.34	180.24
MRET_3763	sister chromatid cohesion protein DCC1	15.03	39.11
MRET_3764	solute carrier family 24 (sodium/potassium/calcium exchanger), member 6	21.42	29.66
MRET_3765	triacylglycerol lipase	44.78	15.81
MRET_3766	serine/threonine-protein phosphatase	119.36	42.21
MRET_3767	uncharacterized protein	122.84	264.28
MRET_3768	uncharacterized protein	62.7	102.48
MRET_3769	uncharacterized protein	257.39	94.23
MRET_3770	eukaryotic aspartyl protease	9.66	82.85
MRET_3771	deoxyribodipyrimidine photo-lyase	56.57	75.84

MRET_3772	lipase precursor-like protein	28.34	60.43
MRET_3773	MFS family protein	93.43	170.23
MRET_3774	multidrug transporter of the major facilitator superfamily	205.69	243.56
MRET_3775	multidrug transporter of the major facilitator superfamily	464.06	247.73
MRET_3776	glutathione S-transferase	737.76	285.68
MRET_3777	mitochondrial intermediate peptidase	154.81	107.85
MRET_3778	phenylalanyl-tRNA synthetase alpha chain	74.24	87.24
MRET_3779	U4/U6 small nuclear ribonucleoprotein PRP4	127.49	118.25
MRET_3780	large subunit ribosomal protein L24e	141.13	175.83
MRET_3781	glutaredoxin domain protein	68.77	143.21
MRET_3782	signal recognition particle subunit SRP9	13.51	41.5
MRET_3783	uncharacterized protein	72.31	96.77
MRET_3784	FYVE, RhoGEF and PH domain protein	13.29	34.66
MRET_3785	Mus7/MMS22 family protein	26.04	20.97
MRET_3786	sphingomyelin phosphodiesterase	158.93	145.26
MRET_3787	proteasomal ATPase-associated factor 1	379.67	143.7
MRET_3788	transporter (MirC)	167.3	128.2
MRET_3789	RNA binding effector protein Scp160	79.41	93.7
MRET_3790	DUF907 domain protein	357.35	208.1
MRET_3791	nicotinamidase	858.1	558.17
MRET_3792	protein phosphatase PTC7	44.41	47.18
MRET_3793	component of the NuA4 histone acetyltransferase complex	304.59	91.85
MRET_3794	uncharacterized protein	174.49	111.88
MRET_3795	uncharacterized protein	42.13	61.78
MRET_3796	NADH dehydrogenase (ubiquinone) Fe-S protein 4	107.8	185.6
MRET_3797	exosome complex component RRP40	46.77	48.13
MRET_3798	vacuolar protein sorting-associated protein 53	128.82	128.08
MRET_3799	nucleosome assembly protein 1-like 1	303.91	427.19
MRET_3800	DNA polymerase kappa	45.81	50.8
MRET_3801	pH-response regulator protein palC	37.51	47.08
MRET_3802	F-type H+-transporting ATPase subunit f	466.17	602.31
MRET_3803	uncharacterized protein	92.33	387.38
MRET_3804	small subunit ribosomal protein SAe	226.46	595.76
MRET_3805	histone acetyltransferase HTATIP	288.25	257.32
MRET_3806	splicing factor 3B subunit 1	255.7	164.1
MRET_3807	U3 small nucleolar RNA-associated protein 13	32.4	46.74
MRET_3808	uncharacterized protein	318.66	508.13

MRET_3809	telomerase reverse transcriptase	154.61	123.39
MRET_3810	PRA1 family protein 1	1039.89	699.23
MRET_3811	mitochondrial carrier protein	1079.09	563
MRET_3812	uncharacterized protein	197.33	248.13
MRET_3813	mitochondrial carrier protein	62.83	55.94
MRET_3814	uncharacterized protein	55.52	69.65
MRET_3815	component of nuclear aminoacylation-dependent tRNA export pathway	55.84	68.85
MRET_3816	mediator of RNA polymerase II transcription subunit 14	8.48	16.32
MRET_3817	maltose acetyltransferase	56.02	176.89
MRET_3818	N-terminal acetyltransferase B complex non-catalytic subunit	29.07	32.68
MRET_3819	crossover junction endonuclease EME1	47.2	50.9
MRET_3820	phosphatidylinositol glycan, class B	37.88	46.42
MRET_3821	mitogen-activated protein kinase kinase	187.33	145.41
MRET_3822	autophagy-related protein 13	187.94	139.91
MRET_3823	peroxin-16	272.84	204.89
MRET_3824	methyltransferase	63.62	56.99
MRET_3825	lanosterol synthase	522.06	140.99
MRET_3826	transmembrane protein 167	35.55	34.78
MRET_3827	26S proteasome non-ATPase regulatory subunit 10	41.07	50.33
MRET_3828	transcription initiation factor TFIID subunit 11	69.05	89.71
MRET_3829	saccharopine dehydrogenase	40.26	50.39
MRET_3830	tubulin beta	183.81	211.89
MRET_3831	filamentation protein (Rhf1)	144.58	81.18
MRET_3832	carbon catabolite-derepressing protein kinase	60.81	56.95
MRET_3833	Ca2+:H+ antiporter	46.04	45.11
MRET_3834	epsin	80.65	178.7
MRET_3835	ammonium transporter, Amt family	38.69	36.78
MRET_3836	tRNA pseudouridine13 synthase	56.28	50.51
MRET_3837	Ras-related protein Rab-18	35.69	35.49
MRET_3838	UPF0172 domain protein	95.28	98.47
MRET_3839	adrenodoxin-NADP+ reductase	18.12	29.57
MRET_3840	splicing factor 3A subunit 2	22.93	45.49
MRET_3841	cell division control protein 12	405.39	428.38
MRET_3842	DNA repair and recombination protein RAD52	25.18	90.5
MRET_3843	vesicle transport protein SEC22	78.64	148.87
MRET_3844	general stress response protein Whi2	196.06	465.98
MRET_3845	uncharacterized protein	124.58	151.34

MRET_3846	small subunit ribosomal protein S10	77.95	130.6
MRET_3847	phospholipase A2	32.74	32.13
MRET_3848	uncharacterized protein	471.74	226.03
MRET_3849	transcription initiation protein SPT3	91.38	83.73
MRET_3850	FHA domain protein	163.93	141.21
MRET_3851	elongation factor G	78.1	110.93
MRET_3852	STE24 endopeptidase	53.83	43.06
MRET_3853	STE24 endopeptidase	554.19	320.16
MRET_3854	DNA ligase 1	48.12	55.42
MRET_3855	structural maintenance of chromosomes protein	55.62	47.07
MRET_3856	solute carrier family 25 (mitochondrial aspartate/glutamate transporter), member 12/13	71.38	75.53
MRET_3857	chaperonin GroEL	1715.35	2307.74
MRET_3858	chaperonin GroES	1531.6	1049.56
MRET_3859	putative mago nashi protein, exon junction complex	49.29	156.57
MRET_3860	N-glycosylation protein	53.9	66.35
MRET_3861	uncharacterized protein	174.73	147.33
MRET_3862	uncharacterized protein	13.14	11.82
MRET_3863	mitochondrial import receptor subunit TOM70	385.12	271.72
MRET_3864	small ubiquitin-related modifier	450.91	527.23
MRET_3865	ESF2/ABP1 family protein	82.84	84.7
MRET_3866	diphthamide biosynthesis protein 3	112.93	68.93
MRET_3867	DUF1708 domain protein	188.2	122.06
MRET_3868	putative subunit of the 90S preribosome processome complex	182.48	159.56
MRET_3869	Dr1-associated corepressor	218.33	255.77
MRET_3870	carbamoyl-phosphate synthase/aspartate carbamoyltransferase	309.47	100.35
MRET_3871	uncharacterized protein	25.56	22.81
MRET_3872	BAR domain protein	92.1	99.08
MRET_3873	mortality factor 4-like protein 1	38.17	60.57
MRET_3874	uncharacterized protein	99	136.18
MRET_3875	mitochondrial import inner membrane translocase subunit TIM13	25.15	44.59
MRET_3876	uncharacterized protein	20.64	27.96
MRET_3877	Bromodomain associated protein	18.73	29.83
MRET_3878	tRNA (guanine37-N1)-methyltransferase	15.8	24.39
MRET_3879	uncharacterized protein	638.03	361.09
MRET_3880	cation efflux family protein	45.18	27.71
MRET_3881	20S proteasome subunit beta 3	56.12	112.92
MRET_3882	ATPase family AAA domain protein	194.94	141.06

MRET_3883	ATP phosphoribosyltransferase	149.68	189.52
MRET_3884	transcription initiation factor TFIIIB 90 kDa subunit	68.01	66.13
MRET_3885	CobW domain protein	51.51	61.9
MRET_3886	DUF202 domain protein	15.56	16.1
MRET_3887	SH3 domain protein	28.75	82.09
MRET_3888	ribonuclease P/MRP protein subunit POP5	62.32	226.72
MRET_3889	Mob1/phocein family	178.99	451.02
MRET_3890	zinc finger protein, C3HC4 type (RING finger)	2102.8	1445.2
MRET_3891	vacuolar protein-sorting protein BRO1	367.43	142.88
MRET_3892	condensin complex subunit 3	18.7	57.18
MRET_3893	lipoate---protein ligase	327.96	239.04
MRET_3894	chitin synthase	54.89	56.44
MRET_3895	uncharacterized protein	438.62	218.55
MRET_3896	uncharacterized protein	97.86	52.61
MRET_3897	charged multivesicular body protein 1	52.74	107.07
MRET_3898	protein of unknown function (DUF2416)	9.78	10.59
MRET_3899	mitochondrial distribution and morphology protein 31	96.71	55.31
MRET_3900	centromere protein k	30.39	31.78
MRET_3901	peroxisomal carrier protein	133.6	86.09
MRET_3902	UTP--glucose-1-phosphate uridylyltransferase	91.4	103.98
MRET_3903	capping protein (actin filament) muscle Z-line, beta	35.53	63.44
MRET_3904	protein phosphatase 1 regulatory subunit 7	179.63	118.23
MRET_3905	large subunit ribosomal protein L41	14.09	57.99
MRET_3906	ATP-binding cassette, subfamily F, member 2	86.03	98.29
MRET_3907	nuclear transcription Y subunit beta	62.07	170.1
MRET_3908	protein cornichon	72.07	266.9
MRET_3909	structural maintenance of chromosomes protein	26.93	46.41
MRET_3910	uncharacterized protein	793.52	299.27
MRET_3911	large subunit ribosomal protein L13	169.43	181.38
MRET_3912	clathrin light chain	82.14	141.73
MRET_3913	DNA-binding protein	30.43	41.42
MRET_3914	NAD binding dehydrogenase family protein	79.55	93.4
MRET_3915	cofilin/tropomyosin-type actin-binding protein	80.28	70.46
MRET_3916	NAD binding dehydrogenase family protein	45.62	120.18
MRET_3917	conserved hypothetical protein	265.68	406.32
MRET_3918	RNA-binding protein NOB1	80.17	129.75
MRET_3919	DnaJ domain protein	164.54	95.01

MRET_3920	polarized growth protein	104.94	188.58
MRET_3921	metacaspase-1	570.95	359.4
MRET_3922	tubulin-specific chaperone C	20.18	18.41
MRET_3923	ubiquitin carboxyl-terminal hydrolase 36/42	41.58	39.09
MRET_3924	vacuole morphology and inheritance protein 14	46.69	44.02
MRET_3925	beta-catenin-like protein 1	58.21	49.32
MRET_3926	poly(A) RNA-binding protein	190.07	86.62
MRET_3927	uncharacterized protein	441.86	775.25
MRET_3928	nicotinamide-nucleotide adenylyltransferase	68.22	81.9
MRET_3929	pre-mRNA-splicing factor ISY1	19.45	55.48
MRET_3930	large subunit ribosomal protein L10Ae	223.92	623.84
MRET_3931	small subunit ribosomal protein S11e	114	526.47
MRET_3932	small subunit ribosomal protein S12e	312.84	1012.46
MRET_3933	histone deacetylase complex subunit SAP18	388.11	402.4
MRET_3934	uncharacterized protein	82.87	44.3
MRET_3935	uncharacterized protein	118.17	65.79
MRET_3936	tubulin-specific chaperone E	40.41	22.55
MRET_3937	uncharacterized protein	98.36	45.68
MRET_3938	ribosome assembly protein 1	170.69	119.12
MRET_3939	Prp8 binding protein	94.8	89.9
MRET_3940	vacuolar protein sorting-associated protein	70.01	18.83
MRET_3941	lysophospholipid acyltransferase	46.08	28.32
MRET_3942	palmitoyl-protein thioesterase	25.56	24.08
MRET_3943	phosphatidylglycerol phospholipase C	118.67	136.16
MRET_3944	vaculolar membrane protein	63.49	63.01
MRET_3945	uncharacterized protein	279.07	139.73
MRET_3946	peptidyl-prolyl cis-trans isomerase-like 3	227.22	173.05
MRET_3947	vacuolar transporter chaperone 1	207.47	235.79
MRET_3948	modifier of rudimentary (Mod(r)) protein	120.62	79.4
MRET_3949	Myb-like DNA-binding domain protein	56.55	70.99
MRET_3950	coatomer subunit beta'	86.71	90.13
MRET_3951	F-box and leucine-rich repeat protein GRR1	167.33	118.01
MRET_3952	uncharacterized protein	70.38	68.21
MRET_3953	uncharacterized protein	450.9	289.28
MRET_3954	transcription initiation factor TFIID subunit 5	30.34	45.86
MRET_3955	serine palmitoyltransferase	154.98	193.31
MRET_3956	ubiquitin-conjugating enzyme E2 A	73.69	174.87

MRET_3957	uncharacterized protein	245.13	451.5
MRET_3958	trimethylguanosine synthase	95.93	245.21
MRET_3959	exosome complex component MTR3	83.97	139.43
MRET_3960	glycoside hydrolase family 16 protein	5786.22	8077.37
MRET_3961	DNA repair and recombination protein RAD54 and RAD54-like protein	445.52	261.43
MRET_3962	transcription initiation factor TFIIH subunit 4	47.02	78.39
MRET_3963	acyl-CoA dehydrogenase	580.43	442.35
MRET_3964	putative phosphomutase	75.19	94.9
MRET_3965	uncharacterized protein	76.33	112.37
MRET_3966	phospholipid-translocating ATPase	55.99	78.04
MRET_3967	SUN domain protein (Adg3)	233.03	195.78
MRET_3968	uncharacterized protein	1579.5	1337.47
MRET_3969	hexosyltransferase	80.6	61.69
MRET_3970	inner centromere protein	19.69	51.89
MRET_3971	SNARE complex subunit Vam7	34.74	54.64
MRET_3972	general repressor of transcription	231.1	218.15
MRET_3973	serine/arginine repetitive matrix protein 1	436.45	266.42
MRET_3974	ribose-phosphate pyrophosphokinase	74.29	125.47
MRET_3975	ATP-dependent RNA helicase DDX55/SPB4	59.04	25.67
MRET_3976	GRAM domain protein	57.75	44.79
MRET_3977	AN1-like zinc finger protein	4.82	7.31
MRET_3978	component of the endoplasmic reticulum- associated degradation (ERAD) pathway	13.72	10.66
MRET_3979	golgi phosphoprotein 3	35.73	59.34
MRET_3980	uncharacterized protein	7.35	12.48
MRET_3981	U3 small nucleolar RNA-associated protein 22	10.96	17.8
MRET_3982	ankyrin repeat domain protein	33.5	46.65
MRET_3983	uncharacterized protein	389.46	154.07
MRET_3984	uncharacterized protein	162.15	185.18
MRET_3985	acetolactate synthase I/II/III large subunit	17.65	31.4
MRET_3986	cyclin	134.26	90.69
MRET_3987	tuftelin-interacting protein 11	12.21	15.43
MRET_3988	uncharacterized protein	65.9	72.2
MRET_3989	establishment of cell polarity	31.5	31.53
MRET_3990	separase	260.68	61.41
MRET_3991	uncharacterized protein	47.37	29.44
MRET_3992	conserved hypothetical protein	37.6	47.02
MRET_3993	protein transport protein SEC24	123.84	166.27

MRET_3994	GDSL-like lipase/acylhydrolase	38.95	63.05
MRET_3995	Got1 family protein	127.7	245.43
MRET_3996	cyclin-dependent kinase 8/11	34.42	77.43
MRET_3997	translation initiation factor eIF-2B subunit alpha	58.95	71.69
MRET_3998	ATP-dependent RNA helicase DDX27	31.29	102.68
MRET_3999	FHA domain protein	919.9	544.84
MRET_4000	ribosomal RNA assembly protein	1250.88	302.64
MRET_4001	SNF2 family helicase	19.74	48
MRET_4002	geranylgeranyl transferase type-1 subunit beta	46.71	96.51
MRET_4003	ATP-binding cassette, subfamily E, member 1	28.11	68.17
MRET_4004	uncharacterized protein	42.33	581.74
MRET_4005	RING-14 protein	88.32	152.12
MRET_4006	coatomer subunit beta	77.99	98.43
MRET_4007	peptide chain release factor 1	75.48	43.78
MRET_4008	SAGA-associated factor 29	45.37	81.04
MRET_4009	uncharacterized protein	628	486.92
MRET_4010	uncharacterized protein	294.5	142.51
MRET_4011	metallo-beta-lactamase domain protein	103.02	61.55
MRET_4012	enolase	502.85	648.62
MRET_4013	insulysin	225.48	211.52
MRET_4014	lariat debranching enzyme	177.62	96.25
MRET_4015	fatty acid desaturase	87.64	86.37
MRET_4016	cytochrome b5-like heme/steroid binding domain protein	132.06	154.91
MRET_4017	uncharacterized protein	29.64	59.39
MRET_4018	H/ACA ribonucleoprotein complex non-core subunit NAF1	50.67	50.76
MRET_4019	malate synthase	853.05	591.97
MRET_4020	SIT4-associating protein SAP185/190	29.52	70.43
MRET_4021	acetyl-CoA acyltransferase 2	387.28	239.39
MRET_4022	conserved oligomeric golgi complex subunit 5	68.36	136.02
MRET_4023	uncharacterized protein	69.9	166.35
MRET_4024	uncharacterized protein	70.53	69.87
MRET_4025	hydroxymethylpyrimidine/phosphomethylpyrimidine kinase/thiaminase	46.38	50.28
MRET_4026	AP-3 complex subunit beta	10.35	16.65
MRET_4027	essential nuclear protein 1	51.41	59.44
MRET_4028	molecular chaperone HtpG	3458.52	5200.28
MRET_4029	CCR4-NOT transcription complex subunit 6	132.46	240.35
MRET_4030	uncharacterized protein	16.67	28.69

MRET_4031	chitin synthase	79.39	41.27
MRET_4032	lipase	1216.78	653
MRET_4033	nitrosoguanidine resistance protein	457.74	103.87
MRET_4034	structural maintenance of chromosomes protein	45.04	70.95
MRET_4035	conserved oligomeric golgi complex subunit 8	11.05	34.11
MRET_4036	uncharacterized protein	62.6	86.71
MRET_4037	uncharacterized protein	85.82	306.72
MRET_4038	trehalose 6-phosphate synthase/phosphatase	58.89	168
MRET_4039	mannosyl-oligosaccharide alpha-1,2-mannosidase	53.71	85.56
MRET_4040	AP-2 complex subunit mu-1	21.69	35.21
MRET_4041	tRNA-splicing endonuclease subunit Sen54	30.46	39.58
MRET_4042	calcium permeable stress-gated cation channel	67.75	89.27
MRET_4043	nucleolar protein 6	87.05	132.95
MRET_4044	uncharacterized protein	186.98	213.55
MRET_4045	oxidoreductase	80.39	79.6
MRET_4046	cytosol aminopeptidase	138.51	127.88
MRET_4047	DNA excision repair protein ERCC-4	9.31	34.1
MRET_4048	uncharacterized protein	3536.03	2038
MRET_4049	uncharacterized protein	434.26	223.35
MRET_4050	MFS transporter, DHA1 family, multidrug resistance protein	227.07	120.03
MRET_4051	secreted protein	178.86	208.13
MRET_4052	DNA replication licensing factor MCM6	17.45	28.01
MRET_4053	uncharacterized protein	61.98	35.8
MRET_4054	ribosomal RNA-processing protein 7	72.07	89.16
MRET_4055	small subunit ribosomal protein S35	46.29	61.05
MRET_4056	putative methyltransferase	131.53	97.3
MRET_4057	mitochondrial import inner membrane translocase subunit TIM50	257.28	225.77
MRET_4058	acetylornithine aminotransferase	142.8	128.87
MRET_4059	glyoxylate reductase	243.46	161.44
MRET_4060	uncharacterized protein	29.3	18.13
MRET_4061	peptidyl-tRNA hydrolase domain 1	70.09	92.05
MRET_4062	trafficking protein particle complex subunit 4	47.57	92.58
MRET_4063	translation initiation factor 3 subunit M	24.18	83.04
MRET_4064	actin related protein 2/3 complex, subunit 3	103.4	101.21
MRET_4065	ribosome biogenesis ATPase	102.13	153.14
MRET_4066	tRNA-specific adenosine deaminase 2	108.05	223.4
MRET_4067	arginase	57.1	115.35

MRET_4068	cytochrome b5	221.79	95.21
MRET_4069	glutathione S-transferase	2143.6	1569.59
MRET_4070	glycoside hydrolase family 55 protein	60.82	108.62
MRET_4071	conserved hypothetical protein	53.78	96.55
MRET_4072	DUF159 domain protein	24.13	102.91
MRET_4073	MFS sugar transporter	124.13	144.08
MRET_4074	23S rRNA (uridine2552-2'-O)-methyltransferase	132.27	262.42
MRET_4075	protein-lysine N-methyltransferase EEF2KMT	26.04	48.48
MRET_4076	DnaJ homolog subfamily A member 5	7.51	18.41
MRET_4077	uncharacterized protein	19.96	44.77
MRET_4078	NADH dehydrogenase (ubiquinone) 1 alpha subcomplex subunit 8	174.64	297.12
MRET_4079	uncharacterized protein	82.55	152.95
MRET_4080	uncharacterized protein	95.89	72.92
MRET_4081	succinate-semialdehyde dehydrogenase/glutarate-semialdehyde dehydrogenase	408.18	300.84
MRET_4082	PA domain protein	69.64	129.33
MRET_4083	Sec14 cytosolic factor	322.01	417.66
MRET_4084	N-alpha-acetyltransferase 10/11	120.85	456.63
MRET_4085	uncharacterized protein	60.02	140.56
MRET_4086	nucleolar GTP-binding protein	29.07	124.65
MRET_4087	uncharacterized protein	192.1	194.16
MRET_4088	zinc finger protein	330.82	239.98
MRET_4089	1-pyrroline-5-carboxylate dehydrogenase	32.05	40.32
MRET_4090	trafficking protein particle complex subunit 3	81.24	163.68
MRET_4091	axial budding pattern protein 2	15.95	31.59
MRET_4092	nucleoporin GLE1	12.51	26.64
MRET_4093	N-glycosylase/DNA lyase	55.48	102.24
MRET_4094	SNF2 family helicase ATPase	58.74	160.59
MRET_4095	uncharacterized protein	218.07	223.11
MRET_4096	secreted aspartic endopeptidase	19.15	50.7
MRET_4097	uncharacterized protein	404.85	852.34
MRET_4098	secretory lipase	198.23	173.22
MRET_4099	secretory lipase	40.7	71.02
MRET_4100	Jumonji domain protein	293.53	159.42
MRET_4101	protein RER1	53.77	81.62
MRET_4102	ubiquitin carboxyl-terminal hydrolase 25	52.17	34.32
MRET_4103	HEAT repeat protein	723.48	61.05
MRET_4104	NLR family carD domain protein 3	60.45	41.08

MRET_4105	mannosyl-oligosaccharide alpha-1,2-mannosidase	18.47	23.83
MRET_4106	pyrimidine and pyridine-specific 5'-nucleotidase	149.14	142.2
MRET_4107	beta-1,4-N-acetylglucosaminyltransferase	17.65	74.07
MRET_4108	GTPase activating protein	178.59	247.68
MRET_4109	alkyl hydroperoxide reductase 1	1981.97	1280.57
MRET_4110	uncharacterized protein	262.45	507.35
MRET_4111	ISM6 protein	59.26	308.86
MRET_4112	DnaJ homolog subfamily C member 11	35.3	63.66
MRET_4113	phosphoadenosine phosphosulfate reductase	103.87	473.06
MRET_4114	large subunit ribosomal protein L4e	234.29	685.34
MRET_4115	carboxylesterase family	62.01	77.16
MRET_4116	thioredoxin reductase	1846.87	1048.86
MRET_4117	aconitate hydratase	154.06	43.42
MRET_4118	aconitase	76.72	228.04
MRET_4119	Hsp90 binding co-chaperone (Sba1)	1038.11	1757.99
MRET_4120	nucleolar protein 15	188.06	428.88
MRET_4121	type II protein arginine methyltransferase	24.04	44.36
MRET_4122	uncharacterized protein	12.55	30.9
MRET_4123	N-alpha-acetyltransferase 50	25.66	74.12
MRET_4124	actin-related protein 3	372.26	460.75
MRET_4125	phosphoinositide-3-kinase, regulatory subunit 4	49.01	38.18
MRET_4126	nucleolar protein 16	262.27	382.81
MRET_4127	retrotransposon	77.12	421.52
MRET_4128	FAD synthetase	30.6	60.56
MRET_4129	5'-3' exoribonuclease 1	36.95	50.82
MRET_4130	ribosome biogenesis protein NSA1	69.56	43.62
MRET_4131	cullin-associated NEDD8-dissociated protein 1	136.91	33.44
MRET_4132	homoserine O-acetyltransferase	275.09	89.48
MRET_4133	uncharacterized protein	35.57	29.73
MRET_4134	transcription elongation regulator 1	22.15	26.14
MRET_4135	uncharacterized protein	72.55	44.62
MRET_4136	vacuolar protein 8	339.1	352.85
MRET_4137	cytochrome c oxidase subunit 6a	493.32	396.85
MRET_4138	uncharacterized protein	684	310.27
MRET_4139	short-chain dehydrogenase reductase	359.67	438.94
MRET_4140	Sec20 domain protein	87.6	69.85
MRET_4141	Rab6A-GEF complex partner protein 2	69.69	69.34

MRET_4142	replication factor C subunit 1	57.91	83.41
MRET_4143	member of the PUF protein family	78.85	95.75
MRET_4144	alpha/beta-hydrolase lipase	553.97	498.9
MRET_4145	zinc finger protein, C2H2 type	553.04	597.12
MRET_4146	bromodomain factor 1	258.67	241.28
MRET_4147	trafficking protein particle complex subunit 6	99.16	64.35
MRET_4148	E3 ubiquitin-protein ligase UBR1	80.68	77.6
MRET_4149	E3 ubiquitin-protein ligase HUWE1	88.76	146.61
MRET_4150	MAPEG family protein	1796.92	796.92
MRET_4151	serine/threonine-protein kinase	70.41	187.8
MRET_4152	kinetochore protein Spc25, fungi type	26.92	96.32
MRET_4153	aarF domain kinase	30.93	73.19
MRET_4154	MFS multidrug transporter	45.92	42.1
MRET_4155	conserved hypothetical protein	297.15	164.41
MRET_4156	delta3,5-delta2,4-dienoyl-CoA isomerase	105.02	95.56
MRET_4157	elongator complex protein 1	166.8	51.82
MRET_4158	valyl-tRNA synthetase	27.23	60.75
MRET_4159	histone H1/5	22.53	403.97
MRET_4160	regulator of chromosome condensation	84.57	369.04
MRET_4161	peptidyl-prolyl cis-trans isomerase	2389.52	1762.06
MRET_4162	regulator of Ty1 transposition protein 109	84.37	56.62
MRET_4163	DNA mismatch repair protein	31.47	21.34
MRET_4164	uncharacterized protein	51.31	46.37
MRET_4165	phosphatidate phosphatase LPIN	244.83	144.31
MRET_4166	uncharacterized protein	431.71	196.3
MRET_4167	unfolded protein response protein Orm1	1798.7	1241.77
MRET_4168	pre-rRNA-processing protein TSR1	49.83	107.09
MRET_4169	26S proteasome regulatory subunit N8	71.26	120.02
MRET_4170	uncharacterized protein	84.04	63.6
MRET_4171	RNA-binding protein PNO1	22.19	40.05
MRET_4172	protein YOP1	294.51	505.92
MRET_4173	molecular chaperone GrpE	464.85	665.07
MRET_4174	polyadenylate-binding protein	817.1	702.74
MRET_4175	nucleolar complex protein 3	39.06	44.4
MRET_4176	carboxypeptidase D	62.94	67.41
MRET_4177	uncharacterized protein	69.27	57.12
MRET_4178	NFU1 iron-sulfur cluster scaffold homolog, mitochondrial	1756.25	758.99

MRET_4179	DNA-directed RNA polymerase I subunit RPA2	154.81	101.35
MRET_4180	protein transport protein YIF1	65.63	72.66
MRET_4181	translation initiation factor eIF1A	102.54	163.27
MRET_4182	paired amphipathic helix protein Sin3a	83.46	90.42
MRET_4183	dolichyl-phosphate beta-glucosyltransferase	65.5	73.83
MRET_4184	uncharacterized protein	61.69	99.83
MRET_4185	uncharacterized protein	95.21	329.55
MRET_4186	serine/threonine-protein kinase	38.45	138.7
MRET_4187	histone H2A	1047.07	1245.13
MRET_4188	WD domain, G-beta repeat protein	122.06	59.07
MRET_4189	uncharacterized protein	311.09	153.96
MRET_4190	mitochondrial import receptor subunit TOM40	100.51	141.68
MRET_4191	PHD finger domain protein	175.78	151.71
MRET_4192	osomolarity two-component system, sensor histidine kinase NIK1	67.58	76.44
MRET_4193	helix-loop-helix DNA-binding domain protein	25.31	62.02
MRET_4194	DNA topoisomerase III	48.77	134.34
MRET_4195	diphthine methyl ester acylhydrolase	32.86	103.15
MRET_4196	meiotic recombination protein SPO11	43.41	79.31
MRET_4197	diacylglycerol diphosphate phosphatase/phosphatidate phosphatase	54.37	199.99
MRET_4198	mitochondrial ABC transporter ATM	54.44	455.95
MRET_4199	nitrogen regulatory protein	35.89	215.07
MRET_4200	ADP-ribosylation factor-like protein 1	29.52	76.06
MRET_4201	COPII coat assembly protein SEC16	347.57	242.5
MRET_4202	peptide chain release factor subunit 1	42.71	145.25
MRET_4203	large subunit ribosomal protein L43	42.9	105.3
MRET_4204	cytokinesis protein	15.18	23.36
MRET_4205	V-type H+-transporting ATPase subunit D	95.69	212.87
MRET_4206	protein FAM32A	28.46	67.01
MRET_4207	UMP-CMP kinase	47.7	96.39
MRET_4208	protein BTN	17.18	19.46
MRET_4209	charged multivesicular body protein 2A	16.11	36.99
MRET_4210	mitogen-activated protein kinase kinase 2	61.79	74.11
MRET_4211	cytochrome c heme-lyase	79.85	126.59
MRET_4212	arabinose-5-phosphate isomerase	89.58	121.3
MRET_4213	YTH domain family protein	45.75	43.75
MRET_4214	cytoskeleton-associated protein 5	50.93	41.53
MRET_4215	permease	108.91	81.68

MRET_4216	homocitrate synthase	1238.74	560.06
MRET_4217	uncharacterized protein	67.22	129.28
MRET_4218	U4/U6.U5 tri-snRNP-associated protein 3	66.29	124.57
MRET_4219	uncharacterized protein	17.81	22.67
MRET_4220	tRNA-dihydrouridine synthase 2	10.86	20.7
MRET_4221	pentafunctional AROM polypeptide	163.26	80.01
MRET_4222	ATP-dependent permease	303.8	178.88
MRET_4223	cardiolipin synthase	90.65	58.3
MRET_4224	translation initiation factor eIF-2B subunit gamma	39.41	51.64
MRET_4225	regulator of nonsense transcripts 2	74.79	66.43
MRET_4226	heterogeneous nuclear rnp K-like protein	107.65	182.72
MRET_4227	small plasma membrane protein	64.05	65.61
MRET_4228	diacylglycerol acyltransferase family	73.31	156.39
MRET_4229	diacylglycerol acyltransferase family	74.69	131.1
MRET_4230	cytochrome p450	325.54	380.36
MRET_4231	dUTP pyrophosphatase	205.96	167.32
MRET_4232	putative methyltransferase	319.05	276.53
MRET_4233	peptidyl-prolyl cis-trans isomerase SDCCAG10	75.17	93.51
MRET_4234	RalA-binding protein 1	17.6	21.43
MRET_4235	Ran-binding protein 3	75.35	134.1
MRET_4236	universal stress protein	82.92	87.45
MRET_4237	ATP-dependent Lon protease	610.4	335.39
MRET_4238	DNA polymerase delta subunit 1	79.7	84.54
MRET_4239	25S rRNA (cytosine2870-C5)-methyltransferase	120.05	432.51
MRET_4240	RHO1 GDP-GTP exchange protein 1/2	168.82	73.04
MRET_4241	calcium permeable stress-gated cation channel	166.87	87.15
MRET_4242	NAD(P)H-hydrate epimerase	458.23	214.34
MRET_4243	flavin-binding monooxygenase-like protein	107.29	113.31
MRET_4244	putative vacuolar membrane transporter for cationic amino acids	446.98	356.35
MRET_4245	amyloid beta (A4) precursor protein-binding, family B, member 1 interacting protein	122.35	89.98
MRET_4246	DNA polymerase gamma 1	40.44	33.87
MRET_4247	nuclear envelope organization	52.51	39.89
MRET_4248	tyrosine phosphatase family	104.45	110.29
MRET_4249	SUN domain protein 1/2	40.84	34.76
MRET_4250	nucleoside-diphosphate kinase	76.87	220.38
MRET_4251	L-glyceraldehyde reductase	781.92	609.94
MRET_4252	ER membrane protein SH3	160.55	113.44

MRET_4253	zinc finger protein	572.39	265.04
MRET_4254	DNA excision repair protein ERCC-3	21.3	35.1
MRET_4255	dynactin 4	30.55	37.32
MRET_4256	actin-interacting protein	95.08	120.41
MRET_4257	DUF500 domain protein	22.37	58.31
MRET_4258	Rho guanyl nucleotide exchange factor	32.66	39.81
MRET_4259	chitin biosynthesis protein CHS5	33.42	49.81
MRET_4260	Vps51/Vps67 family protein	39.62	44.11
MRET_4261	transcription initiation factor TFIIF subunit alpha	244.03	126.16
MRET_4262	protein involved in negative regulation of iron regulon transcription	34.93	33.77
MRET_4263	TPR repeat protein	26.89	34.75
MRET_4264	cell division control protein 7	71.87	61.49
MRET_4265	uncharacterized protein	72.84	89.57
MRET_4266	TP53 regulating kinase and related kinases	92.65	95.1
MRET_4267	chromatin modification-related protein	253.66	238.26
MRET_4268	DNA mismatch repair protein MSH2	144.95	141.34
MRET_4269	uncharacterized protein	105.04	65.39
MRET_4270	protein transport protein SEC9	36.21	56.68
MRET_4271	mitochondrial inner membrane protease ATP23	17.23	39.59
MRET_4272	large subunit ribosomal protein L13e	234.19	742.56
MRET_4273	Ras GTPase-activating-like protein IQGAP2/3	22.02	93.43
MRET_4274	serine/threonine-protein kinase	58.63	56.36
MRET_4275	AT rich DNA binding protein	44.42	39.17
MRET_4276	uncharacterized protein	70.1	83.62
MRET_4277	sphingomyelin phosphodiesterase	112.02	87.03
MRET_4278	2-phosphoxylose phosphatase	271.65	148.85
MRET_4279	aryl-alcohol dehydrogenase	1125.54	592.91
MRET_4280	ion channel regulatory protein UNC-93	62.31	17.27
MRET_4281	eukaryotic aspartyl protease	40.11	40.44
MRET_4282	eukaryotic aspartyl protease	15.78	22.7
MRET_4283	uncharacterized protein	378.51	466.49
MRET_4284	glucose oxidase	28.66	41.79
MRET_4285	DnaJ-related protein SCJ1	49.3	67.38
MRET_4286	eukaryotic aspartyl protease	55.49	78.14
MRET_4287	cathepsin D	158.62	125.26
MRET_4288	mitochondrial import inner membrane translocase subunit TIM10	92.08	217.95
MRET_4289	small subunit ribosomal protein S10e	39.22	162.48

MRET_4290	protein of unknown function (DUF2034)	85.73	65.22
MRET_4291	DASH complex subunit DAD2	198.28	131.64
MRET_4292	uncharacterized protein	269.68	165.36
MRET_4293	SAGA-associated factor 73	148.5	65.43
MRET_4294	ATP-dependent RNA helicase DDX35	89.63	49.69
MRET_4295	charged multivesicular body protein 7	76.9	44.67
MRET_4296	v-SNARE component of the vacuolar SNARE complex	202.45	109
MRET_4297	aspartyl-tRNA synthetase	60.21	39.84
MRET_4298	2-dehydropantoate 2-reductase	105.33	103.07
MRET_4299	translation machinery associated TMA7	458.55	139.61
MRET_4300	transcription factor	33.05	64.66
MRET_4301	methionyl-tRNA synthetase	97.48	93.42
MRET_4302	uncharacterized protein	175.57	107.29
MRET_4303	sorting and assembly machinery component 37	169.25	94.27
MRET_4304	uncharacterized protein	138.62	109.49
MRET_4305	short-chain dehydrogenase	88.82	139.13
MRET_4306	NADH dehydrogenase (ubiquinone) flavoprotein 2	1404.71	1125.35
MRET_4307	uncharacterized protein	28.34	36.74
MRET_4308	uncharacterized protein	341.93	229.31
MRET_4309	fungal protein of unknown function (DUF1748)	140	94.24
MRET_4310	ribonuclease H2 subunit B	24.67	24.72
MRET_4311	uncharacterized protein	201.75	166.74
MRET_4312	NADH dehydrogenase (ubiquinone) Fe-S protein 8	232.19	344.07
MRET_4313	anaphase-promoting complex subunit 3	21.21	39.89
MRET_4314	cytosolic Fe-S cluster assembly factor CFD1	155.08	85.43
MRET_4315	adenylate kinase	163.51	139.09
MRET_4316	DNA repair protein REV1	146.35	64.84
MRET_4317	mitofilin	414.72	261.77
MRET_4318	3,4-dihydroxy 2-butanone 4-phosphate synthase	1217.5	526.9
MRET_4319	V-type H+-transporting ATPase subunit A	461.45	320.42
MRET_4320	26S proteasome regulatory subunit T1	373.28	212.9
MRET_4321	coiled-coil domain protein 75	44.06	46.44
MRET_4322	acetyl-CoA C-acetyltransferase	112.86	178.49
MRET_4323	ubiquitin-conjugating enzyme E2 variant	245.03	210.15
MRET_4324	exosome complex component RRP4	195.35	87.62
MRET_4325	pre-mRNA-splicing factor CWC22	407.81	106.65
MRET_4326	DNA repair protein RAD7	209.85	82.74

MRET_4327	actin-related protein 10	180.88	99.36
MRET_4328	pre-mRNA-splicing factor SYF2	651	598.31
MRET_4329	regulator of nonsense transcripts 3	154.19	80.46
MRET_4330	ubiquitin-protein ligase involved in ER-associated protein degradation	298.27	174.54
MRET_4331	proline-rich receptor-like protein kinase	50.44	51.63
MRET_4332	uncharacterized protein	48.58	52.21
MRET_4333	lipase esterase family protein	47.39	52.3
MRET_4334	cytochrome-b5 reductase	41.27	21.18
MRET_4335	saccharopine dehydrogenase (NAD+, L-lysine forming)	261.73	125.93
MRET_4336	Ran-interacting Mog1 protein	29.52	54.38
MRET_4337	uncharacterized protein	43.33	35.4
MRET_4338	pre-rRNA-processing protein IPI3	108.47	107.39
MRET_4339	asparagine synthase (glutamine-hydrolysing)	196.07	106.16
MRET_4340	mitochondrial import inner membrane translocase subunit TIM16	847.41	770.43
MRET_4341	OmpA-like domain protein	79.61	85.64
MRET_4342	spinocerebellar ataxia type 10 protein domain protein	81.15	94.28
MRET_4343	protein SIP5	265.98	131.89
MRET_4344	cell division control protein 42	195.5	325.92
MRET_4345	uncharacterized protein	132.14	136.89
MRET_4346	centromeric DNA binding protein	87.03	65.62
MRET_4347	conserved hypothetical protein	38.9	31.59
MRET_4348	integrin alpha FG-GAP repeat containing protein 1	317.16	143.76
MRET_4349	V-type H+-transporting ATPase subunit a	319.97	279.17
MRET_4350	adenylate kinase	19.27	23.26
MRET_4351	uncharacterized protein	11.58	16.57
MRET_4352	splicing factor 3A subunit 1	140.11	61.1
MRET_4353	anthranilate synthase component I	66.76	55.05
MRET_4354	uncharacterized protein	50.63	203.02
MRET_4355	uncharacterized protein	772.19	371.64
MRET_4356	lipase	124.99	91.15
MRET_4357	glycine cleavage system H protein	206.25	350.5
MRET_4358	DNA-dependent metalloprotease WSS1	158.33	207.94
MRET_4359	cohesin complex subunit SA-1/2	27.24	29.97
MRET_4360	AHNAK nucleoprotein	53.76	51.2
MRET_4361	uncharacterized protein	118.86	233.93
MRET_4362	ubiquitin-conjugating enzyme E2 L3	212.47	232.63
MRET_4363	uncharacterized protein	96.09	145.06

MRET_4364	tRNA(His) guanylyltransferase	89.35	109.54
MRET_4365	peroxin-12	97.04	65.67
MRET_4366	ubiquitin carboxyl-terminal hydrolase 5/13	136.15	84.91
MRET_4367	adenylyl cyclase-associated protein	72.95	48.47
MRET_4368	kinetochore protein NDC80	63.44	83.28
MRET_4369	ribonucleoside-diphosphate reductase subunit M2	1100.78	683.8
MRET_4370	YL1 nuclear protein	222.25	82.52
MRET_4371	diacylglycerol acyltransferase family	106.78	115.38
MRET_4372	BRCT domain protein	43.79	26.77
MRET_4373	S-adenosylmethionine-dependent methyltransferase	27.95	19.11
MRET_4374	periodic tryptophan protein 1	35.92	45.41
MRET_4375	ribosome production factor 2	49.01	57.42
MRET_4376	membrane-associating domain protein	95.24	200.11
MRET_4377	ribonucleoside-diphosphate reductase subunit M1	851.17	408.65
MRET_4378	uncharacterized protein	236.35	144.83
MRET_4379	uncharacterized protein	69.33	61.58
MRET_4380	DUF833 domain protein	31.89	28.26
MRET_4381	DNA-directed RNA polymerase III subunit RPC11	652.44	256.08
MRET_4382	uncharacterized protein	666.62	440.35
MRET_4383	structure-specific recognition protein 1	140.82	146.8
MRET_4384	nicotinamide/nicotinate riboside kinase	31.13	55.49
MRET_4385	mitochondrial 37S ribosomal protein RSM19	76.5	146.3
MRET_4386	NAD+ diphosphatase	51.5	135.85
MRET_4387	pre-rRNA-processing protein IPI1	35.1	23.64
MRET_4388	MFS family protein	149.76	43.87
MRET_4389	septicolysin	999.28	1085.39
MRET_4390	eukaryotic aspartyl protease	8.43	28.01
