# Supplementary material for: A Novel Virus Alters Gene Expression and Vacuolar Morphology in Malassezia Cells and Induces a TLR3-Mediated Inflammatory Immune Response
Source: mBio. 2020 Sep 1;11(5):e01521-20. doi: 10.1128/mBio.01521-20 (PMC7468201; doi:10.1128/mBio.01521-20)
Supplement: FIG S3 [file mBio.01521-20-sf003.pdf]

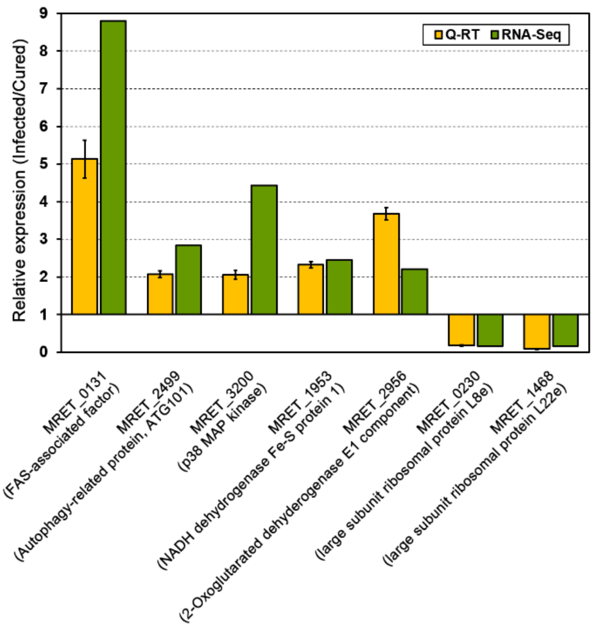

Fig. S3. Validation of the differential expression in transcriptome analysis. Differential expression of selected genes was confirmed by Q-RT PCR. MRET_0131 (FAS-associated factor); MRET_2499 (Autophagy-related protein, ATG101); MRET_3200 (p38 MAP kinase); MRET_1953 (NADH dehydrogenase Fe-S protein 1); MRET_2956 (2-Oxoglutarated dehyderogenase E1 component); MRET_0230 (large subunit ribosomal protein L8e); MRET_1468 (large subunit ribosomal protein L22e).
