# Supplementary material for: A Novel Virus Alters Gene Expression and Vacuolar Morphology in Malassezia Cells and Induces a TLR3-Mediated Inflammatory Immune Response
Source: mBio. 2020 Sep 1;11(5):e01521-20. doi: 10.1128/mBio.01521-20 (PMC7468201; doi:10.1128/mBio.01521-20)
Supplement: FIG S4 [file mBio.01521-20-sf004.pdf]

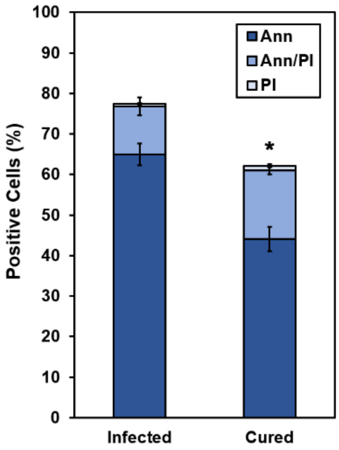

Fig. S4. Evaluation of apoptosis in *M. restricta* strains. The virus-infected and the virus-cured *M. restricta* KCTC 27540 cells were stained with Annexin V and propidium iodide (PI) to evaluate apoptosis. Annexin V binds to phosphatidylserine (PS), which translocates from the inner leaflet of the plasma membrane to the outer leaflet upon apoptosis while the plasma membrane integrity is still maintained (1). PI is permeable when cell membrane was ruptured by damage or upon cell death (2). Overall apoptosis was quantified by Annexin V(Ann), and early and late apoptosis was quantified by Annexin V(Ann)/PI and PI, respectively. Statistical analysis for differences between the strains was performed with unpaired t-tests. *p < 0.01.

1. Martin S, Reutelingsperger C, McGahon AJ, Rader JA, Van Schie R, LaFace DM, Green DR. 1995. Early redistribution of plasma membrane phosphatidylserine is a general feature of apoptosis regardless of the initiating stimulus: inhibition by overexpression of Bcl-2 and Abl. The Journal of experimental medicine 182:1545-1556.

2. Carmona-Gutierrez D, Bauer MA, Zimmermann A, Aguilera A, Austriaco N, Ayscough K, Balzan R, Bar-Nun S, Barrientos A, Belenky P. 2018. Guidelines and recommendations on yeast cell death nomenclature. Microbial Cell 5:4.
