# Supplementary material for: A Novel Virus Alters Gene Expression and Vacuolar Morphology in Malassezia Cells and Induces a TLR3-Mediated Inflammatory Immune Response
Source: mBio. 2020 Sep 1;11(5):e01521-20. doi: 10.1128/mBio.01521-20 (PMC7468201; doi:10.1128/mBio.01521-20)
Supplement: TABLE S2 [file mBio.01521-20-st002.pdf]

Table S2. Primers used for sequencing and heterologous expression of viral proteins

Primer	Sequence (5’-3’)
Tagged dN_6_1	CCTGAATTCGGATCCTCCNNNNNN
Tagged oligo_1	CCTGAATTCGGATCCTCC
Linkage1_V40	CACACCTGACGAGTTGTATGACA
Linkage2_V40	GCTGGCTGTTAAAGTCATCATAATCG
V40L_SP1	CGAAATGCTCAATCGGGATGAAGC
V40L_SP2	GTCCATTCCATCGAGGGAAGTT
V40L_SP3	CTCACTACACAATGAGGCCATCG
V40L_SP4-3	CCTCGAGTGCCAGAACTGACAT
V40L_SP5-2	CATCGGCAGAGTGGACTCATGAC
V40L_SP6	CTCACTGCACAACGGTGATGATA
V40L_SP7	GAACAGAGTCTGAGGCGCCTA
V40L_SP8	GGACTACGTCAAAGAACGCTTCAA
V40L_SP9	CGGTATCGAGACTGACGAGAGAA
MrV40L_CP_F1	CTCGGTAGGGTTGATGAAACCTGT
MrV40L_CP_R1	CGCTTGAGGAAGTTTTCATACGAG
MrV40L_CP_F2	GTGATTTCGATCTAGCCTACCACA
MrV40L_CP_R2	ATTCCTCGACGCCGCCAGGACT
MrV40L_CP_F3	CTCGTATGAAAACTTCCTCAAGCG
MrV24L_CP_F2	GGCACAGGAAAGTATAAGCCTG
MrV24L_CP_R2	ATTCCTCGACGCCGCCAGGACT
MrV79L_CP_F1	GACGTATAAGCAGCATTTGGG
MrV79L_CP_R1	CGGTGAGTCCCAACTGATCCA
MrV40L_RDRP_F1	GATCACGCAACAGCATCATGTTCATG
MrV40L_RDRP_R1	CTCAGTATGCCAATCCACAGTGTC
MrV40L_RDRP_F2	GCCACCACTAGCACAAAATATG
MrV40L_RDRP_R2	TATCATCACCGTTGTGCAGTGAG
MrV40L_RDRP_F4	CCACTAGCACAAAATATGAGTGG
MrV50L_RDRP_F1	CGCATCCACTAGTACAAAGTATG
MrV50L_RDRP_R3	TCGTCACCATTGTGCAACGAG
MrV24L_RDRP_F1	GGGTCTGGGATGTTACACGCAA
MrV24L_RDRP_R1	CATCTTACTTAACTGTGCGCGC
V40S_SP1	GAATAGCTGCGAAGAGTCAAGCA
V40S_SP2	CATCGAATACTCGCTCCACAGC
V40S_SP3-2	GGGCTGAAAGTTGTCATAAGACC
V40S_SP4	CTGCATTTGCTTAGGTGAACATGG
V40S_SP5	CAGAGACGTATGCCAGTATGAA
V40S_SP6	CAGAGACCGGGTTCTGATAGT

MrV40S_F1	GCACTATCAGAACCCGGTCTCT
MrV40S_F2	CCTGCCACTGTGTTGTCAAGCAA
MrV40S_F3	GCTGTGGAGCGAGTATTCGATG
MrV40S_F5	GGTCTTATGACAACTTTCAGCCC
MrV40S_R1	GCACTATCAGAACCCGGTCTCT
MrV40S_ORF_F1	ATGAAGATATTTGACTACTTTAG
MrV40L.CP.F_BamHI	GATGGTGGATCCTCGTTTACGTTATTTG ATCAATTGACAGGTCC
MrV40L.CP.R_HindIII	GATTGGTAAGCTTTTAGTGTTCCGCCGG TGCACCAT
MrV40S.F_BamHI	GTTGGGGATCCATGAAGATATTTGACTA CTTTAGC
MrV40S.R_XbaI	CCTGCTCTAGAATCAGTTACGAATTGCA ATCCAACT
