# Supplementary material for: A Novel Virus Alters Gene Expression and Vacuolar Morphology in Malassezia Cells and Induces a TLR3-Mediated Inflammatory Immune Response
Source: mBio. 2020 Sep 1;11(5):e01521-20. doi: 10.1128/mBio.01521-20 (PMC7468201; doi:10.1128/mBio.01521-20)
Supplement: FIG S5 [file mBio.01521-20-sf005.pdf]

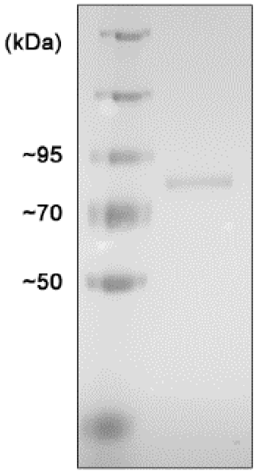

Fig. S5. Purification of the MrV40 capsid protein. The capsid protein encoded by ORF1 of MrV40 was heterologously expressed in *E. coli*, purified using a His-tag column, and used to analyze the expression of TLRs and cytokines in BMDCs.
