# Supplementary material for: A Novel Virus Alters Gene Expression and Vacuolar Morphology in Malassezia Cells and Induces a TLR3-Mediated Inflammatory Immune Response
Source: mBio. 2020 Sep 1;11(5):e01521-20. doi: 10.1128/mBio.01521-20 (PMC7468201; doi:10.1128/mBio.01521-20)
Supplement: TABLE S3 [file mBio.01521-20-st003.pdf]

Table S3. Primers used for Q-RT PCR

Target genes		Sequence (5′-3′)
MRET_0131	Forward	GGAGCATAAGCATGCACTTTTG
	Reverse	CCCATGCCCGCCATT
MRET_2499	Forward	GCTCGGCCTGCTCCAA
	Reverse	CGGCATCGATACGCCTTT
MRET_3200	Forward	TCCATCTTTGGGACCGTGTT
	Reverse	GCCCACAGGCTGCAAGTT
MRET_1953	Forward	TCGATGCGCTACGGTAAAGA
	Reverse	CACGCTTTCCAGTCGTCTCA
MRET_2956	Forward	TCCCATTGAGGGCATTCTG
	Reverse	CCACCGCCCTCTTCGTT
MRET_0230	Forward	TGAACAAGGCGCCTGCTAA
	Reverse	CCGTTGCGCTCAGCAAA
MRET_1468	Forward	TGATGCCGACGCTTTCG
	Reverse	GGACGACCATCGACCTTGAT
MRET_1518	Forward	CCTTCCTTGCCCTCTTCTCAT
	Reverse	AGCGACGACAGGGACAATG
TLR3	Forward	TTGCGTTGCGAAGTGAAGAA
	Reverse	TCAGTTGGGCGTTGTTCAAG
TLR7	Forward	ATATCCCAGAGGCCCATGTG
	Reverse	ACACACATTGGCTTTGGACC
TLR8	Forward	TCCTCCCTGCAAACCAAGAT
	Reverse	AAAACAGGACAGCTGCAGTG
TLR9	Forward	CCTGAAGTCTGTACCCCGTT
	Reverse	TCTGGGCTCAATGGTCATGT

β-actin	Forward	CCTCTATGCCAACACAGTGC
	Reverse	CCTGCTTGCTGATCCACATC
